# Molecular-level petroleum refining by graded membranes

**DOI:** 10.1093/nsr/nwag488

**Published:** 2026-08-12

**Authors:** Yuecheng Wang, Fang-Di Dong, Yujie Ban, Ziyi Hu, Dong-Dong Zhou, Jie-Peng Zhang, Xiao-Ming Chen, Weishen Yang

**Affiliations:** State Key Laboratory of Catalysis, Dalian Institute of Chemical Physics, Chinese Academy of Sciences, Dalian 116023, China; University of Chinese Academy of Sciences, Beijing 100049, China; MOE Key Laboratory of Bioinorganic and Synthetic Chemistry, GBRCE for Functional Molecular Engineering, School of Chemistry, IGCME, Sun Yat-Sen University, Guangzhou 510275, China; State Key Laboratory of Catalysis, Dalian Institute of Chemical Physics, Chinese Academy of Sciences, Dalian 116023, China; University of Chinese Academy of Sciences, Beijing 100049, China; State Key Laboratory of Catalysis, Dalian Institute of Chemical Physics, Chinese Academy of Sciences, Dalian 116023, China; MOE Key Laboratory of Bioinorganic and Synthetic Chemistry, GBRCE for Functional Molecular Engineering, School of Chemistry, IGCME, Sun Yat-Sen University, Guangzhou 510275, China; MOE Key Laboratory of Bioinorganic and Synthetic Chemistry, GBRCE for Functional Molecular Engineering, School of Chemistry, IGCME, Sun Yat-Sen University, Guangzhou 510275, China; MOE Key Laboratory of Bioinorganic and Synthetic Chemistry, GBRCE for Functional Molecular Engineering, School of Chemistry, IGCME, Sun Yat-Sen University, Guangzhou 510275, China; State Key Laboratory of Catalysis, Dalian Institute of Chemical Physics, Chinese Academy of Sciences, Dalian 116023, China; University of Chinese Academy of Sciences, Beijing 100049, China

**Keywords:** molecular refining, mild etching, metal-organic framework, graded membrane, light naphtha separation

## Abstract

Light naphtha is a critical feedstock whose full value relies on molecular-level fractionation into value-specific streams. Achieving such precise separation remains difficult because its components exhibit extremely similar physicochemical properties. Here, we address this challenge by developing a graded membrane cascade based on metal-organic framework CuBTC whose pore size and surface chemistry are continuously tuned through mild tannic acid treatment. Membranes with distinct sieving characteristics were created to sequentially co-separate linear and mono-branched alkanes and then selectively permeate aromatics. The integrated cascade fractionated a 15-component C_5_–C_7_ mixture into three high-purity streams, ethylene feedstock, chemical intermediates, and gasoline fuel, with recoveries of 85%–90% and an estimated 91% reduction in energy consumption compared to conventional distillation. This strategy establishes an energy-efficient route toward molecular refining.

## Teaser

A smart MOF membrane cascade enables energy-efficient, molecular-level refining of light naphtha, fractionating its complex mixture into three distinct value-added streams—ethylene feedstock, chemical intermediates, and clean gasoline.

## INTRODUCTION

Petroleum fractionation via distillation consumes ∼1% of global energy [1–5]. Light naphtha, a complex fraction derived from petroleum, consists mainly of C_5_–C_7_ hydrocarbons, including linear alkanes, branched isomers, cyclic alkanes, and aromatics (Table S1) [6–8]. As a versatile feedstock, light naphtha is integral to various chemical manufacturing processes. Thermal cracking of its linear alkanes yields ethylene, which constitutes ∼80% of global ethylene production (∼180 MMt/a) [9,10]. Its highly branched and cyclic alkanes, with research octane numbers (RON) typically exceeding 85, serve as anti-knock gasoline fuel (∼140 MMt/a) [11–13]. Meanwhile, aromatics such as BTX (benzene-toluene-xylene) are essential intermediates in producing plastics, resins, and fibers (∼120 MMt/a) [14–16]. Consequently, separating light naphtha at the molecular level is critical to maximizing the value derived from each component, representing a strategic approach in modern petrochemical industries [17,18]. However, achieving this precise separation requires significantly more theoretical distillation plates due to extremely similar physicochemical properties of hydrocarbons within such a narrow carbon number range.

Compared to conventional distillation, membrane-based separation technologies offer notable energy savings [19–24]. Traditional membranes primarily rely on size exclusion, exploiting the modest dimensional differences between linear alkanes and other molecules (Fig. S1) to selectively extract linear alkanes from the mixture [25–27]. Among these, metal-organic framework (MOF) membranes are particularly attractive because of their well-defined pore architectures. Precise control over pore size and orientation is crucial for enhancing the selectivity of linear alkanes over isomeric counterparts [26–28]. However, these methods often retain lightly branched alkanes (e.g. mono-branched isomers), which leads to underutilization of potential ethylene feedstock and simultaneously lowers the overall RON of the residual stream. Furthermore, conventional membranes are generally ineffective at separating aromatic hydrocarbons from aliphatic ones in light naphtha [14], a limitation that impedes comprehensive resource utilization and raises potential public health concerns. Fundamentals of molecular refining necessitate a specialized membrane platform for molecular-scale fractionation of complex light naphtha into diverse high-value streams, bridging the existing research gap.

To address these challenges, we developed a graded sorting paradigm based on the hierarchical molecular size and aromaticity differences among light naphtha components (Fig. 1), achieving complete utilization of all light naphtha fractions. This cascade process first co-separates linear and mono-branched alkanes by size sieving, targeting molecular dimension differences of 0.08–0.12 nm. It then isolates aromatics from the remaining fraction via specific chemical recognition, even when their size differences are as subtle as 0.01–0.03 nm. Unlike conventional membranes designed mainly for linear alkane extraction, graded sorting membranes must address a dual challenge: they need to differentiate among larger molecules with extremely fine size variations, sometimes as small as 0.01 nm. To achieve this goal, we implemented gentle etching of a MOF membrane using a tannic acid (TA) solution, a naturally derived polyphenol, at parts-per-million (ppm) concentration (Fig. S2). This mild etching process enabled continuous, precise modulation of both the sieving pore size and the surface functionality of the membrane, dynamically adapting it to the requirements of graded light naphtha sorting.

**Figure 1.**
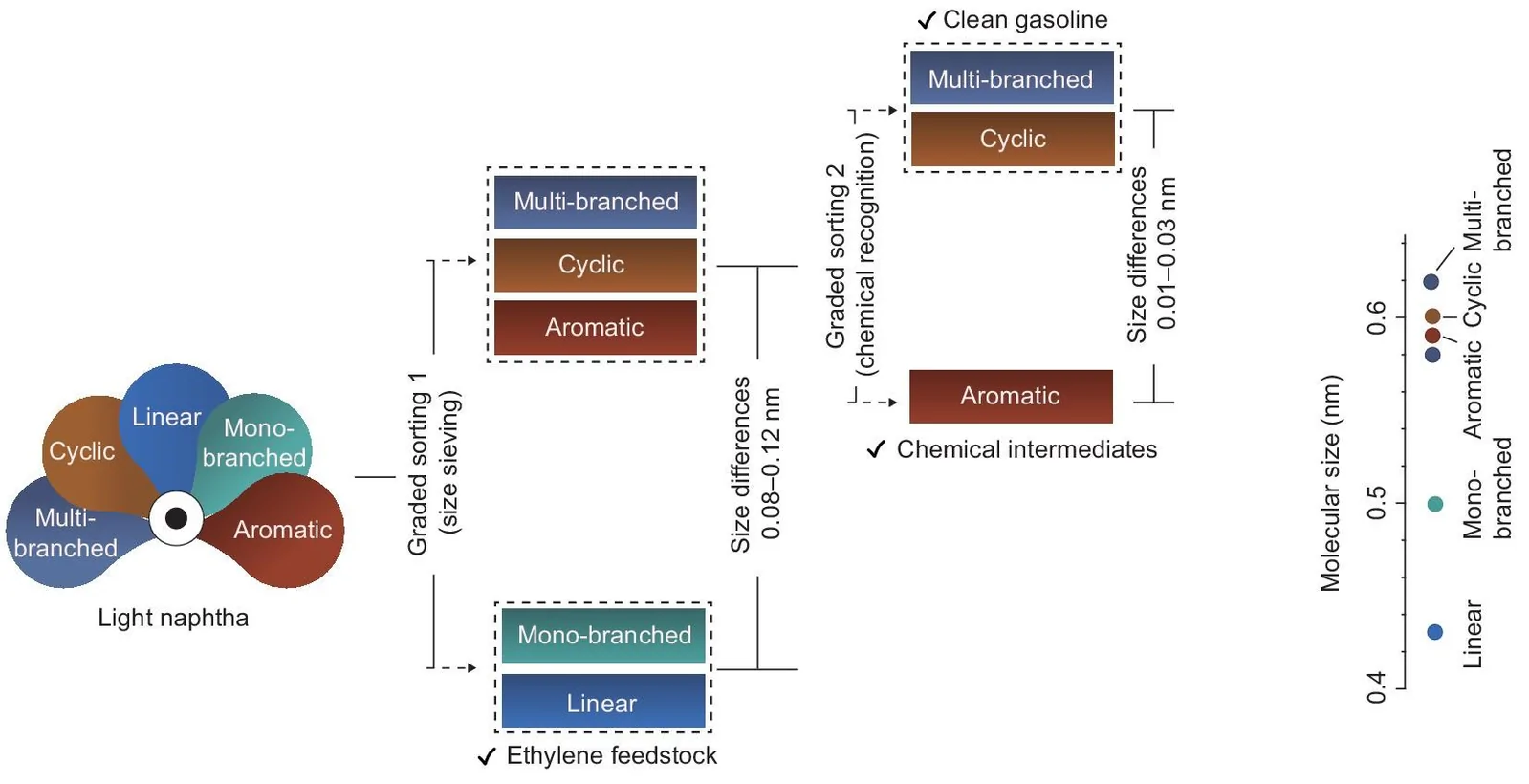
Schematic illustration of graded separation of light naphtha, using the C_6_ component as an example. The molecular size is defined in terms of its kinetic diameter.

## RESULTS AND DISCUSSION

### Mildly etching MOF membranes

CuBTC (BTC is the abbreviation for the 1,3,5-benzenetricarboxylate linker) membranes with a {111} orientation, consisting of well-interconnected octahedral crystals, were synthesized on α-alumina support disks (Fig. S3) via a static seeding-thermal conversion (SS-TC) method previously developed in our laboratory (Fig. S4) [26]. Powder X-ray diffraction (PXRD) showed a pronounced (222) peak at 2*θ* = 11.6° (Fig. S5), confirming the preferred orientation of the membrane. In this configuration, octahedral cages align perpendicular to the membrane plane, and their triangular windows serve as the primary sieving pores (Fig. S6).

We immersed the membranes in a mildly acidic TA-water solution (680 ppm) for 1 to 60 min, followed by thorough washing with ethanol. This TA treatment had minimal effect on membrane thickness (Fig. 2a). Cross-sectional scanning electron microscopy analysis further confirmed the overall preservation of structural integrity (Fig. 2b and Fig. S7). Given that the TA treatment partially disrupted the Cu-BTC coordination bonds via mild proton-induced cleavage [29], a reduction in the PXRD intensity of the membrane was observed (Fig. 2c and Fig. S8). Nonetheless, the crystalline framework of the membrane remained intact. To elucidate chemical compositional changes, we performed acid degradation of the membranes followed by analysis with inductively coupled plasma-atomic emission spectroscopy and proton nuclear magnetic resonance (^1^H NMR) spectroscopy (Figs S9 and S10). The pristine CuBTC membrane had a BTC/Cu molar ratio of 0.67, consistent with its crystallographic stoichiometry. TA treatments from 1 to 60 min progressively reduced the BTC content (Fig. 2d), which was concomitant with a gradual blue shift in the Cu-Cu vibrational mode [30] observed via Raman spectroscopy (Fig. 2e). Owing to their capacity for metal coordination [31], more TA molecules interacted with the MOF membrane over time (Fig. 2d), partially filling the linker vacancies generated. Fourier transform infrared spectroscopy further corroborated this interaction: it showed a time-dependent increase in the vibrational intensity of phenolic C–O groups in TA (Fig. 2f), along with a blue shift attributable to metal-phenol coordination (Fig. S11).

**Figure 2.**
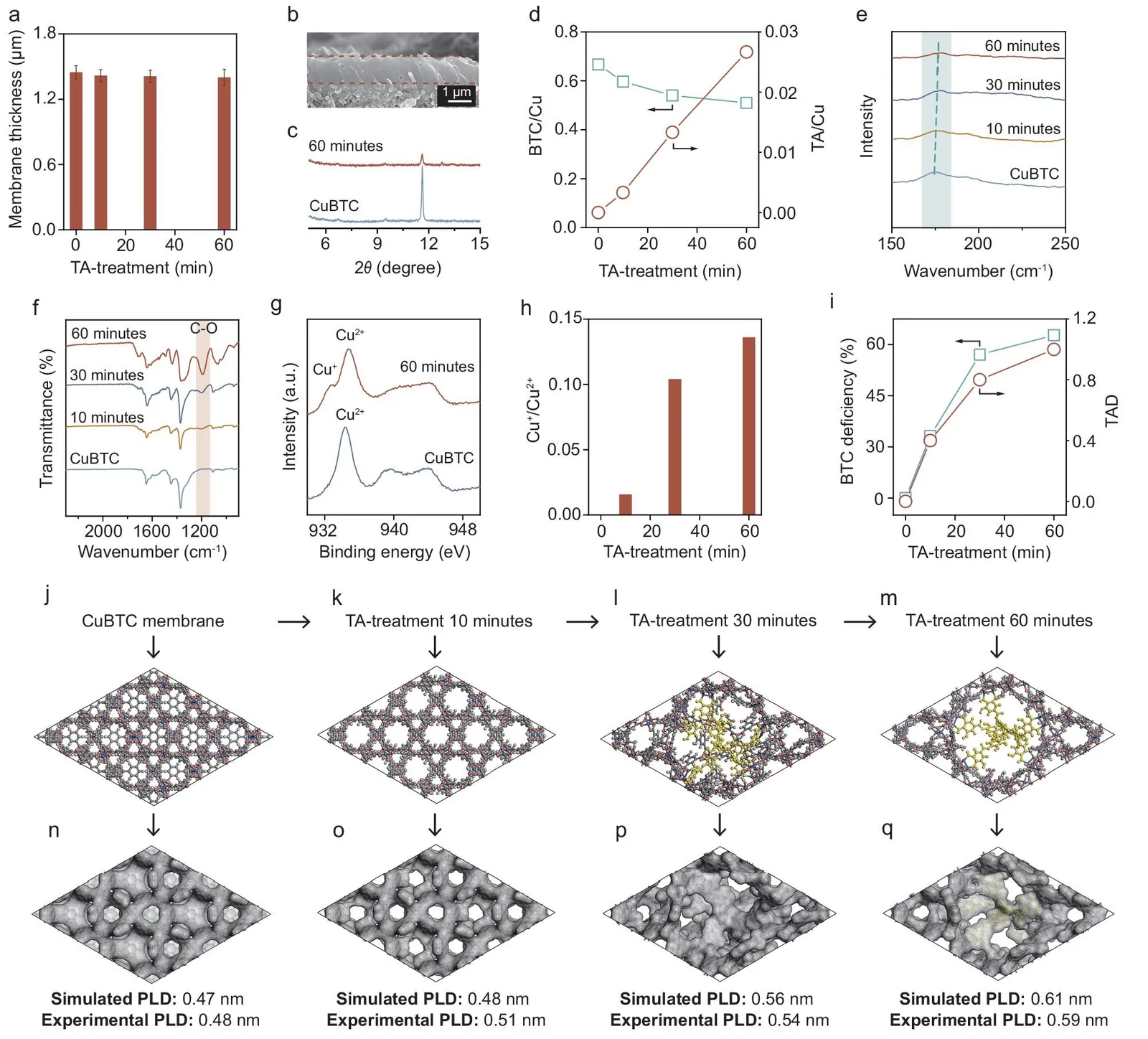
Structural evolution of the CuBTC membranes as a function of TA treatment duration. (a) Comparison of membrane thickness between the pristine CuBTC membrane and those subjected to TA treatment. (b) Representative cross-sectional scanning electron microscopy image of the CuBTC membrane following 60 min of TA treatment. (c) Comparison of PXRD patterns between the pristine CuBTC membrane and TA-treated CuBTC membranes. (d) Molar ratios of BTC/Cu and TA/Cu. (e) Raman spectra. (f) Fourier transform infrared spectra. (g) XPS spectra of both pristine and TA-treated CuBTC membranes. (h) Variation in the Cu^+^/Cu^2+^ molar ratio corresponding to different durations of TA treatment. (i) BTC deficiency ratio alongside TAD values. (j–m) Schematic models depicting BTC vacancies and TA coordination within individual slabs of CuBTC membranes treated with TA. TA molecules are highlighted in yellow in panels (l) and (m). (n–q) Models illustrating the simulated PLD of pristine and TA-treated CuBTC membranes, in comparison with the experimental data.

High-resolution X-ray photoelectron spectroscopy (XPS) revealed the coexistence of Cu^+^ and Cu^2+^ species in the TA-treated membranes, indicating that some Cu^2+^ nodes were reduced to Cu^+^ (Fig. 2g and Fig. S12). Prolonged TA exposure further increased the Cu^+^/Cu^2+^ molar ratio in the membrane (Fig. 2h). Ultimately, BTC linker depletion left the CuBTC membrane positively charged; it was neutralized by OH^–^, and bulky TA molecules remained stably embedded in the membrane matrix.

### Precisely editing sieving pore size and surface functionality

The continuous alterations in the membrane’s sieving pore size and functionality following TA etching are of particular significance. The BTC/Cu molar ratio was translated into the average number of BTC linkers per specific MOF slab to explore BTC deficiency as a function of TA treatment time (Fig. 2i) via systematic computational structure optimization (Fig. 2j–m; Figs S13–S15 and Table S2). This analysis indicated a BTC deficiency varying from ∼18% to 47% as the TA treatment was extended from 10 to 60 min (Fig. 2i). Such linker removal correspondingly reconfigured the sieving pore size of MOFs according to its intrinsic structural characteristics, resulting in a continuous broadening of the simulated pore limiting diameter (PLD) (Fig. 2n–q and Fig. S16).

Single-gas permeation experiments were performed with hydrogen, methane, ethane, propane, and *n*-butane as probe molecules to determine the experimental PLD of the membranes. The obtained permeation data were analyzed using a normalized Knudsen-based permeance method [32] for fitting these measurements (Methods; Tables S3–Table S6 and Fig. S17). As BTC linkers were progressively removed, the membrane PLD systematically expanded from 0.48 nm to 0.59 nm (denoted in Fig. 2n–q), consistent with the simulated results. These findings confirmed that mild TA treatment can continuously enlarge the sieving pores without compromising the structural integrity of the membrane.

Additionally, we estimated the TA density (TAD), the average number of TA molecules per MOF slab, to increase from about 0.4 to 1 as the TA treatment duration rose from 10 to 60 min (Fig. 2i). This suggests that each MOF slab incorporates, at most, approximately one TA molecule (Fig. 2m) via metal-phenol coordination (Figs S18, S11 and S19). Density functional theory calculations unveiled that multiple molecular-level interactions, such as phenol OH_TA_‧‧‧π_guest_ interactions, occur between the typical TA-treated CuBTC (60 min) and the aromatic compounds. These interactions result in a binding energy of −68.9 kJ mol^−1^, which is markedly stronger than the interaction with cyclic-alkane (−43.8 kJ mol^−1^), despite their closely similar dimensions. Compared with pristine CuBTC (Figs S20 and S21), this amplified discrepancy in binding energy between the two adsorbates substantiates a preferential affinity of the TA-modified framework for aromatic compounds (Figs S22 and S23), indicating that mild TA etching enables precise modulation of the pore surface chemistry.

We measured single-component vapor permeation behavior of five representative components in light naphtha, such as hexane (HEX), 2-methylpentane (2MP), 2,3-dimethylbutane (23DMB), cyclohexane (CH), and benzene (BEN), to evaluate how TA modification tunes membrane performance. These data, interpreted in terms of two membrane structural descriptors (experimental PLD and TAD), demonstrate the potential of TA treatment for tunable petrochemical separations. The triangular pore of the pristine membrane had an experimental PLD of 0.48 nm. This pore size lies between the molecular dimensions of HEX and 2MP, creating a clear size cutoff. Consequently, HEX permeance was measured at 151 liquid permeation units (LPU, where 1 LPU = 10^–9^ mol m^–2^ s^–1^ Pa^–1^), roughly an order of magnitude higher than the permeance of 2MP and the other molecules tested (Fig. 3a).

**Figure 3.**
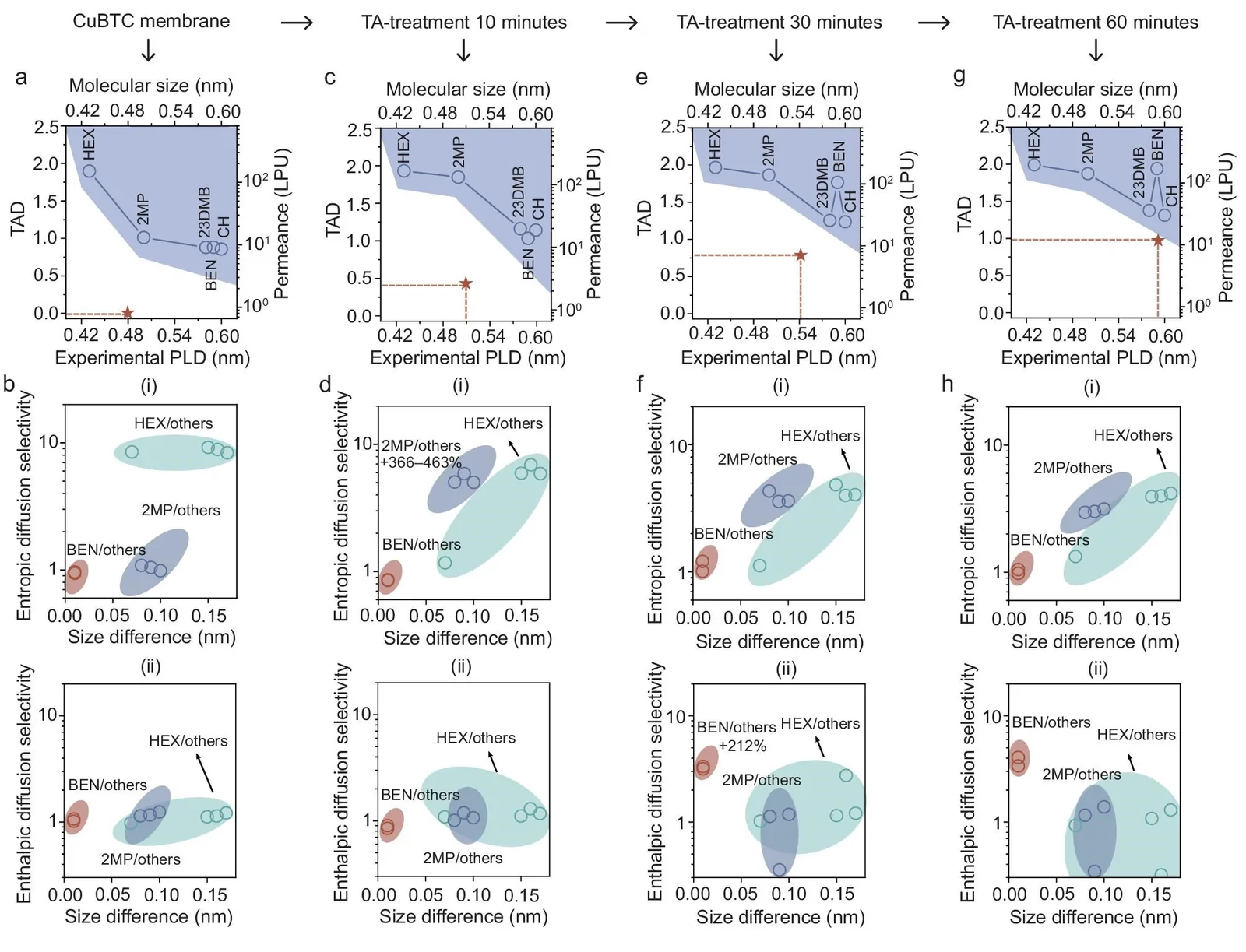
Benchmark permeation and diffusion characteristics of membranes at 333 K for five representative components in light naphtha, as influenced by membrane structural descriptors. (a, c, e, g) Single-component permeation of the pristine CuBTC membrane alongside TA-treated membranes. (b, d, f, h) Diffusion selectivity governed by entropy and enthalpy for various molecular pairs.

Ideal permeation selectivity was defined as the ratio of the permeance of the faster-permeating one to that of the slower one (Tables S7–S22). Accordingly, we deduce that adsorption selectivity and diffusion selectivity varied with TA-treatment duration for petrochemical molecules with distinct sizes (Fig. S24). Furthermore, diffusion selectivity can be factored into an entropic diffusion selectivity, reflecting the membrane’s size discrimination ability [33], and an enthalpic diffusion selectivity, which is governed by pore-vapor interactions [34], as detailed in the Methods. In the pristine CuBTC membrane, the entropic diffusion selectivity of HEX relative to the other molecules is notably high (Fig. 3b), underscoring its ability to discriminate molecular size differences of 0.07–0.17 nm.

After a 10-min TA treatment, the membrane’s PLD increased to between the sizes of 2MP and 23DMB, allowing both HEX and 2MP to permeate (Fig. 3c). This adjustment is practically useful for diversifying ethylene production feedstock. Relative to the pristine membrane, the 10-min TA-treated membrane showed a 366%–463% increase in entropic diffusion selectivity for 2MP over the other molecules (Fig. 3d). This indicates that the treatment substantially improved the membrane’s ability to discriminate among larger molecules with relatively minor differences in molecular size (∼0.08–0.10 nm).

Longer TA treatments further expanded the PLD and increased the membrane’s TAD (Fig. 3e). With these changes, even BEN could permeate through the membrane, despite previously being excluded by the ∼0.54 nm pore size limit. The high density of TA in the pores altered the pore characteristics to enhance BEN transport, effectively overcoming the original sieving cutoff. Notably, the TA modification increased the enthalpic diffusion selectivity favoring BEN by ∼212% compared to the untreated membrane, while the entropic selectivity changed little (Fig. 3f).

With 60 min of TA treatment, the membrane’s PLD nearly matched BEN’s molecular size (Fig. 3g). This alignment, together with a TAD of approximately one TA molecule per MOF slab, allowed the unimpeded permeation of BEN. The permeance of BEN (∼171 LPU) became comparable to that of the smaller HEX and 2MP, and it far exceeded that of 23DMB and CH. This result suggests that the membrane can precisely separate BEN from 23DMB and CH. As expected, the higher TAD mainly boosted the enthalpic selectivity of BEN over these molecules (Fig. 3h), confirming that TA-BEN interactions can enable separation of hydrocarbons whose molecular sizes differ by as small as 0.01 nm.

Notably, we also investigated the influence of other tunable TA treatment parameters, including solution concentration and reaction temperature, on the membrane morphology, structural integrity, TA loading content, and PLD. These results demonstrate that TA-treatment using a 680-ppm TA solution at room temperature delivers the optimal modification performance (Figs S25 and S26).

### Separating dual-component C_6_ mixtures

Building on these findings, we investigated three categories of binary vapor permeation systems to evaluate the separation performance of pristine and TA-treated CuBTC membranes. These systems involved: (1) linear vs. branched alkanes, (2) aliphatic vs. aromatic hydrocarbons, (3) mono-branched vs. di-branched alkanes.

The first category focused on the separation of HEX from 23DMB and BEN. With its small pore size, the pristine CuBTC membrane effectively separated HEX from the larger 23DMB and BEN molecules (Fig. S27a). It achieved an average HEX/23DMB selectivity of 46.9 and a HEX permeance of 119 LPU with a 50/50 HEX/23DMB feed mixture. After TA treatment enlarged the pores, the HEX/23DMB and HEX/BEN selectivities both dropped, while the HEX permeance remained essentially unchanged (Fig. S27b–d).

The second category considered separating 2MP from 23DMB and BEN. The 10-min TA-treated membrane had better size discrimination than the pristine membrane (discussed in Fig. 3d), enabling it to separate 2MP from 23DMB and BEN. Compared to the untreated membrane (Fig. S27e), its 2MP/23DMB and 2MP/BEN selectivities increased by 181% and 266%, respectively (Fig. S27f). However, longer TA treatments reduced these selectivities (Fig. S27g and h), likely because the membrane’s size discrimination became less sharp.

The third category addressed separating BEN from 23DMB and CH, a particularly difficult task given their minimal size difference (0.01 nm). The membrane could not effectively separate BEN from 23DMB or CH until it had undergone at least a 30-min TA treatment (Fig. S27i–k). The 30-min treatment significantly modified the pore functionality by incorporating TA, thereby enhancing interactions with BEN (as discussed in Fig. 3f). Finally, with 60 min of TA treatment, the membrane’s BEN/23DMB and BEN/CH selectivities increased by about 1842% and 1079%, respectively (relative to the pristine membrane), and the BEN permeance rose to 140–144 LPU (Fig. S27l). Notably, prolonged TA treatment for 120 min severely destroyed the crystalline framework and grain boundary structures, giving rise to a marked reduction in separation selectivity (Fig. S28).

Subsequently, the membranes were categorized into four discrete performance tiers—Tier A (optimal), Tier B, Tier C, and Tier D—according to their selectivity values for each binary mixture. This classification provides a selection framework for the complex sorting of light naphtha fractions (Fig. 4a–d).

**Figure 4.**
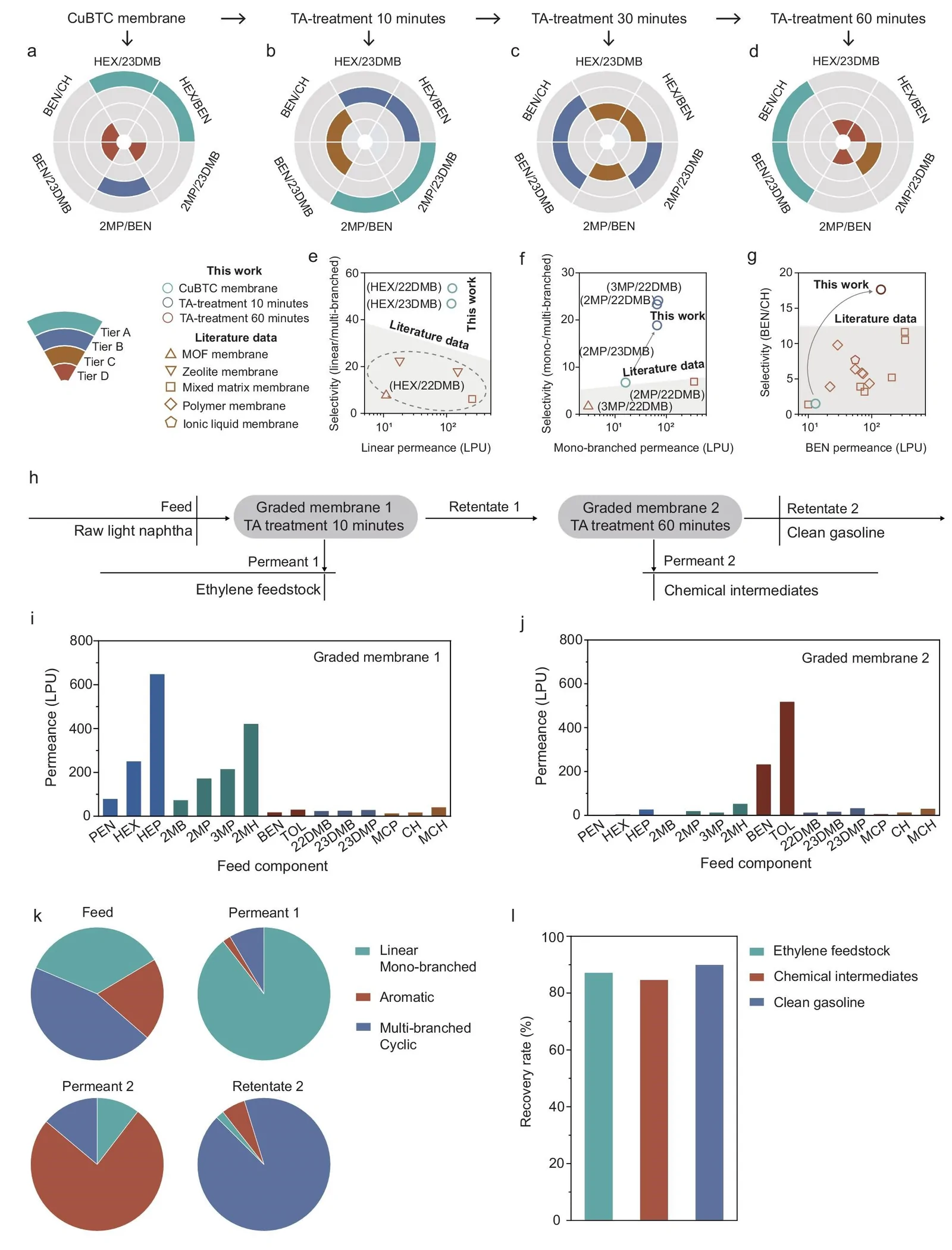
Graded sorting cascade for light naphtha refining at 333 K. (a–d) Ranking membranes according to separation selectivities for binary vapor mixtures. (e–g) Comparison of the current membranes with previously reported membranes for typical dual-component systems. (h) Schematic illustration of the cascade process by employing two TA-treated membranes. (i) Separation data for graded sorting stage 1. Abbreviations used in this figure are as follows: PEN, pentane; 2MB, 2-methylbutane; 3MP, 3-methylpentane; 22DMB, 2,2-dimethylbutane; HEP, heptane; 2MH, 2-methylhexane; 23DMP, 2,3-dimethylpentane; TOL, toluene; MCP, methylcyclopentane; MCH, methylcyclohexane. The feed solution consisted of PEN/HEX/HEP/2MB/2MP/3MP/2MH/22DMB/23DMB/23DMP (each 5 wt.%) and BEN/TOL/MCP/CH/MCH (each 10 wt.%). (j) Separation data for graded sorting stage 2. (k) Compositions of the raw feed and products from each separation stage. (l) Recovery rates of the ethylene feedstock, chemical intermediates, and clean gasoline.

In comparison, the pristine CuBTC membrane provided exceptional separation for HEX/23DMB and HEX/22DMB mixtures, outperforming previously reported membranes (Fig. 4e). After a 10-min TA treatment and a 60-min TA treatment, the CuBTC membranes also showed greatly improved separation for the mono-branched/multi-branched alkanes and BEN/CH mixtures, respectively. In fact, they surpassed a range of membranes documented in the literature, including other MOF membranes, zeolite membranes, mixed matrix membranes, polymer membranes, and ionic liquid membranes (Fig. 4f and g; Tables S23–S25).

In addition, the reproducibility of the TA-treatment strategy was confirmed by evaluating separation performance of ten individually fabricated membranes in a standard vapor permeation setup. Around 70% of these membranes exhibited the target separation performance (Fig. S29). A long-duration vapor permeation experiment (Fig. S30), combined with the nearly unchanged TA loading content measured pre- and post-testing (Fig. S31), corroborated the robust immobilization of TA within the membrane matrix. The fabrication of CuBTC membranes and subsequent TA-treatment can be scaled up on industrially relevant tubular α-Al_2_O_3_ supports (Fig. S32). These results collectively demonstrate the promising practical utility of separation membranes developed in our study.

The gentle etching strategy relying on natural polyphenols in the current study possesses broad universality. Gallic acid and ellagic acid deliver analogous pore-modulation effects on CuBTC membranes (Figs S33–S35). The TA-etching method can also be readily extended to other MOF membranes including Zn-BODC and Al-BODC without compromising framework integrity (Figs S36 and S37). These findings suggest a general route for programming pore size and functionality across diverse MOF architectures.

### Graded sorting cascade for light naphtha refining

We developed a graded sorting cascade using two TA-treated membranes (one treated for 10 min and another for 60 min). The goal was to fractionate a simulated light naphtha mixture (15 components, linear/mono-branched/aromatic/multi-branched/cyclic = 15/20/20/15/30 by weight) into three distinct product streams (Fig. 4h): linear and mono-branched alkanes for ethylene feedstock, aromatic compounds for chemical intermediates, and multi-branched and cyclic alkanes for clean gasoline. As expected, the cascade successfully extracted the linear/mono-branched alkanes and the aromatic compounds from the complex mixture (Fig. 4i and j), thereby enriching the desired components in each product (Fig. 4k). This graded sorting cascade achieved about 85%–90% recovery for each of the three fractions (ethylene feedstock, chemical intermediates, and clean gasoline) (Fig. 4l). Raising the cascade vapor permeation temperature from 333 K to 353 K yielded negligible fluctuations in product purity and recovery ratio, which manifests the outstanding industrial compatibility of this separation platform (Fig. S38).

To assess the energy efficiency of this process, we performed chemical process modeling on a representative five-component mixture (HEX/2MP/BEN/22DMB/CH = 15/20/20/15/30 by weight). The membrane cascade consumed an estimated 0.39 GJ per ton of feed to reach 90% recovery of all three target product streams, a 91% reduction in energy use compared to conventional distillation (Figs S39 and S40). Techono-economic analysis at the full process scale reveals that the membrane cascade delivers drastically lower annual operational expenses in comparison with conventional distillation (Tables S26 and S27).

## CONCLUSION

Molecular-level separation of light naphtha is challenging because conventional membranes have limited selectivity, especially for components with very similar physicochemical properties. In this study, we demonstrated a graded sorting cascade that leverages differences in molecular size and aromaticity to fractionate complex naphtha in an energy-efficient manner. This system uses a series of membranes whose pore sizes and surface chemistries are continuously tuned via mild TA etching. Incorporating these membranes in sequence allowed us to divide a fifteen-component feed into three high-purity product streams, each recovered at 85%–90%. These streams correspond to ethylene production feedstock enriched in linear and mono-branched alkanes, chemical intermediates primarily containing aromatics, and clean gasoline predominantly composed of multi-branched and cyclic alkanes. Notably, this membrane-based strategy reduced energy consumption by 91% compared to distillation. The proposed graded sorting paradigm thus offers a novel platform for petrochemical refining, maximizing value extraction while greatly improving energy efficiency.

## METHODS

### Synthesis of CuBTC membranes

Metal ion and linker solutions were prepared by dissolving 0.218 g of Cu(NO_3_)_2_·3H_2_O and 0.126 g of 1,3,5-benzenetricarboxylic acid in 10 ml of DMF, respectively. The metal ion solution was slowly added dropwise into the linker solution to form a clear precursor solution. Then the support was placed horizontally in the precursor solution, followed by ultrasonication for 2 min and keeping static at 25°C for 60 min to fulfil the static seeding process. After that, the alumina support was removed from the precursor solution and thermal conversion was implemented at 150°C for 120 min. After an SS-TC cycle, the membranes were thoroughly washed with DMF and then dried at 120°C for 12 h under a vacuum condition.

### Mild etching of CuBTC membranes by tannic acid

An etching solution was prepared by dissolving 0.034 g of TA in 50 ml of deionized water to obtain a concentration of 0.68 g L^−^^1^ (680 ppm). The CuBTC membrane was horizontally immersed in the solution and stirred at 200 rpm at room temperature for 10–60 min. Upon completion, the membrane was thoroughly washed with deionized water to remove residual TA.

## Supplementary Material

nwag488_Supplemental_File
