# Supplementary material for: Molecular-level petroleum refining by graded membranes
Source: Natl Sci Rev. 2026 Aug 12;13(18):nwag488. doi: 10.1093/nsr/nwag488 (PMC13622342; doi:10.1093/nsr/nwag488)
Supplement: Supplementary file 1 [file nwag488_supplemental_file.pdf]

Supplementary Materials for

Molecular-Level Petroleum Refining by Graded Membranes

Yuecheng Wang[1,2,†], Fang-Di Dong[3,†], Yujie Ban[1,2,*], Ziyi Hu[1], Dong-Dong Zhou[3,*], Jie-Peng Zhang[3], Xiao-Ming Chen[3], Weishen Yang[1,2,*]

[1] State Key Laboratory of Catalysis, Dalian Institute of Chemical Physics, Chinese Academy of Sciences, 457 Zhongshan Road, Dalian 116023, P. R. China.

[2] University of Chinese Academy of Sciences, 19A Yuquan Road, Beijing 100049, P. R. China.

[3] MOE Key Laboratory of Bioinorganic and Synthetic Chemistry, GBRCE for Functional Molecular Engineering, School of Chemistry, IGCME, Sun Yat-Sen University, Guangzhou 510275, P. R. China.

***Corresponding authors**. E-mails: yjban@dicp.ac.cn; yangws@dicp.ac.cn; zhoudd3@mail.sysu.edu.cn

†Equally contributed to this work.

This PDF file includes:

Materials and Methods
Figs. S1 to S40
Tables S1 to S27
References (1 to 22)

Materials and Methods

Chemicals.

Copper(II) nitrate trihydrate ($Cu(NO_3)_2 \cdot 3H_2O$, ≥98%), aluminum chloride hexahydrate ($AlCl_3 \cdot 6H_2O$, ≥98%), bicyclo[2.2.2]octane-1,4-dicarboxylic acid (BODCA, $C_{10}H_{14}O_4$, 98%) and 1,3,5-benzenetricarboxylic acid (BTCA, $C_9H_6O_6$, 99%) were purchased from Sigma-Aldrich Inc. Aqueous HF solution (40.0 wt.%), tannic acid ($C_{76}H_{52}O_{46}$, 99%), gallic acid ($C_7H_6O_5$, 99%), ellagic acid ($C_{14}H_6O_8$, 99%), zinc nitrate hexahydrate ($Zn(NO_3)_2 \cdot 6H_2O$, ≥99.5%) and urea (CH_4N_2O, ≥99.5%) were purchased from Shanghai Aladdin Biochemical Technology Co., Ltd. N,N'-dimethylformamide (DMF, C_3H_7NO, ≥99.5%), methanol (CH_4O, ≥99.5%), ethanol (C_2H_6O, ≥99.5%), *n-/i*-propanol (C_3H_8O, ≥99.5%), *n-/i*-butanol ($C_4H_{10}O$, ≥99.5%), pyridine (C_5H_5N, ≥99.5%), ethyl acetate ($C_4H_8O_2$, ≥99.5%), acetonitrile (C_2H_3N, ≥99.5%), sodium hydroxide (NaOH, ≥99 %), formic acid, and acetic acid were obtained from Sinopharm Chemical Reagent Co., Ltd. The α-Al_2O_3 disks (diameter: 18 mm, pore size: 70 nm) and the tubular α- Al_2O_3 supports (inner diameter: 7.2 mm, length: 85 mm) used in this work were both prepared in our laboratory.

Mild etching of CuBTC membranes by gallic acid and ellagic acid.

An etching solution was prepared by dissolving 0.034 g of gallic acid (or ellagic acid) in 50 mL of deionized water to obtain a concentration of 0.68 g L^{-1} (680 ppm). The CuBTC membrane was horizontally immersed in the solution and stirred at 200 rpm at room temperature for 10–60 min. Upon completion, the membrane was thoroughly washed with deionized water to remove residual acid.

Fabrication of Zn-BODC membrane.

The Zn-Al LDH priming layer was synthesized prior to the fabrication of Al-BODC membrane. A precursor solution was prepared by dissolving 0.20 g of $Zn(NO_3)_2 \cdot 6H_2O$ and 0.10 g urea in 25 mL of deionized water. Then the porous γ-Al_2O_3 substrate was horizontally placed and sealed in a 50 ml Teflon-lined stainless steel autoclave containing the precursor solution. The autoclave was put into an oven with the temperature pre-heated to 75°C for 24 h, and then cooled naturally to room temperature. The membrane disk was thoroughly washed with deionized water and then dried at 60°C for 12 h in a vacuum condition.

A precursor solution was prepared by dissolving 0.011 g $Zn(NO_3)_2 \cdot 6H_2O$ and 0.05 g bicyclo[2.2.2]octane-1,4-dicarboxylic acid in 25 mL of DMF. An γ-Al_2O_3 disk pre-coated with the LDH priming layer was placed horizontally in the precursor solution and sealed in a Teflon-lined autoclave. The reaction was carried out at 120°C for 24 h. After natural cooling, the membrane was washed three times with DMF and methanol, and then soaked overnight in methanol to remove residual ligands and solvents. Finally, the sample was thermally activated by vacuum drying at 100°C for 24 h.

Fabrication of Al-BODC membrane.

A precursor solution was prepared by dissolving 0.012 g $AlCl_3 \cdot 6H_2O$, 0.05 g bicyclo[2.2.2]octane-1,4-dicarboxylic acid, and 0.01 g NaOH in 25 mL of deionized water. An γ-Al_2O_3 disk pre-coated with the LDH priming layer was placed horizontally in the precursor solution and sealed in a Teflon-lined autoclave. The reaction was carried

out at 200°C for 24 h. After natural cooling, the membrane was washed three times with DMF and methanol, and then soaked overnight in methanol to remove residual ligands and solvents. Finally, the sample was thermally activated by vacuum drying at 100°C for 24 h.

Mild etching of Zn-BODC and Al-BODC membranes by tannic acid.
An etching solution was prepared by dissolving 0.034 g of TA in 50 mL of deionized water to obtain a concentration of 0.68 g L^{-1} (680 ppm). The Zn-BODC and Al-BODC membrane was horizontally immersed in the solution and stirred at 200 rpm at room temperature for 10–60 min. Upon completion, the membrane was thoroughly washed with deionized water to remove residual TA.

Characterizations.
PXRD patterns of the samples were recorded on a Rigaku instrument (Smartlab) using Cu Kα radiation (λ = 0.154 nm at 40 kV and 200 mA). The scan speed was 10° min^{-1}, and the 2θ range was 5–50°. The top-view and cross-sectional morphology of the membranes was probed by an SEM (Quanta 200 FEG, FEI Co.) with an acceleration voltage of 20 kV. The alumina-supported membrane was broken and pasted on a home-made metal holder. Sputter coating of conductive gold nanoparticles on the sample was needed to prevent charge accumulation. Membranes were digested with DCl solution (35 wt.% in D_2O) or HCl solution (36 wt.% in H_2O) before performing liquid-state ^{1}H NMR (Bruker AVANCE III 400 MHz) and ICP-AES measurements (Shimadzu ICPS-8100), respectively. Raman spectra were collected on a Bruker Optics Senterra Raman spectrometer using a laser with a wavelength of 532 nm. FTIR spectra in the mode of attenuated total reflection were obtained on a Thermo-Fisher spectrometer (Nicolet is50) from 400 to 4000 cm^{-1}. XPS was performed on a quasi-in situ X-ray photoelectron spectrometer (Thermo Fisher Escalab 250 Xi+; Thermo Fisher Scientific).

Single-component gas permeation.
The single-component gas permeations of H_2, CH_4, C_2H_6, C_3H_8 and n-C_4H_{10} were conducted at 298 K with a constant feed flow rate of 100 ml min^{-1}. The permeate side was continuously swept by 100 ml min^{-1} of Ar. A gas chromatograph (Agilent 7890B) equipped with a capillary column (HP-PLOT Al_2O_3 KCl, Agilent) and a highly sensitive flame ionization detector was used for online monitoring of the concentration of permeants.

Analysis of the membrane's experimental PLD.
The NKP method established on the gas translation model has been proposed for determining the membrane's experimental PLD values below 1 nm [1–3]. Under the assumption of the Knudsen diffusion behavior, the ratio of the permeance of a given component i to that of a reference component (typically H_2) corresponds to the inverse ratio of the molar mass of the component i to that of H_2. Therefore, the predicted Knudsen permeance of the component i ($P_{i\text{-}K}$) can be calculated using the following equation:

$$P_{\text{i-K}} = P_{\text{H}_2}\sqrt{M_{\text{H}_2}/M_{\text{i}}} \tag{1}$$

The NKP coefficient (β) is defined as the ratio of the experimentally obtained permeance (P_{i}) of the component i to its predicted Knudsen permeance, as shown in the following equation:

$$\beta = \frac{P_{\text{i}}}{P_{\text{H}_2}\sqrt{M_{\text{H}_2}/M_{\text{i}}}} \tag{2}$$

According to the gas translation model, the permeance of the component i through the microporous membrane (assuming cylindrical pores) can be formulated with the following equation:

$$P_{\text{i}} = \frac{(d_{\text{p}} - d_{\text{i}})}{3}\left(\frac{(d_{\text{p}} - d_{\text{i}})^2}{d_{\text{p}}^2}\right)\sqrt{\frac{8}{\pi M_{\text{i}} RT}} \cdot \frac{\varepsilon_{\text{i}}}{\tau_{\text{i}} L_{\text{i}}} \cdot \exp\left(-\frac{E_{\text{p,i}}}{RT}\right) \tag{3}$$

The parameters d_{p}, ε and τ denote the size, porosity and tortuosity of the membrane's pores, respectively, while L is the membrane thickness. The variables d_{i}, M_{i}, and E_{i} correspond to the molecular size (kinetic diameter), molecular mass, and permeation activation energy of the component i, respectively. The term d_{p}-d_{i} corresponds to the diffusion distance of the component in the pore. The universal gas constant and temperature are represented by R and T. Therefore, by inserting Eq. (3) into Eq. (2), β can be expressed as follows, assuming the activation energies, $E_{\text{P,i}}$, are the same for any type of gas.

$$\beta \approx \frac{(d_{\text{p}} - d_{\text{i}})^3}{(d_{\text{p}} - d_{\text{k,H}_2})^3} \tag{4}$$

$$\beta \approx \frac{(d_{\text{p}} - d_{\text{i}})^3}{(d_{\text{p}} - 0.29)^3} \tag{5}$$

The pore sizes, d_p, particularly reflecting the PLD of microporous membranes was derived by fitting Eq. (5).

Vapor permeation experiments.
Vapor permeation through the membrane was conducted at 333 K. The membrane was sealed into a home-designed dead-end stainless-steel module, with an effective membrane area of 0.9499 cm^2. Generally, the front side of the membrane was positioned approximately 1 cm above the feed liquid (50 ml), and the back side was evacuated by an oil pump (Edwards RV12) to maintain a vacuum. When the permeation reached a steady state, the permeant was collected in a liquid nitrogen cold trap with an interval of 1 to 6 h. The composition of the permeant was analyzed by a gas chromatography (GC, Agilent 7890B). The permeation flux (J) was calculated via:

$$J = \frac{W}{A \times t} \quad (6)$$

where W is the amount of permeant (kg), A is the effective membrane area (m^2), and t is the permeation time (h). The permeance of each component P_i (mol m^{-2} s^{-1} Pa^{-1}) was obtained via:

$$P_i = \frac{J}{3600 \times (p_f - p_p) \times M_r} \quad (7)$$

where p_f (kPa) and p_p (kPa) are the vapor pressures of component i on the feed and permeate sides, respectively. The p_f is calculated according to the Antoine equation and Raoult's law. The p_p is considered to be zero in the vacuum mode of vapor permeation. M_r (g mol^{-1}) stands for the molar weight of component i. Permeance was reported in liquid permeation units (LPU), where 1 LPU = 10^{-9} mol m^{-2} s^{-1} Pa^{-1}. LPU was used because the permeating species were condensable hydrocarbon vapours rather than permanent gases, making it a more appropriate metric for vapour permeation and pervaporation than the commonly used gas permeation unit (GPU). Accordingly, the selectivity (α) of component i over component j was determined by their permeance ratio:

$$\alpha = \frac{P_i}{P_j} \quad (8)$$

Single-component vapor adsorption.

The single-component alkane adsorption capacity of the membrane was measured based on the weighing method. A volume of 5 mL of a pure alkane was placed in a 50 mL beaker to serve as the vapor source. The MOF membrane was suspended above the liquid phase using a holder, ensuring that adsorption occurred exclusively from the vapor phase without direct liquid contact. The beaker was then sealed with plastic film to establish a closed adsorption environment and placed in an oven at either 303 K or 333 K for 2 h to allow adsorption to reach equilibrium. After adsorption, the beaker was opened and the membrane was exposed to ambient conditions for 2 min to allow the desorption of weakly bound vapor. The membrane was weighed before and after adsorption, and the mass difference was used to determine the single-component adsorption capacity.

Calculation of diffusion entropic and enthalpic selectivity.

The membrane selectivity can be rewritten as diffusion selectivity product adsorption selectivity:

$$\alpha = \frac{P_\mathrm{i}}{P_\mathrm{j}} = \left(\frac{D_\mathrm{i}}{D_\mathrm{j}}\right) \times \left(\frac{S_\mathrm{i}}{S_\mathrm{j}}\right) \quad (9)$$

Based on the weighing method, we can calculate the adsorption selectivity of component i to component j by the ratio of their adsorption amounts, and further obtain diffusion selectivity according to Eq. (9). Diffusion selectivity is determined by entropic selectivity and enthalpic selectivity, namely:

$$\frac{D_{\mathrm{i}}}{D_{\mathrm{j}}}=\left[\exp\left(\frac{S_{\mathrm{D,i}}-S_{\mathrm{D,j}}}{R}\right)\right]\left[\exp\left(-\frac{E_{\mathrm{D,i}}-E_{\mathrm{D,j}}}{RT}\right)\right] \quad (10)$$

$$\text{Entropic selectivity}=\exp\left(\frac{S_{\mathrm{D,i}}-S_{\mathrm{D,j}}}{R}\right) \quad (11)$$

$$\text{Enthalpic selectivity}=\exp\left(-\frac{E_{\mathrm{D,i}}-E_{\mathrm{D,j}}}{RT}\right) \quad (12)$$

where R is the universal gas constant, T is the absolute temperature, $E_{\mathrm{D,i}}$ and $E_{\mathrm{D,j}}$ are the diffusion activation enthalpies, and $S_{\mathrm{D,i}}$ and $S_{\mathrm{D,j}}$ are the diffusion activation entropies, respectively. In logarithmic form:

$$\ln\frac{D_{\mathrm{i}}}{D_{\mathrm{j}}}=\left(-\frac{\Delta E_{\mathrm{D_{i/j}}}}{R}\right)\frac{1}{T}+\left(\frac{\Delta S_{\mathrm{D_{i/j}}}}{R}\right) \quad (13)$$

Through temperature-variable diffusion selectivity, we can obtain diffusion enthalpic selectivity and diffusion entropic selectivity.

Structural simulation of CuBTC following TA treatment.

Computational simulations were performed utilizing the Materials Studio 5.5 software package. The MOF slab was constructed based on the (111) crystallographic plane of pristine CuBTC. Structural geometry optimization was carried out by employing the Dreiding force field within the Forcite module. Partial charges for both the MOF framework and the TA molecule were calculated by the charge equilibration method. The convergence criteria were established as follows: energy tolerance of 2×10^{-5} kcal mol^{-1}, force tolerance of 1.0×10^{-3} kcal mol^{-1} Å^{-1}, and displacement tolerance of 1.0×10^{-5} Å. Guest adsorption was investigated using grand canonical Monte Carlo (GCMC) simulations with a fixed loading task at 298 K, implemented within the Sorption module. The GCMC simulations employed the Metropolis algorithm in conjunction with the Dreiding force field. Atomic charges were assigned based on Mulliken charges derived from density functional theory (DFT) calculations. A cutoff radius of 18.5 Å was applied for the Lennard-Jones potential. Electrostatic and van der Waals interactions were treated using the Ewald summation and atom-based summation methods, respectively. Both equilibration and production phases consisted of 10^7 steps.

DFT calculations were conducted using the Dmol3 module. Geometry optimizations utilized the generalised gradient approximation (GGA) with the Perdew-Burke-Ernzerhof (PBE) functional, double numerical plus d-fuctions (DNP) basis set, Grimme's DFT-D correction, and effective core potentials (ECP). Convergence criteria for energy, gradient, and displacement were set to 1×10^{-5} Ha, 2×10^{-3} Å and 5×10^{-3} Å, respectively. Both the host framework and guest molecules were treated as flexible entities. Binding energies (ΔE) were calculated according to:

$$\Delta E = E_{host+guest} - E_{host} - E_{guest} \quad (14)$$

where E_{host}, E_{guest}, and $E_{host+guest}$ represent the total energies of the host, guest, and host-guest complex, respectively.

Chemical process simulation.

Chemical process simulations were conducted to separate a five-component model mixture using a graded membrane cascade and vacuum distillation, respectively. The simulation was performed via Aspen Plus v7.2 software. The model mixture consisted of HEX, 2MP, BEN, 23DMB and CH in a weight ratio of 15: 20: 20: 15: 30, respectively. This composition was selected to representatively correspond to the overall proportions of linear alkanes, mono-branched alkanes, aromatics, multi-branched alkanes, and cyclic alkanes observed in a 15-component separation study.

Table S1. Typical structures and nomenclature of light naphtha components ranging from C_5 to C_7.

Name	Structure	Abbreviation	Molecular size (nm)
Pentane	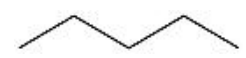	PEN	0.45
2-Methylbutane	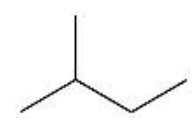	2MB	0.50
Methylcyclopentane	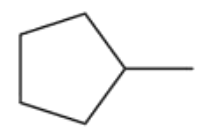	MCP	0.60
Hexane	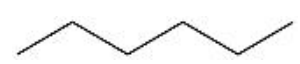	HEX	0.43
2-Methylpentane	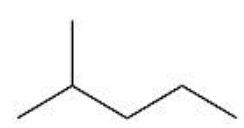	2MP	0.50
3-Methylpentane	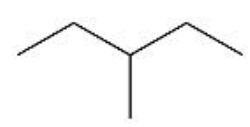	3MP	0.50
2,2-Dimethylbutane	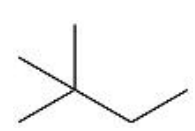	22DMB	0.62
2,3-Dimethylbutane	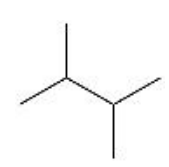	23DMB	0.58
Cyclohexane	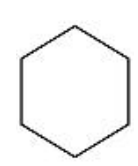	CH	0.60
Benzene	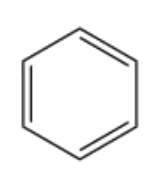	BEN	0.59
Heptane	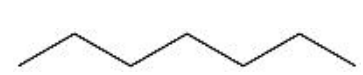	HEP	0.43
2-Methylhexane	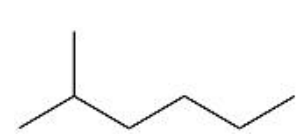	2MH	/
2,3-Dimethylpentane	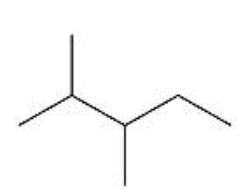	23DMP	/
Toluene	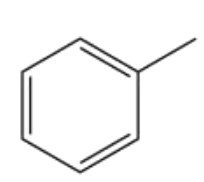	TOL	/
Methylcyclohexane	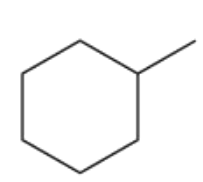	MCH	/

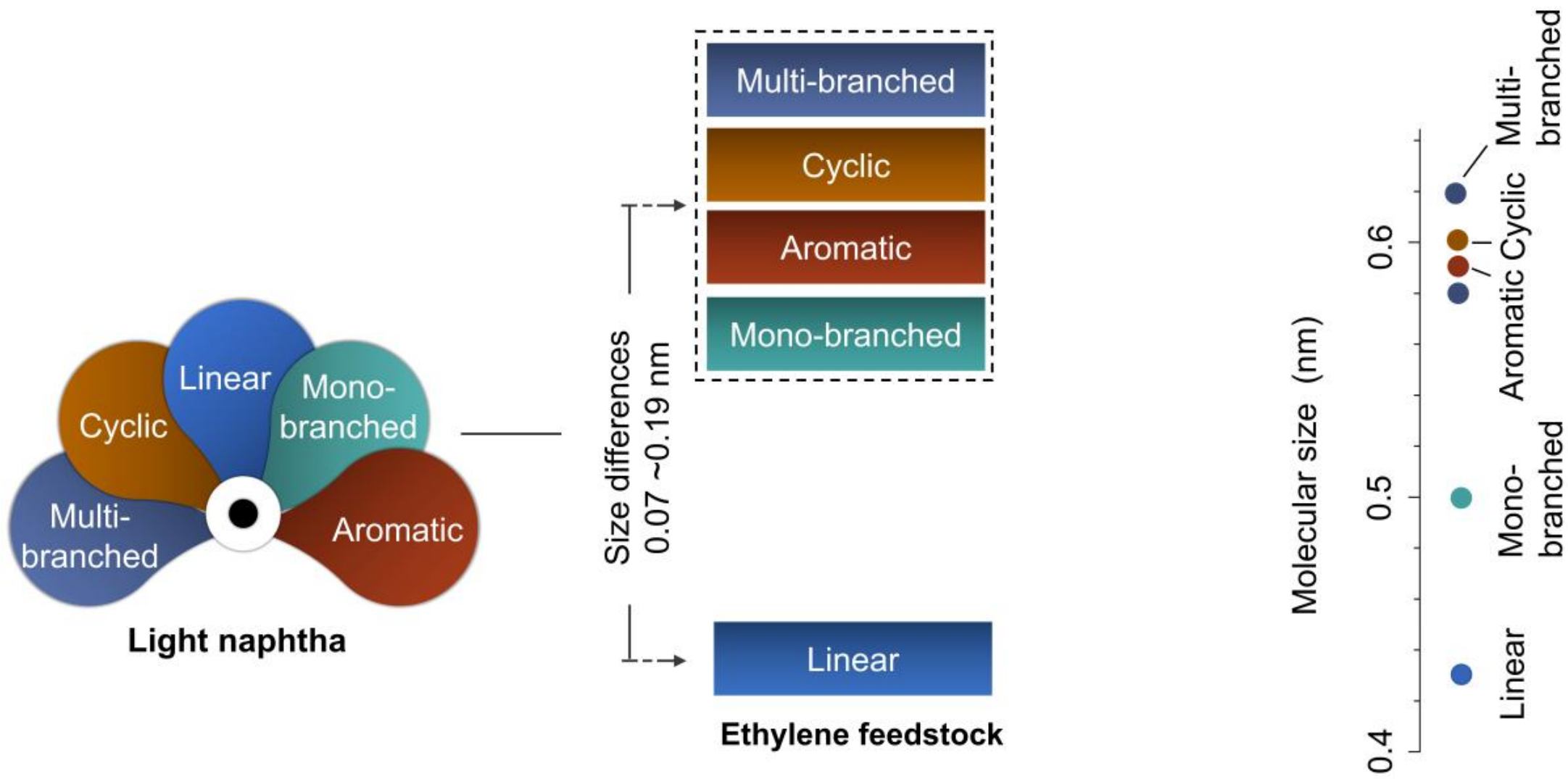

Fig. S1. Schematic illustration of traditional sieving of light naphtha, using the C_6 component as an example.

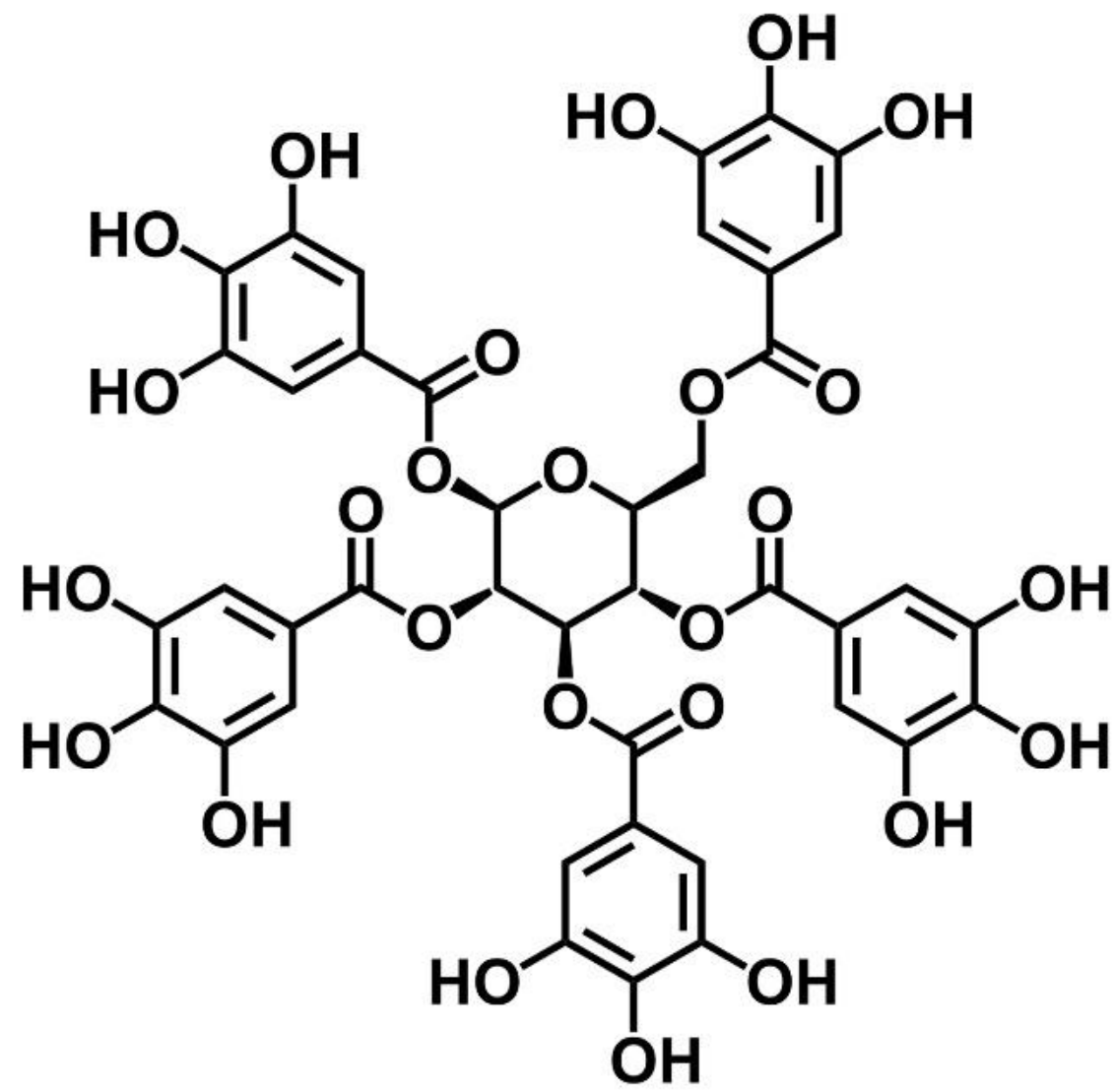

Fig. S2. Chemical structure of TA.

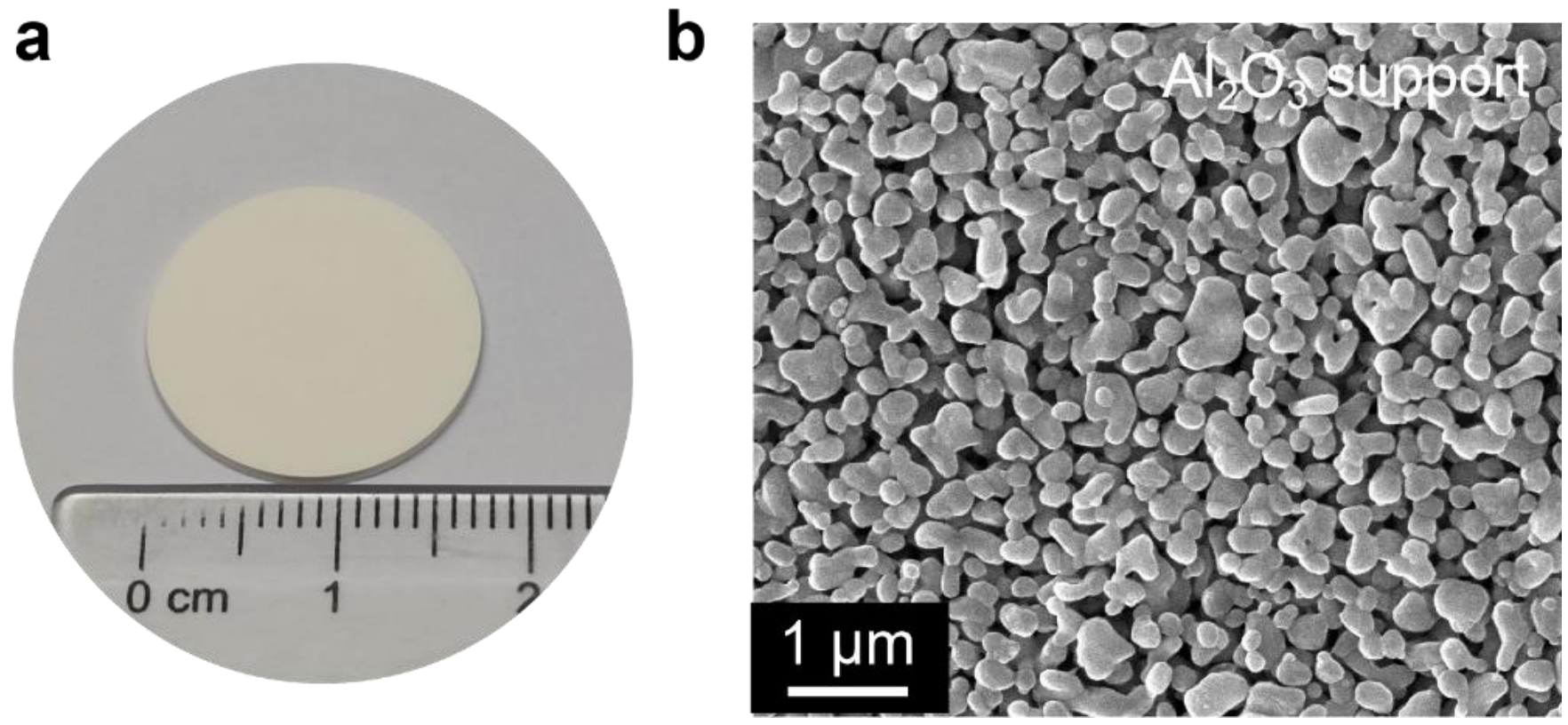

Fig. S3. Morphological characterization of the bare α-Al_2O_3 support disk. (a) Digital photograph. (b) Top-view SEM image.

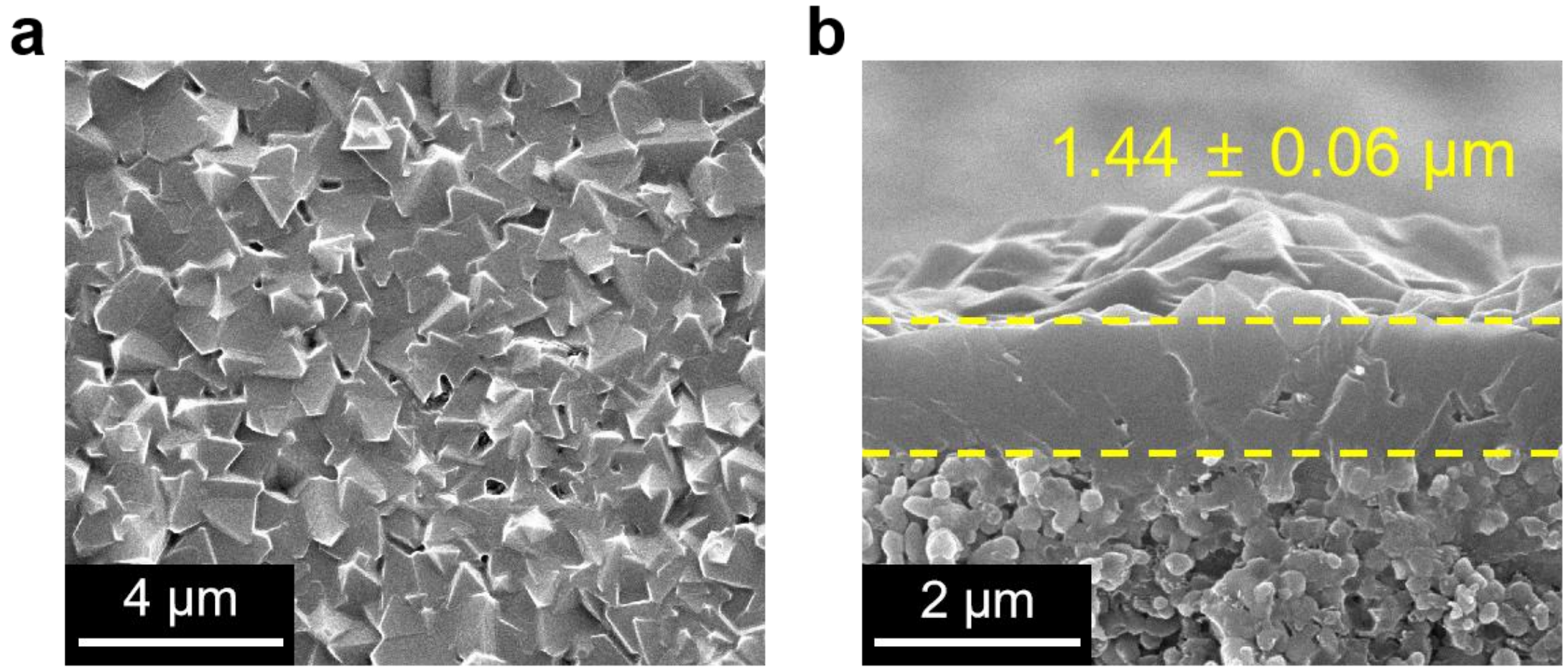

Fig. S4. SEM images of the CuBTC membrane synthesized using the SS-TC method. (a) Top-view SEM image. (b) Cross-sectional SEM image.

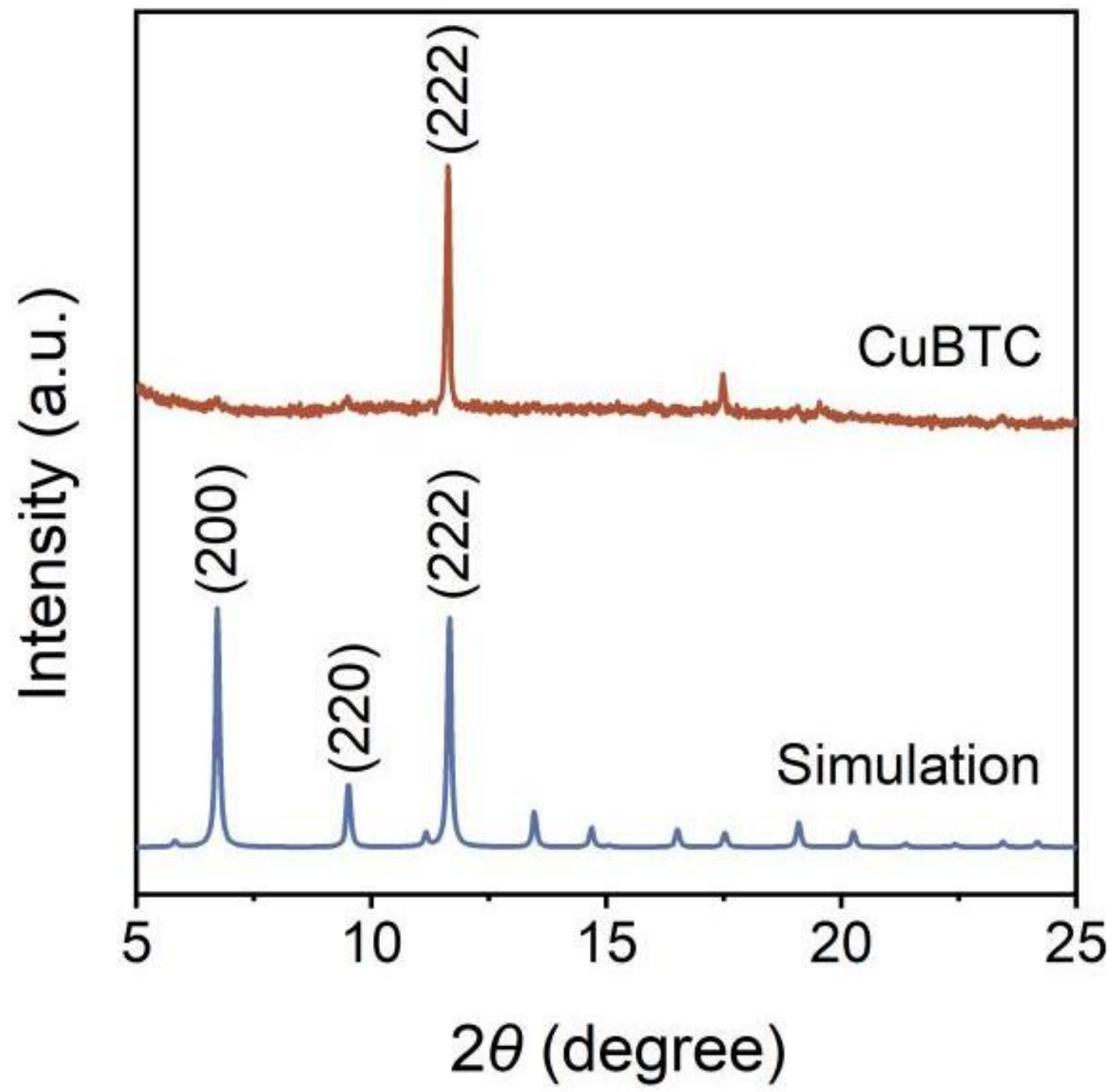

Fig. S5. The XRD pattern of the CuBTC membrane synthesized via the SS-TC method.

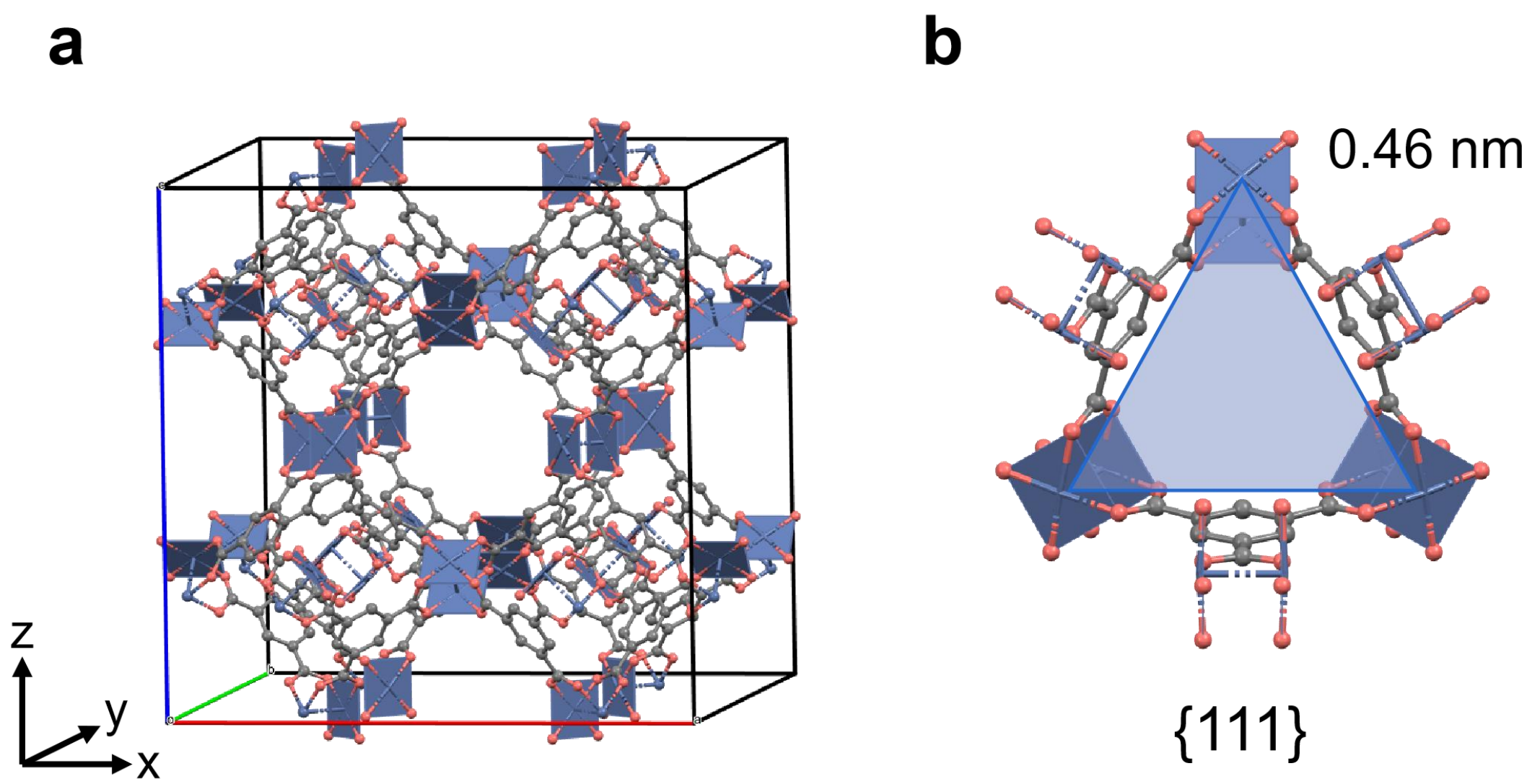

Fig. S6. Structures of CuBTC. (a) Cubic unit cell. (b) Triangular sieving pore along the {111} crystal plane.

The structure of CuBTC is depicted using ball-and-stick models, where Cu are represented in dark blue, C in dark gray and O in red. As shown in Fig. S6b, the {111} crystal plane reveals an alignment of octahedral cages, which exposes triangular sieving pores measuring 0.46 nm in size, indicated by a light blue triangle.

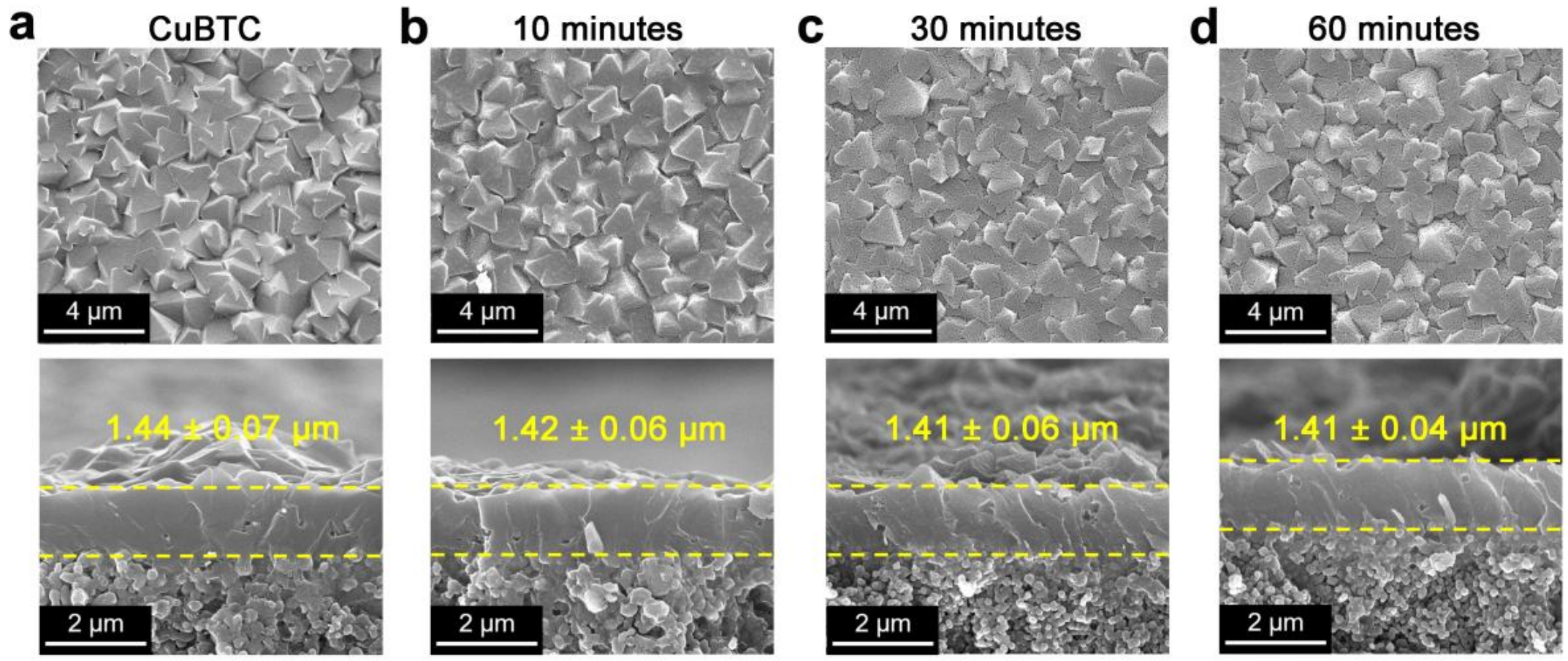

Fig. S7. SEM images including top-view and cross-sectional perspectives of CuBTC membranes subject to TA treatment for durations ranging from 0 to 60 minutes.

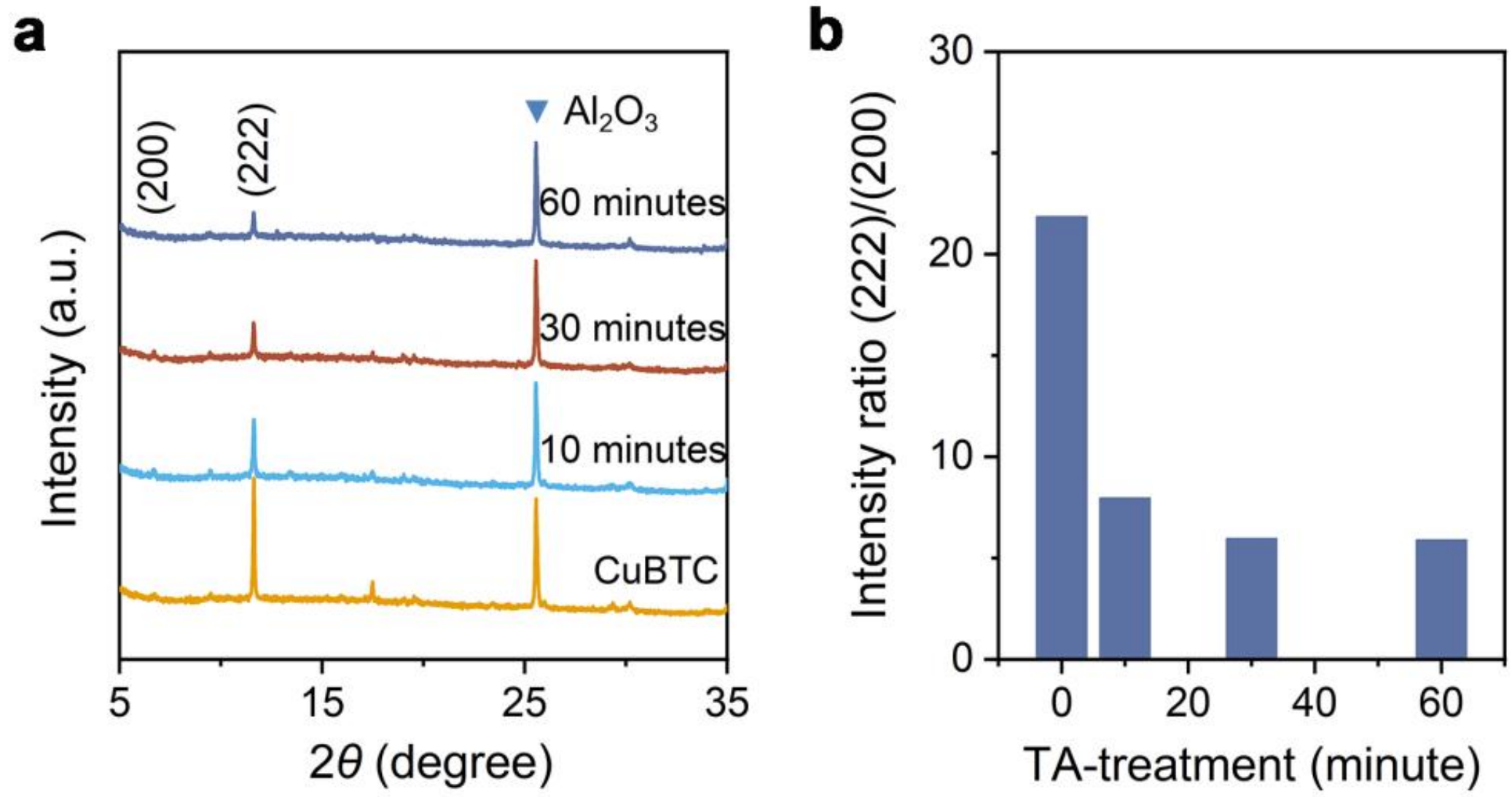

Fig. S8. Orientation of the pristine and TA-treated CuBTC membranes. (a) XRD patterns. (b) The intensity ratio (222) to (200) diffractions of the membranes determined from the experimental patterns.

The diffraction peak of the Al_2O_3 substrate at $2\theta = 25.5^o$ was used as a reference to normalize the (200) and (222) peaks of the membrane. The crystallinity of the {111} crystal planes was quantified by calculating the intensity ratio of the (222) to (200) diffractions. The results indicate that TA treatment led to a marked reduction in the (222) peak intensity, suggesting a preferential effect on the highly exposed {111} planes.

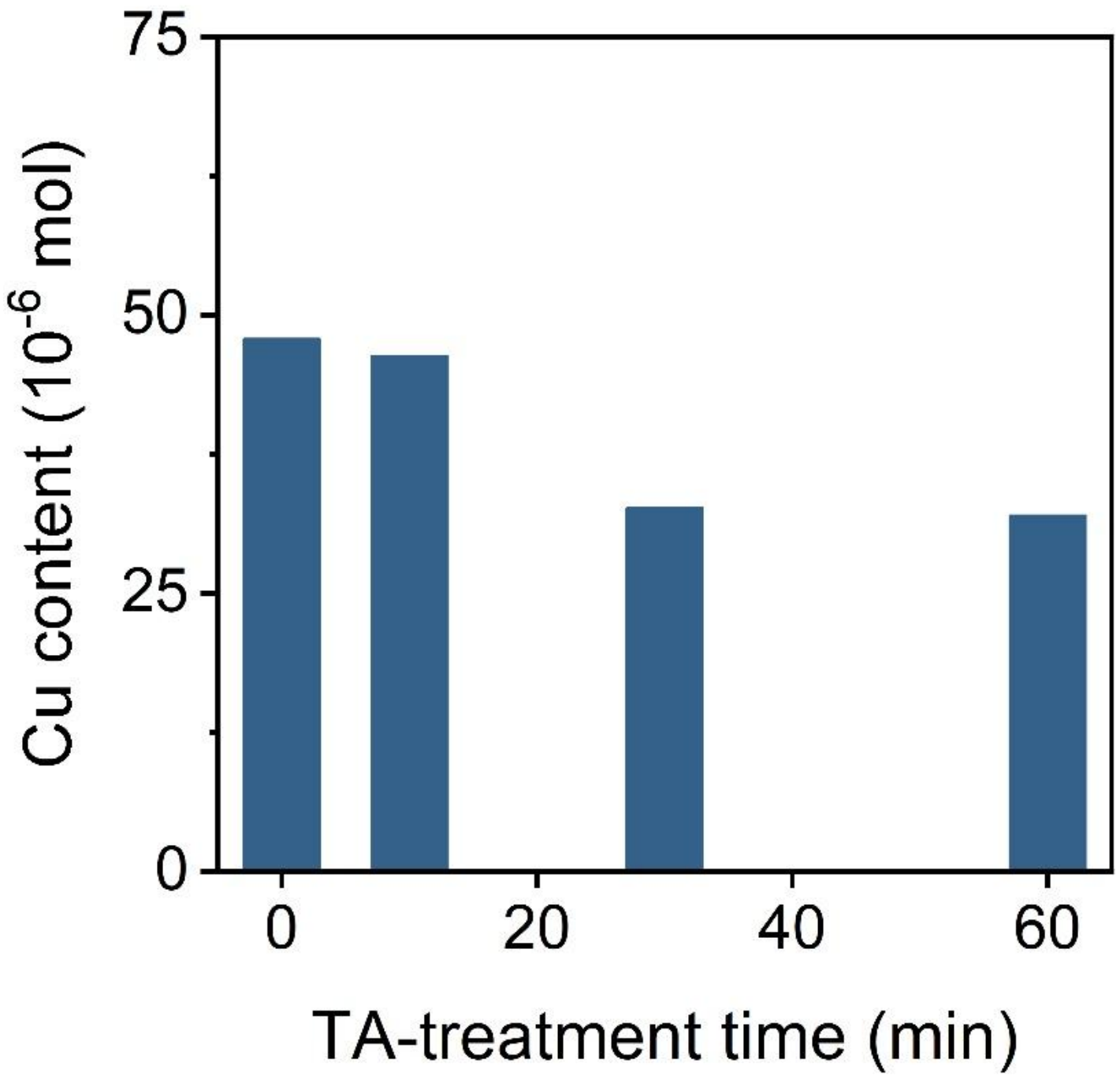

Fig. S9. Determination of Cu content in the CuBTC membrane and TA-treated membranes.

The membranes were immersed in 25 mL of 20 wt.% aqueous hydrochloric acid at 60 °C for 1 h to ensure complete degradation of the MOF layer. Subsequently, the Cu content in the membrane was quantified using ICP-AES analysis.

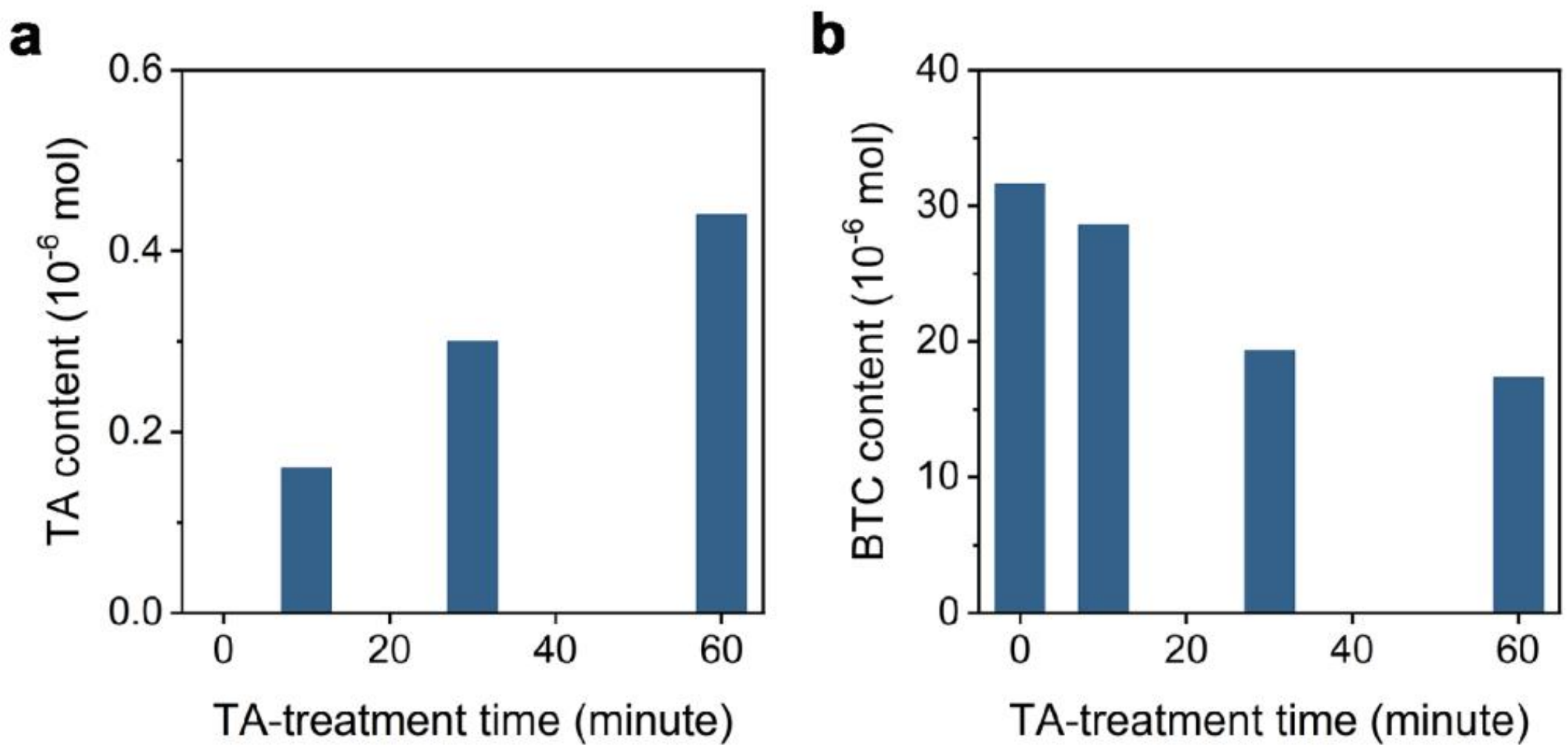

Fig. S10. Determination of organic content in the CuBTC membrane and TA-treated membranes. (a) TA. (b) BTC.

The membranes were treated with 0.5 mL of deuterated hydrochloric acid at 60 °C for 1 h to ensure complete decomposition of the MOF layer. Subsequently, the liquid was diluted with 1 mL of deuterated DMSO. The amounts of BTC and TA present within the membranes were determined using ^{1}H NMR spectroscopy, employing 5 mg of terephthalic acid as an internal standard.

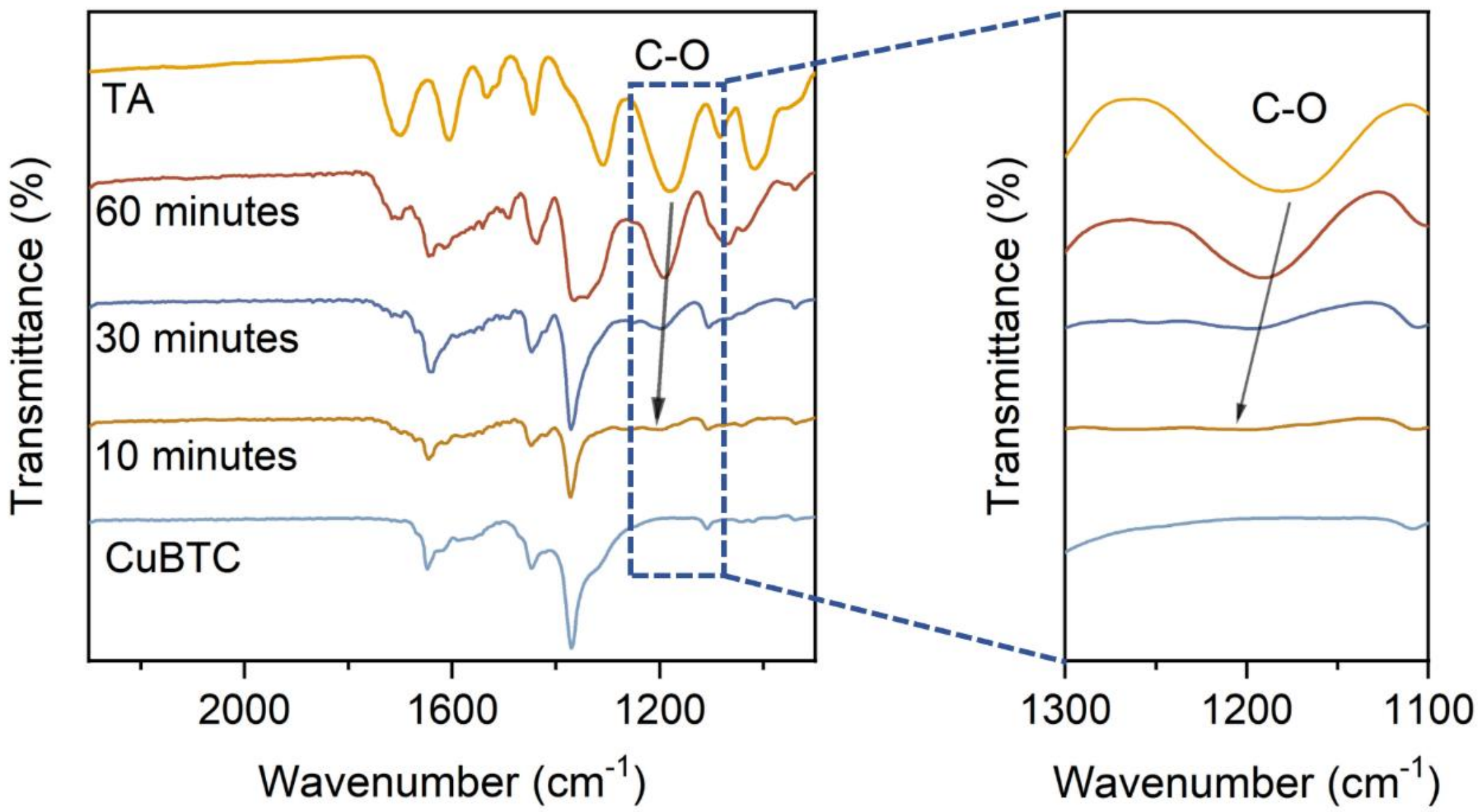

Fig. S11. FTIR spectra of TA and TA treated CuBTC membranes.

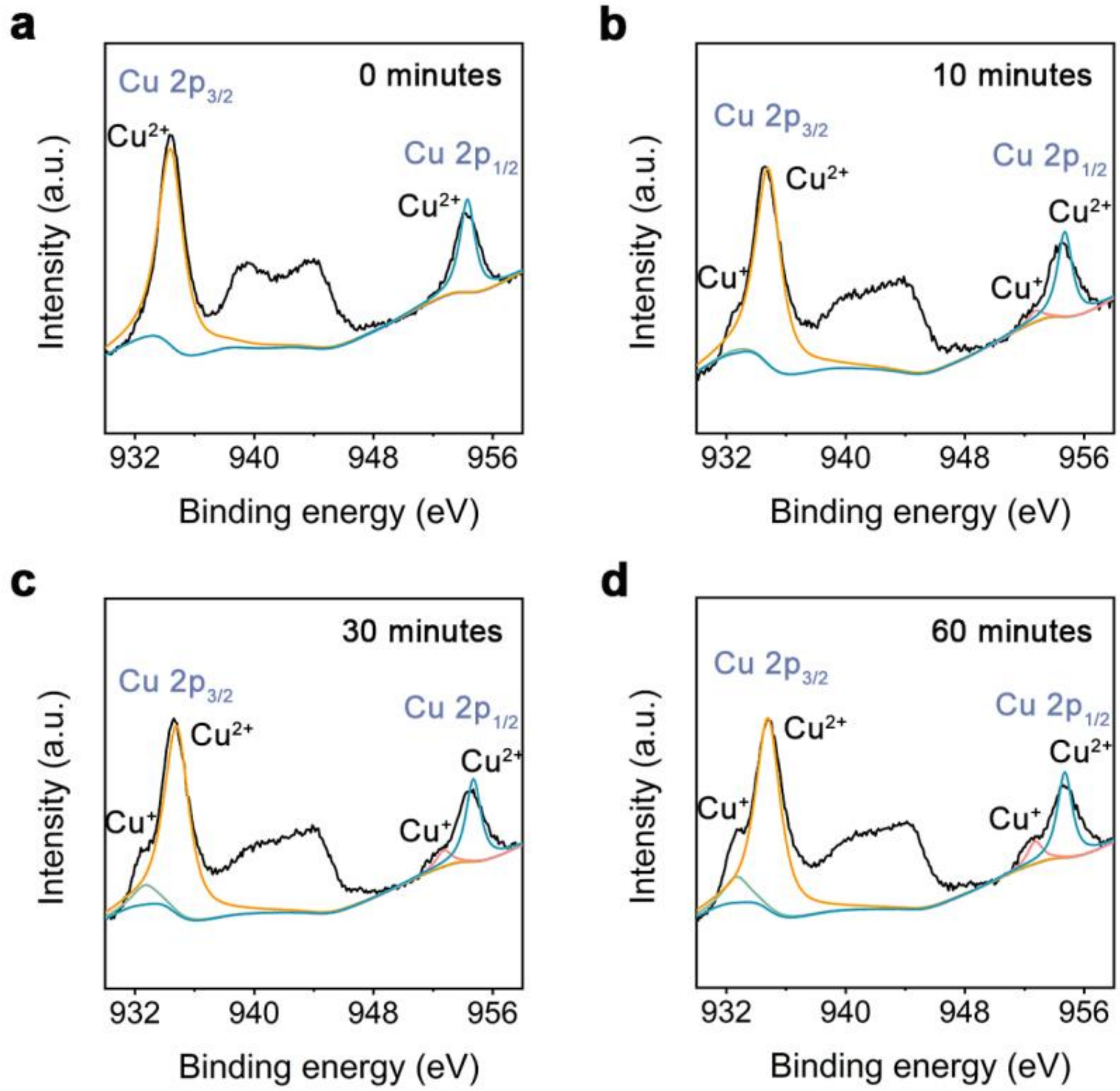

Fig. S12. XPS spectroscopy analysis of Cu 2p of the CuBTC membranes treated using TA with durations ranging from 0 to 60 min.

As illustrated in Fig. S12a, the primary peaks at binding energies of 934.7 eV and 954.7 eV correspond to the Cu $2p_{3/2}$ and Cu $2p_{1/2}$ signals of Cu^{2+} species, respectively. Additional peaks between 939.5 eV and 943.5 eV are attributed to shake-up satellite features [4, 5]. In Figs. S12b to S14d, newly emerged peaks at 932.7 eV and 952.7 eV are assigned to the Cu $2p_{3/2}$ and Cu $2p_{1/2}$ signals of Cu^{+} species. The generation of Cu^{+} is most likely due to the reductive effect of TA. Curve-fitting analyses further reveal that, after 60 min of TA treatment, Cu^{+} accounts for approximately 12% of the total copper content in the CuBTC membrane, indicating that Cu^{2+} remains the dominant oxidation state within the framework.

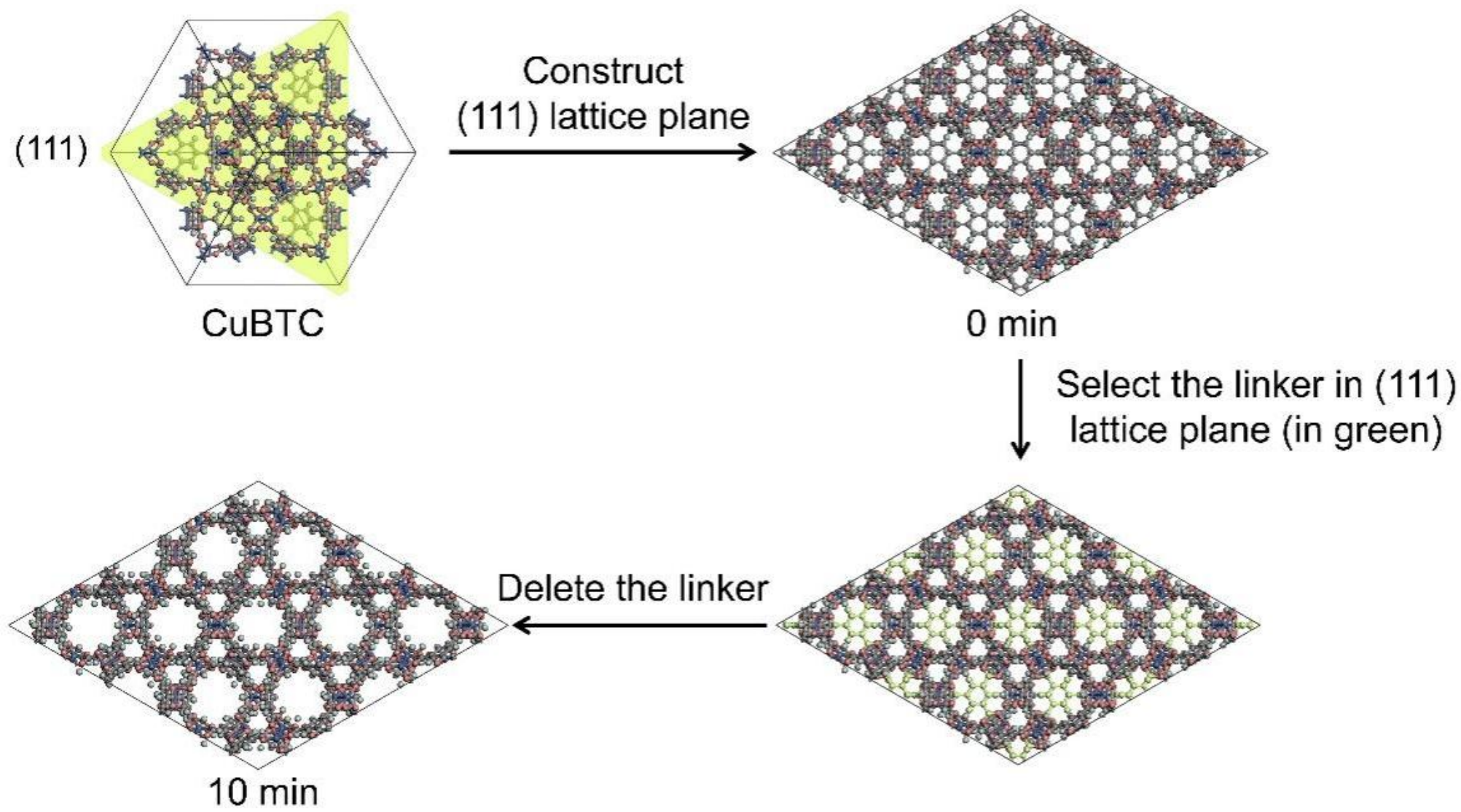

Number of items in the model						
	Cu^{+}	Cu^{2+}	$HBTC^{2-}$	BTC^{3-}	OH^{-}	TA
0 min	0	120	24	64	0	0
10 min	2	118	24	48	46	0

Fig. S13. Schematic illustration of the model construction process for the 10-min treatment.

Given that the CuBTC membrane displays a {111} crystallographic orientation, after the pristine bulk structure was cleaved along the (111) plane, we deleted some irrational fragments to develop a representative surface model. Utilizing this configuration, a two-dimensional MOF slab was constructed with lattice parameters $a = b = 37.3$ Å and $c = 240$ Å, and angles $\alpha = \beta = 90°$ and $\gamma = 120°$. The extended c-axis dimension was incorporated to ensure adequate vacuum spacing, thereby minimizing periodic interactions. The CuBTC model corresponding to the 10-min treatment was obtained from the pristine structure by removing all BTC linkers exposed along the (111) direction, totaling sixteen linkers. After geometry optimization by the Forcite module, the structure after 10-min treatment was obtained.

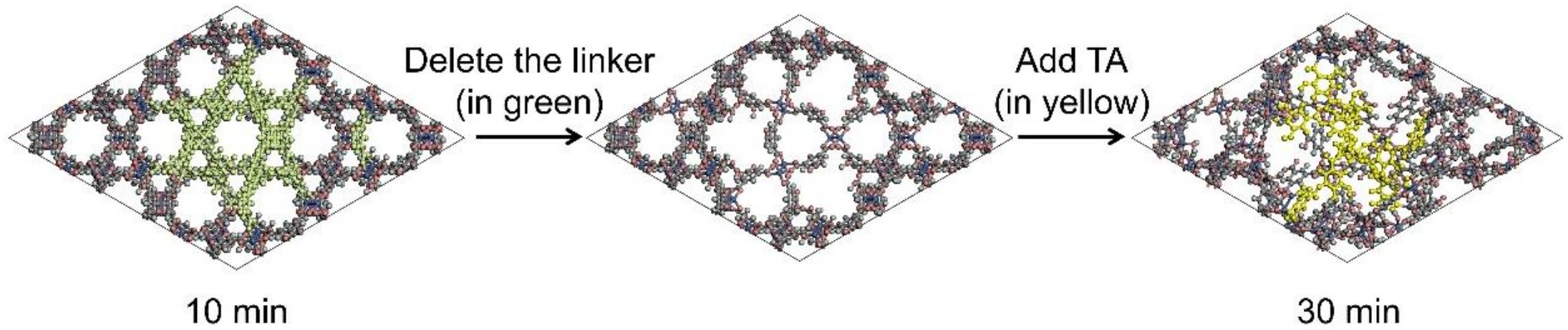

Number of items in the model						
	Cu^{+}	Cu^{2+}	$HBTC^{2-}$	BTC^{3-}	OH^{-}	TA
10 min	2	118	24	48	46	0
30 min	8	78	31	20	42	1

Fig. S14. Schematic illustration of the model construction process for the 30-min treatment.

The CuBTC model representing the 30-min treatment was derived from the 10-min model to depict an intermediate structural state during the TA treatment. Informed by the experimentally determined chemical composition at this stage, a portion of Cu atoms and their coordinated BTC^{3-} linkers were selectively removed from one side of the outer layer of the MOF slab. The framework was then reconstructed by introducing asymmetric coordination defects. Subsequently, a TA molecule was partially incorporated into the central cavity of the slab, allowing four coordination sites of TA to interact with the Cu clusters of the framework via metal-phenol coordination, as discussed in Fig. S18. The framework was obtained after geometry optimization by the Forcite module.

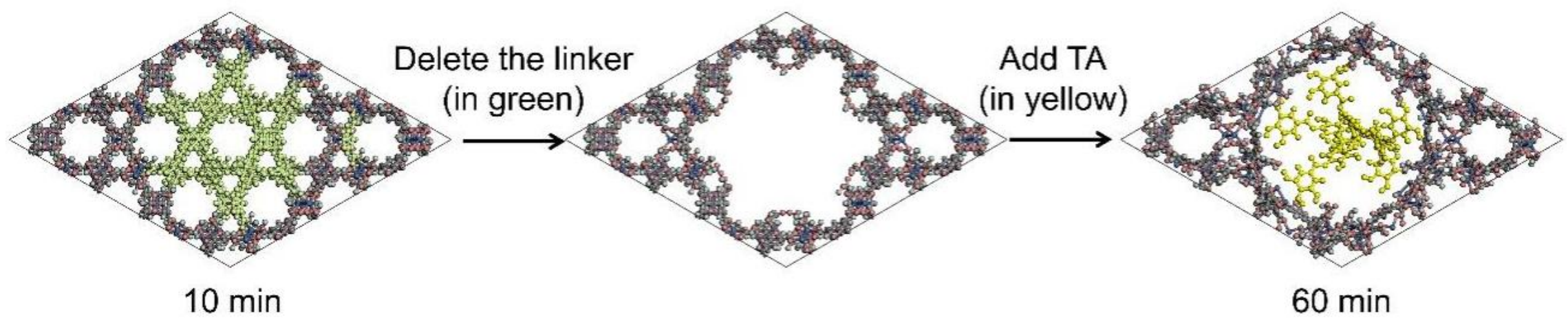

Number of items in the model						
	Cu^{+}	Cu^{2+}	$HBTC^{2-}$	BTC^{3-}	OH^{-}	TA
10 min	2	118	24	48	46	0
60 min	10	74	22	24	42	1

Fig. S15. Schematic illustration of the model construction process for the 60-min treatment.

The CuBTC model corresponding to the 60-min treatment was developed based on the 10-min model to represent the final structural configuration achieved during the TA treatment. In accordance with the experimentally determined chemical composition, a fraction of Cu atoms and their BTC^{3-} linkers were removed from the central region of the MOF slab. A huge cavity was generated due to an increased degree of linker deficiency, thereby exposing additional metal sites to emulate the stable structure formed after extended treatment. Subsequently, a TA molecule was fully incorporated into the central cavity of the slab, with its five coordination sites interacting with the Cu clusters of the framework via metal-phenol coordination, as discussed in Fig. S18. The 60-min framework was obtained after geometry optimization by the Forcite module.

Table S2. Experimental and simulated molecular formula of the CuBTC membrane and TA treated membranes.

Membrane	Experimental molecular formula	Simulated molecular formula
CuBTC	$Cu^{II}_3(BTC)_2$	$Cu^{II}_3(BTC)_2$
TA-treatment for 10 min	$[Cu^{II}_{2.95}Cu^{I}_{0.05}(BTC)_{1.79}(TA)_{0.010}(OH)_x]$	$[Cu^{II}_{2.95}Cu^{I}_{0.05}(BTC/HBTC)_{1.80}(OH)_{1.15}]$
TA-treatment for 30 min	$[Cu^{II}_{2.72}Cu^{I}_{0.28}(BTC)_{1.76}(TA)_{0.028}(OH)_x]$	$[Cu^{II}_{2.72}Cu^{I}_{0.28}(BTC/HBTC)_{1.78}(TA)_{0.035}(OH)_{1.47}]$
TA-treatment for 60 min	$[Cu^{II}_{2.64}Cu^{I}_{0.36}(BTC)_{1.62}(TA)_{0.041}(OH)_x]$	$[Cu^{II}_{2.64}Cu^{I}_{0.36}(BTC/HBTC)_{1.64}(TA)_{0.036}(OH)_{1.50}]$

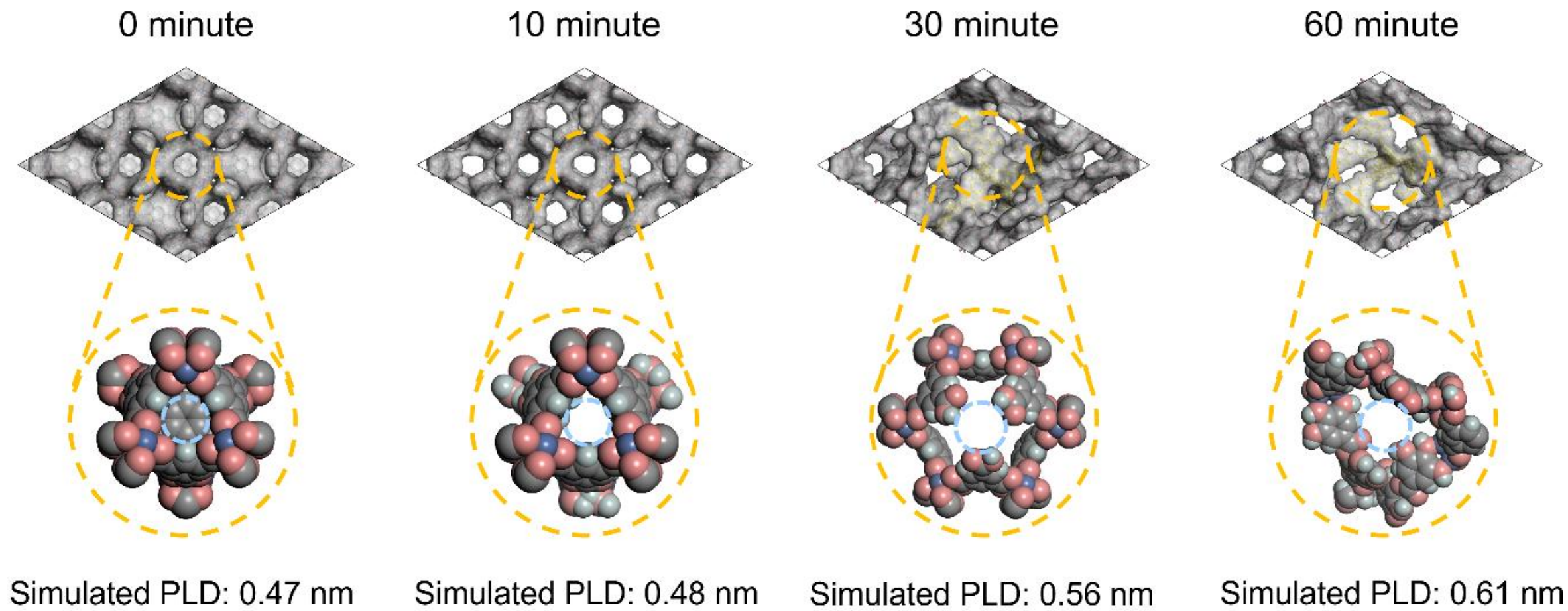

Fig. S16. Structural modelling and PLD simulation of CuBTC treated using TA with durations ranging from 0 to 60 min.

Table S3. Experimental single-component gas permeance at 298 K and NKP coefficient of the CuBTC membrane.

Component	Molecular weight (g mol^{-1})	Molecular size* (nm)	Experimental permeance (mol m^{-2} s^{-1} Pa^{-1})	NKP coefficient
H_2	2	0.29	6.12E-06	1.00
CH_4	16	0.38	2.16E-06	0.116
C_2H_6	30	0.42	1.58E-06	0.0639
C_3H_8	44	0.43	1.30E-06	0.0621
n-C_4H_{10}	58	0.47	1.14E-06	0.0489

* Molecular size is represented by kinetic diameter.

Table S4. Experimental single-component gas permeance at 298 K and NKP coefficient of CuBTC membrane after TA-treatment for 10 min.

Component	Molecular weight (g mol^{-1})	Molecular size* (nm)	Real permeance (mol m^{-2} s^{-1} Pa^{-1})	NKP coefficient
H_2	2	0.29	6.78E-06	1.00
CH_4	16	0.38	2.40E-06	0.179
C_2H_6	30	0.42	1.75E-06	0.0697
C_3H_8	44	0.43	1.45E-06	0.0654
n-C_4H_{10}	58	0.47	1.26E-06	0.0508

* Molecular size is represented by kinetic diameter.

Table S5. Experimental single-component gas permeance at 298 K and NKP coefficient of CuBTC membrane after TA-treatment for 30 min.

Component	Molecular weight (g mol^{-1})	Molecular size* (nm)	Real permeance (mol m^{-2} s^{-1} Pa^{-1})	NKP coefficient
H_2	2	0.29	6.94E-06	1.00
CH_4	16	0.38	2.45E-06	0.282
C_2H_6	30	0.42	1.79E-06	0.0804
C_3H_8	44	0.43	1.48E-06	0.0716
n-C_4H_{10}	58	0.47	1.29E-06	0.0664

* Molecular size is represented by kinetic diameter.

Table S6. Experimental single-component gas permeance at 298 K and NKP coefficient of CuBTC membrane after TA-treatment for 60 min.

Component	Molecular weight ($g\ mol^{-1}$)	Molecular size* (nm)	Real permeance ($mol\ m^{-2}\ s^{-1}\ Pa^{-1}$)	NKP coefficient
H_2	2	0.29	7.01E-06	1.00
CH_4	16	0.38	2.48E-06	0.352
C_2H_6	30	0.42	1.81E-06	0.143
C_3H_8	44	0.43	1.49E-06	0.139
n-C_4H_{10}	58	0.47	1.30E-06	0.127

* Molecular size is represented by kinetic diameter.

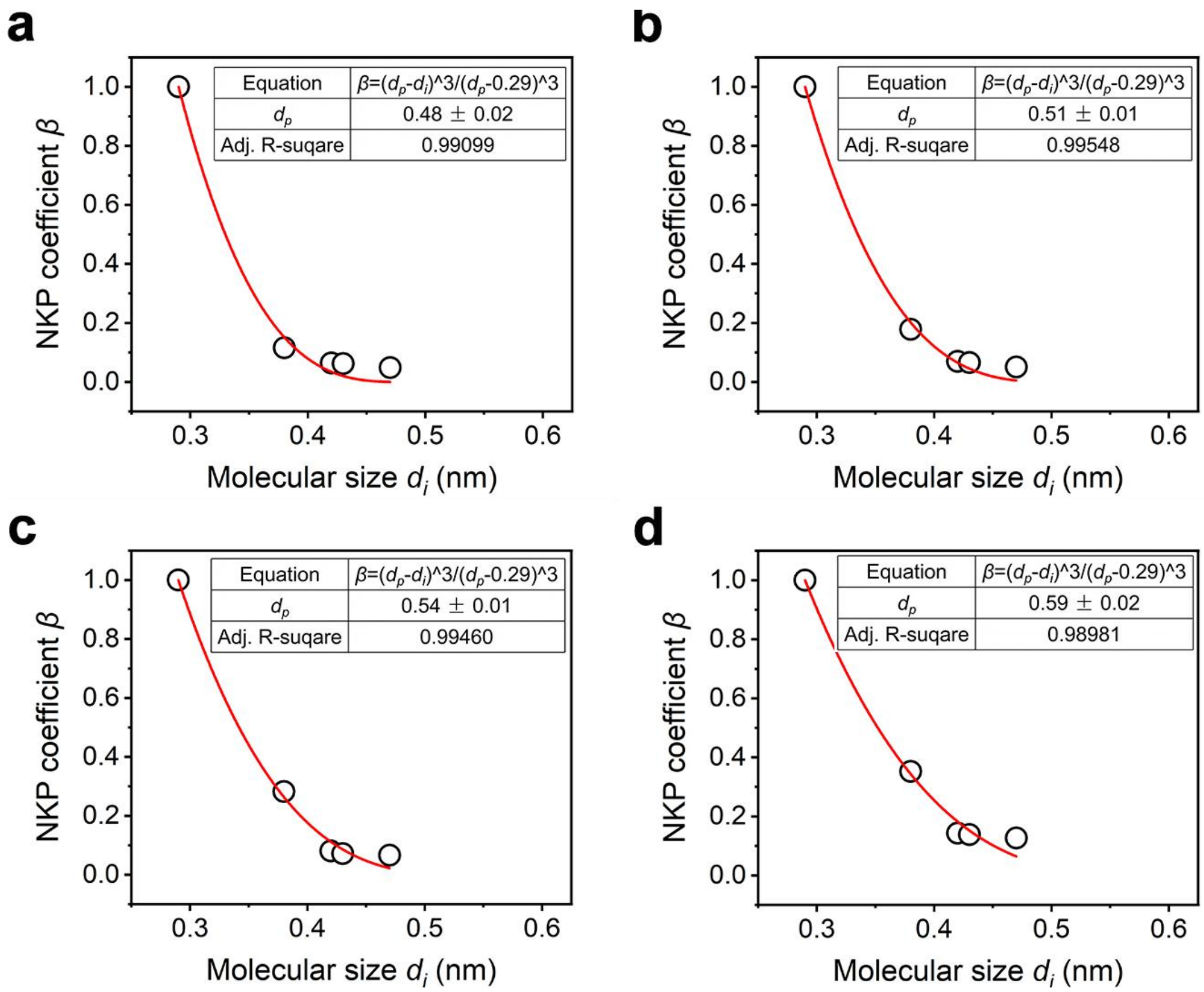

Fig. S17. The NKP coefficient (β) of the membranes as a function of molecular size (d_i). (a) CuBTC membrane. (b to d) TA-treatment for (b) 10 min, (c) 30 min and (d) 60 min.

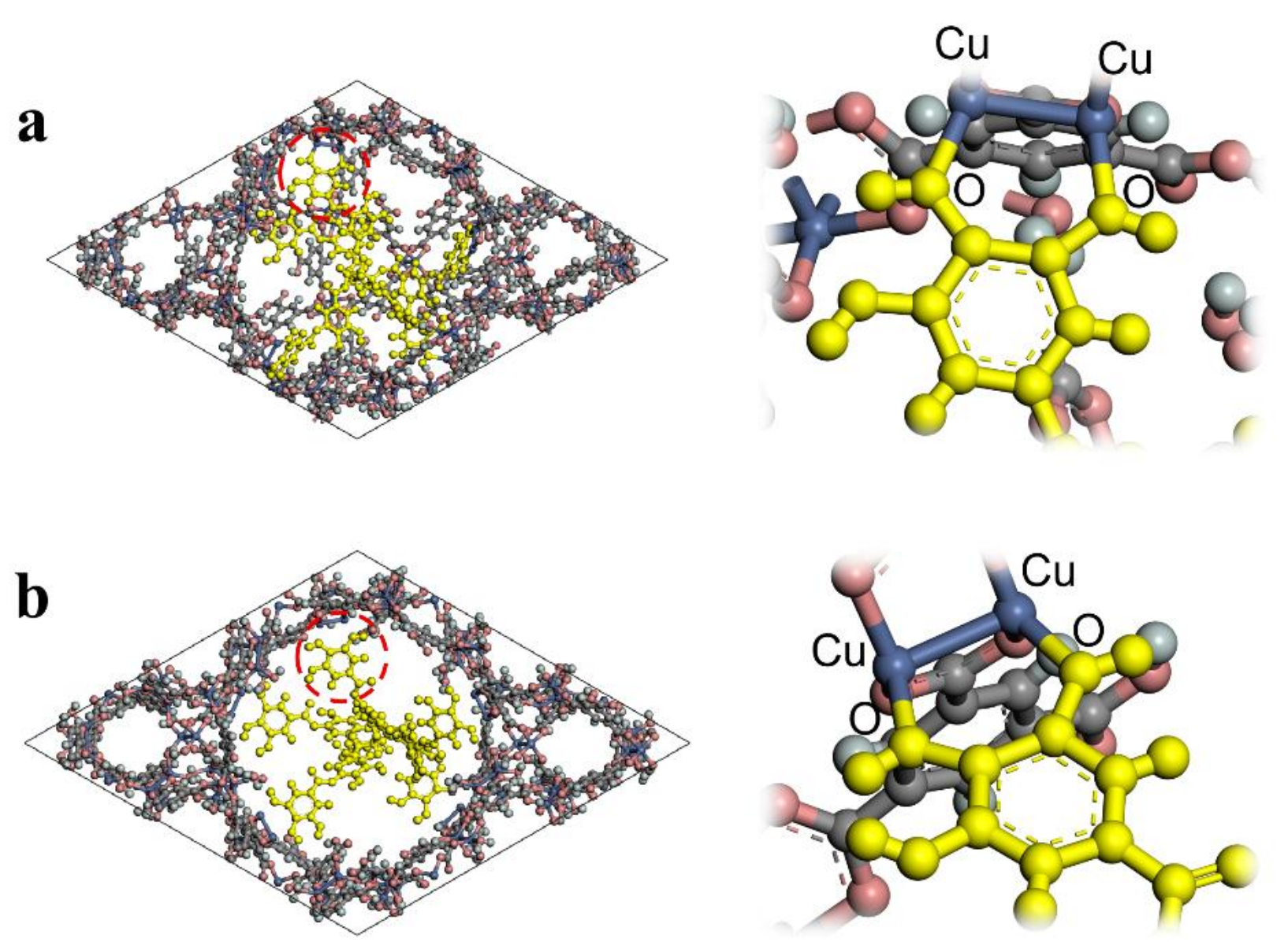

Fig. S18. Structural models illustrating interactions between Cu nodes and the phenolic hydroxyl groups of TA. (a) The CuBTC membrane following 30-min treatment with TA. (b) The CuBTC membrane following 60-min treatment with TA.

In both models, TA molecules interact with exposed Cu nodes via phenolic hydroxyl groups. This metal–phenol coordination mechanism facilitates the stable integration of TA within the MOF cavities formed as a result of linker deficiencies. The interactions between TA and Cu were confirmed via experimental FTIR spectroscopy studies (Fig. S11) and XPS analyses (Fig. S19).

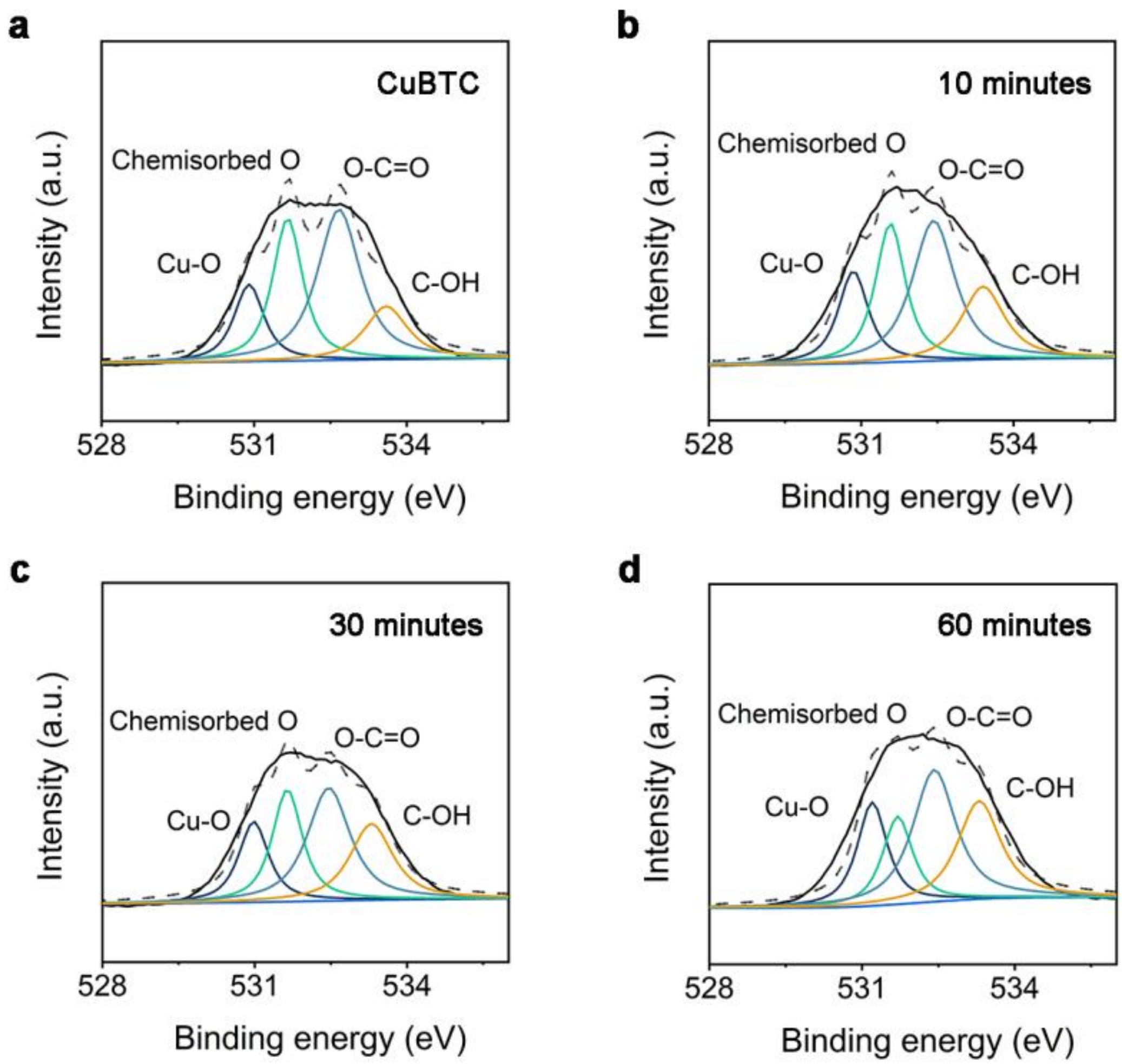

Fig. S19. XPS spectroscopy analysis of O 1s of the CuBTC membranes treated using TA with durations ranging from 0 to 60 min.

As the TA treatment time was increased from 0 to 60 min, pronounced changes in the relative distribution of oxygen species were observed in the XPS O 1s spectra. Specifically, the peak area corresponding to C–OH species increased from 14.85% (CuBTC) to 26.94% (TA treatment 60 min), arising from the increasing incorporation of TA within the membrane structure with prolonged treatment time. Meanwhile, the peak area fraction assigned to Cu–O species increased gradually from 15.09% to 19.93%, which is attributed to progressively strengthened interactions between TA molecules and Cu nodes via metal–phenol coordination.

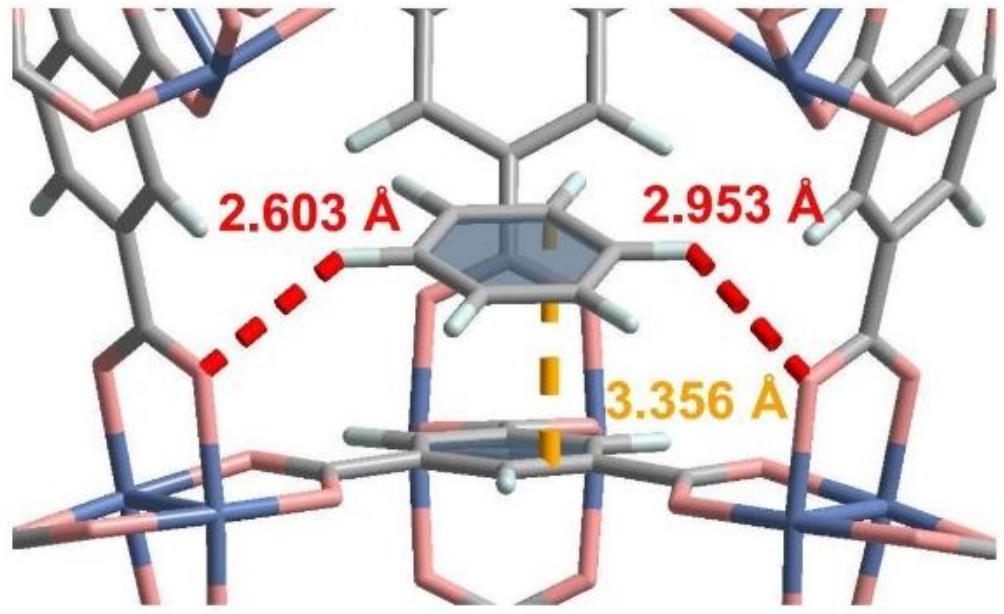

Binding energy = -61.0 kJ mol^{-1}

Host	Guest	Type	The shortest distance (Å)	
CuBTC	BEN	C–H_{host}···O_{guest}	2.603	2.953
		$\pi_{hostr}\cdots\pi_{guest}$	3.356	

Fig. S20. Simulation of adsorption sites of BEN molecules within the pristine CuBTC lattice.

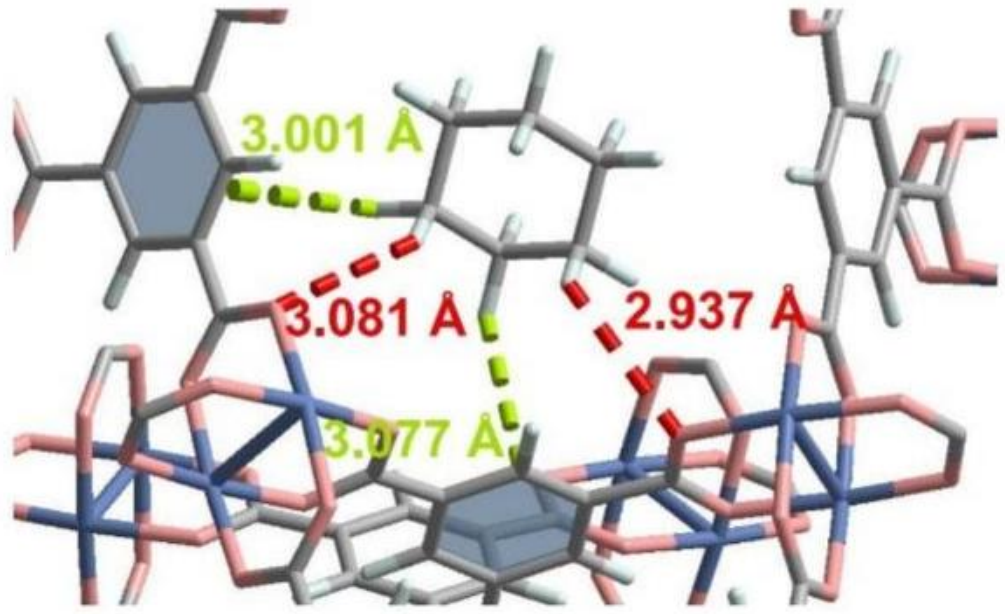

Binding energy = -49.2 kJ mol^{-1}

Host	Guest	Type	The shortest distance (Å)	
CuBTC	CH	C–H$_{guest}$···O$_{host}$	2.937	3.001
		C–H$_{guest}$···π$_{host}$	3.081	3.077

Fig. S21. Simulation of adsorption sites of BEN molecules within the pristine CuBTC lattice.

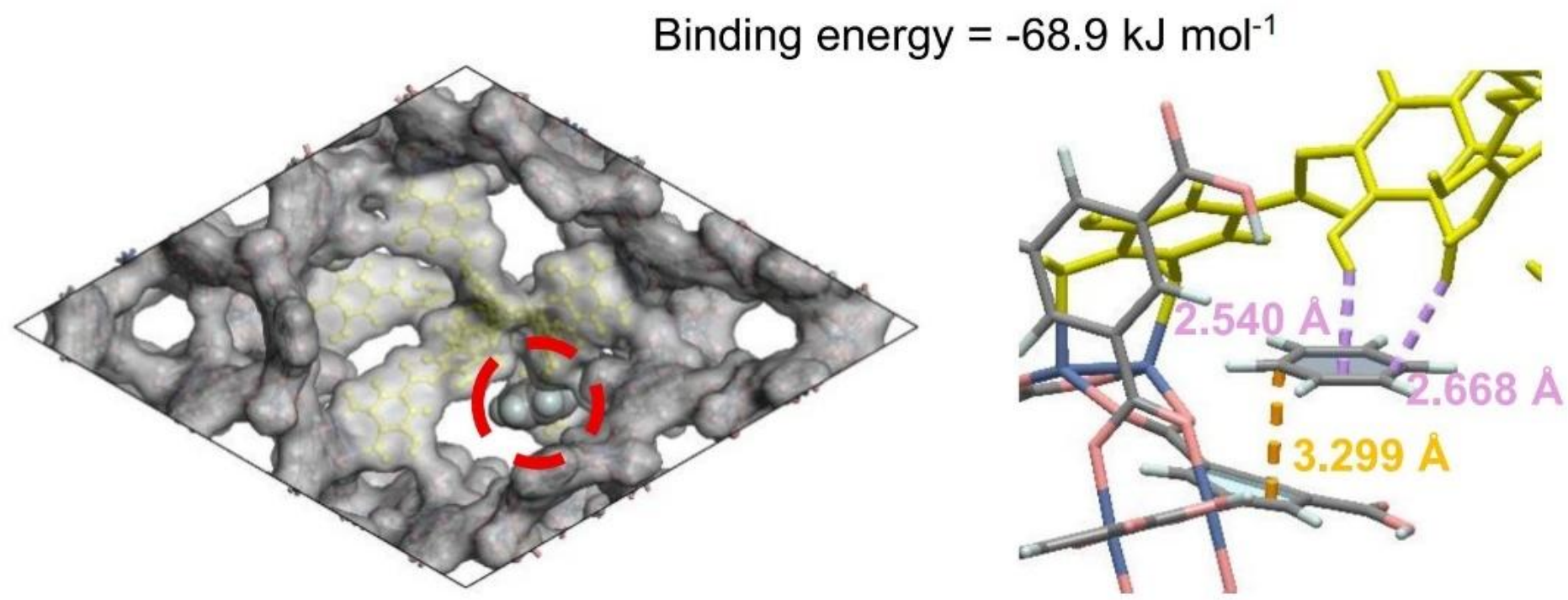

Host	Guest	Type	The shortest distance (Å)	
60 minutes	BEN	$O–H_{host, TA}\cdots\pi_{guest}$	2.540	2.668
		$\pi_{host, linker}\cdots\pi_{guest}$	3.299	

Fig. S22. Simulation of adsorption sites of BEN molecules within the CuBTC membrane slab following TA-treatment for 60 min.

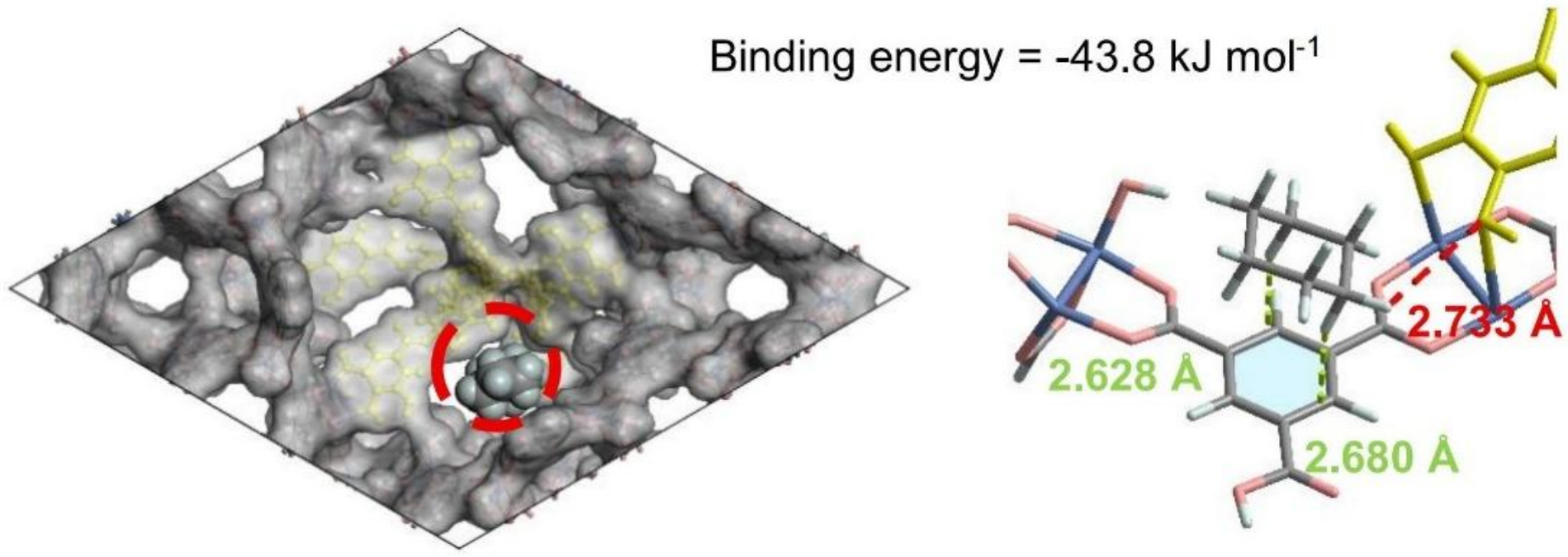

Host	Guest	Type	The shortest distance (Å)	
60 minutes	CH	$C–H_{guest}···π_{host, linker}$	2.628	2.680
		$C–H_{guest}···O_{host, TA}$	2.733	

Fig. S23. Simulation of adsorption sites of CH molecules within the CuBTC membrane slab following TA-treatment for 60 min.

Table S7. Single-component permeance of CuBTC membrane.

Temperature (K)	HEX	2MP	23DMB (LPU)	BEN	CH
303	140	12.5	8.86	8.98	8.48
333	151	13.1	9.10	9.15	8.56

Table S8. Single-component adsorption amount of the CuBTC membrane.

Temperature (K)	HEX	2MP	23DMB (mmol)	BEN	CH
303	0.1283	0.0945	0.0845	0.0842	0.0805
333	0.1105	0.0793	0.0685	0.0676	0.0636

Table S9. Ideal permeation selectivity, adsorption selectivity and diffusion selectivity of the CuBTC membrane.

Ideal permeation selectivity									
Temperature (K)	HEX/2MP	HEX/23DMB	HEX/CH	HEX/BEN	2MP/23DMB	2MP/CH	2MP/BEN	BEN/23DMB	BEN/CH
303	11.21	15.81	17.67	15.59	1.41	1.47	1.39	1.01	1.06
333	11.54	16.62	17.83	16.53	1.44	1.53	1.43	1.01	1.07
Adsorption selectivity									
Temperature (K)	HEX/2MP	HEX/23DMB	HEX/CH	HEX/BEN	2MP/23DMB	2MP/CH	2MP/BEN	BEN/23DMB	BEN/CH
303	1.36	1.52	1.59	1.52	1.12	1.18	1.12	1.00	1.05
333	1.39	1.61	1.74	1.63	1.16	1.25	1.17	0.99	1.06
Diffusion selectivity									
Temperature (K)	HEX/2MP	HEX/23DMB	HEX/CH	HEX/BEN	2MP/23DMB	2MP/CH	2MP/BEN	BEN/23DMB	BEN/CH
303	8.27	10.42	10.10	10.24	1.26	1.25	1.24	1.02	1.01
333	8.31	10.30	10.10	10.11	1.24	1.23	1.22	1.02	1.01

Table S10. Diffusion entropy selectivity and diffusion enthalpy selectivity of the CuBTC membrane for different two-component systems at 333 K.

Component	Diffusion entropic selectivity	Diffusion enthalpic selectivity	Size difference (nm)
HEX/2MP	8.48	0.98	0.07
HEX/23DMB	9.21	1.12	0.15
HEX/CH	8.35	1.22	0.17
HEX/BEN	8.87	1.14	0.16
2MP/23DMB	1.09	1.14	0.08
2MP/CH	0.99	1.24	0.10
2MP/BEN	1.05	1.16	0.09
BEN/23DMB	0.96	1.02	0.01
BEN/CH	0.94	1.07	0.01

Table S11. Single-component permeance of CuBTC membrane following 10-min treatment with TA.

Temperature (K)	HEX	2MP	23DMB (LPU)	BEN	CH
303	154	109	18.6	12.1	16.7
333	166	132	19.8	13.9	18.8

Table S12. Single component adsorption amount of CuBTC membrane following 10-min treatment with TA.

Temperature (K)	HEX	2MP	23DMB (mmol)	BEN	CH
303	0.1327	0.1205	0.1062	0.0955	0.1007
333	0.1215	0.1234	0.0951	0.0913	0.0948

Table S13. Ideal permeation selectivity, adsorption selectivity and diffusion selectivity of CuBTC membrane following 10-min treatment with TA.

Ideal permeation selectivity									
Temperature (K)	HEX/2MP	HEX/23DMB	HEX/CH	HEX/BEN	2MP/23DMB	2MP/CH	2MP/BEN	BEN/23DMB	BEN/CH
303	1.42	8.28	9.00	12.79	5.83	6.55	9.00	0.65	0.72
333	1.26	8.38	9.25	11.94	6.65	6.73	9.48	0.70	0.74
Adsorption selectivity									
Temperature (K)	HEX/2MP	HEX/23DMB	HEX/CH	HEX/BEN	2MP/23DMB	2MP/CH	2MP/BEN	BEN/23DMB	BEN/CH
303	1.11	1.25	1.32	1.39	1.13	1.20	1.26	0.90	0.95
333	0.98	1.28	1.28	1.33	1.30	1.30	1.35	0.96	0.96
Diffusion selectivity									
Temperature (K)	HEX/2MP	HEX/23DMB	HEX/CH	HEX/BEN	2MP/23DMB	2MP/CH	2MP/BEN	BEN/23DMB	BEN/CH
303	1.29	6.62	6.91	8.20	5.14	5.43	7.14	0.72	0.76
333	1.28	6.56	7.00	8.97	5.13	5.50	7.02	0.73	0.77

Table S14. Diffusion entropy selectivity and diffusion enthalpy selectivity of CuBTC membrane following 10-min treatment with TA for different two-component systems at 333 K.

Component	Diffusion entropic selectivity	Diffusion enthalpic selectivity	Size difference (nm)
HEX/2MP	1.17	1.09	0.07
HEX/23DMB	5.93	1.11	0.15
HEX/CH	5.89	1.17	0.17
HEX/BEN	6.91	1.30	0.16
2MP/23DMB	5.06	1.01	0.08
2MP/CH	5.03	1.07	0.10
2MP/BEN	5.89	1.19	0.09
BEN/23DMB	0.86	0.85	0.01
BEN/CH	0.85	0.90	0.01

Table S15. Single-component permeance of CuBTC membrane following 30-min treatment with TA.

Temperature (K)	HEX	2MP	23DMB (LPU)	BEN	CH
303	177	131	25.3	83.2	21.4
333	188	138	25.7	105	24.5

Table S16. Single component adsorption amount of CuBTC membrane following 30-min treatment with TA.

Temperature (K)	HEX	2MP	23DMB (mmol)	BEN	CH
303	0.1435	0.1226	0.1165	0.1194	0.1120
333	0.1260	0.1087	0.0987	0.1053	0.1007

Table S17. Ideal permeation selectivity, adsorption selectivity and diffusion selectivity of CuBTC membrane following 30-min treatment with TA.

Ideal permeation selectivity									
Temperature (K)	HEX/2MP	HEX/23DMB	HEX/CH	HEX/BEN	2MP/23DMB	2MP/CH	2MP/BEN	BEN/23DMB	BEN/CH
303	1.34	6.99	8.27	1.58	5.20	6.13	1.18	4.42	5.21
333	1.33	7.15	7.56	1.74	5.38	5.67	1.31	4.10	4.31
Adsorption selectivity									
Temperature (K)	HEX/2MP	HEX/23DMB	HEX/CH	HEX/BEN	2MP/23DMB	2MP/CH	2MP/BEN	BEN/23DMB	BEN/CH
303	1.17	1.23	1.29	1.20	1.05	1.10	1.03	1.02	1.07
333	1.16	1.28	1.25	1.20	1.10	1.08	1.03	1.07	1.05
Diffusion selectivity									
Temperature (K)	HEX/2MP	HEX/23DMB	HEX/CH	HEX/BEN	2MP/23DMB	2MP/CH	2MP/BEN	BEN/23DMB	BEN/CH
303	1.15	5.68	6.43	1.32	4.94	5.62	1.15	4.31	4.89
333	1.15	5.60	5.97	1.45	4.88	5.22	1.27	3.85	4.12

Table S18. Diffusion entropy selectivity and diffusion enthalpy selectivity of CuBTC membrane following 30-min treatment with TA for different two-component systems at 333 K.

Component	Diffusion entropic selectivity	Diffusion enthalpic selectivity	Size difference (nm)
HEX/2MP	1.12	1.02	0.07
HEX/23DMB	4.85	1.15	0.15
HEX/CH	4.05	1.21	0.17
HEX/BEN	4.01	0.36	0.16
2MP/23DMB	4.33	1.13	0.08
2MP/CH	3.62	1.18	0.10
2MP/BEN	3.58	0.35	0.09
BEN/23DMB	1.21	3.18	0.01
BEN/CH	1.01	3.34	0.01

Table S19. Single-component permeance of CuBTC membrane following 60-min treatment with TA.

Temperature (K)	HEX	2MP	23DMB (LPU)	BEN	CH
303	181	136	33.9	167	26.4
333	196	141	36.0	171	30.1

Table S20. Single component adsorption amount of CuBTC membrane following 60-min treatment with TA.

Temperature (K)	HEX	2MP	23DMB (mmol)	BEN	CH
303	0.1378	0.1236	0.1102	0.1408	0.1117
333	0.1329	0.1181	0.1034	0.1473	0.0979

Table S21. Ideal permeation selectivity, adsorption selectivity and diffusion selectivity of CuBTC membrane following 60-min treatment with TA.

Ideal permeation selectivity									
Temperature (K)	HEX/2MP	HEX/23DMB	HEX/CH	HEX/BEN	2MP/23DMB	2MP/CH	2MP/BEN	BEN/23DMB	BEN/CH
303	1.36	5.33	6.86	1.11	3.91	5.02	0.81	4.79	6.17
333	1.39	5.44	6.53	1.15	3.92	4.69	0.83	4.74	5.68
Adsorption selectivity									
Temperature (K)	HEX/2MP	HEX/23DMB	HEX/CH	HEX/BEN	2MP/23DMB	2MP/CH	2MP/BEN	BEN/23DMB	BEN/CH
303	1.12	1.25	1.24	0.98	1.12	1.11	0.88	1.28	1.26
333	1.13	1.29	1.36	0.90	1.14	1.21	0.80	1.43	1.51
Diffusion selectivity									
Temperature (K)	HEX/2MP	HEX/23DMB	HEX/CH	HEX/BEN	2MP/23DMB	2MP/CH	2MP/BEN	BEN/23DMB	BEN/CH
303	1.22	4.26	5.56	1.14	3.48	4.53	0.93	3.75	4.89
333	1.23	4.23	4.77	1.27	3.43	3.89	1.03	3.33	3.77

Table S22. Diffusion entropy selectivity and diffusion enthalpy selectivity of CuBTC membrane following 60-min treatment with TA for different two-component systems at 333 K.

Component	Diffusion entropic selectivity	Diffusion enthalpic selectivity	Size difference (nm)
HEX/2MP	1.33	0.93	0.07
HEX/23DMB	3.94	1.08	0.15
HEX/CH	4.18	1.30	0.17
HEX/BEN	4.00	0.32	0.16
2MP/23DMB	2.96	1.16	0.08
2MP/CH	3.15	1.39	0.10
2MP/BEN	3.01	0.34	0.09
BEN/23DMB	0.98	3.38	0.01
BEN/CH	1.05	4.07	0.01

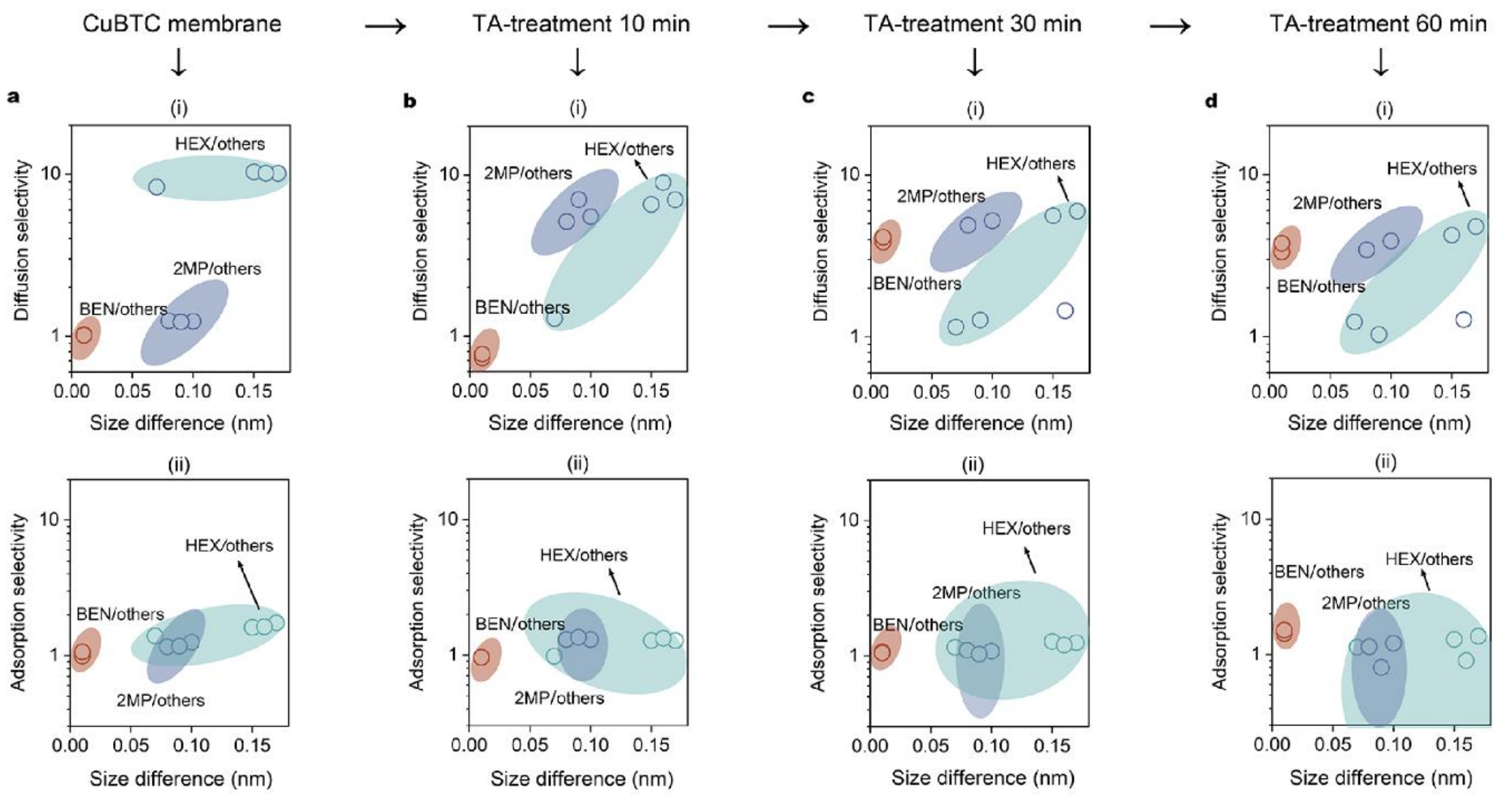

Fig. S24. Diffusion and adsorption selectivity of membranes at 333 K, analyzed in relation to the differences in molecular size among representative components of light naphtha.

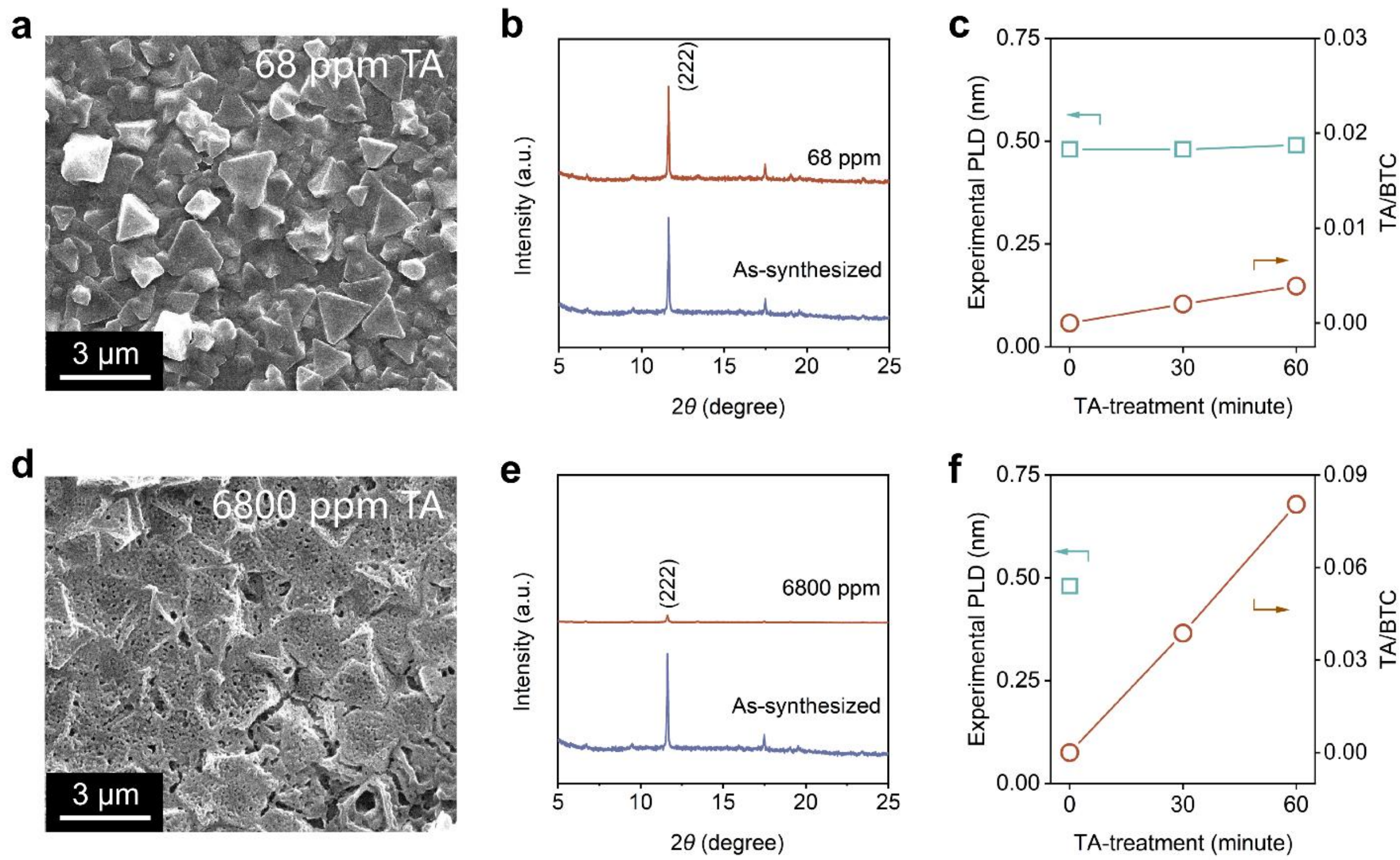

Fig. S25. Characterization of CuBTC membranes treated with dilute (68 ppm) and concentrated (6800 ppm) TA solutions. (a) Top-view SEM image of the CuBTC membrane after treatment with 68-ppm TA for 60 min. (b) PXRD patterns of the CuBTC membrane after treatment with 68 ppm-TA for 60 min. (c) Experimental PLD of CuBTC membranes as a function of treatment time. (d) Top-view SEM image of the CuBTC membrane after treatment with a 6800-ppm TA for 60 min. (e) PXRD patterns of CuBTC membranes before and after treatment with a 6800-ppm TA for 60 min. (f) Experimental PLD of CuBTC membranes as a function of treatment time in a 6800 ppm TA aqueous solution.

At 68 ppm, SEM images revealed only slight modification at the crystal edges (Fig. S25a). The experimental PLD exhibited only a marginal increase from 0.48 nm for the pristine membrane to 0.49 nm after 60 min of treatment (Fig. S25c), indicating negligible pore-size modulation under this condition. In addition, quantitative composition analysis showed a TA/BTC ratio of only 0.39%, suggesting that very limited amounts of TA were incorporated into the framework. These results demonstrate that excessively dilute TA solutions are unable to effectively enlarge the original pores or introduce sufficient TA molecules into the membrane. In contrast, treatment with 6800 ppm TA caused severe damage to the membrane surface, as evidenced by SEM characterization (Fig. S25d). Consistently, the PXRD peaks were dramatically weakened after treatment, confirming significant loss of crystallinity and partial structural collapse of the CuBTC framework (Fig. S25e). Quantitative composition analysis revealed a TA/BTC ratio of 8.05% after 60 min of treatment, indicating that a large amount of TA was incorporated into the framework. However, overly concentrated TA solutions accelerate framework degradation and eventually induce partial structural collapse, making them unsuitable for controlled pore-size modulation despite the increased incorporation of TA molecules.

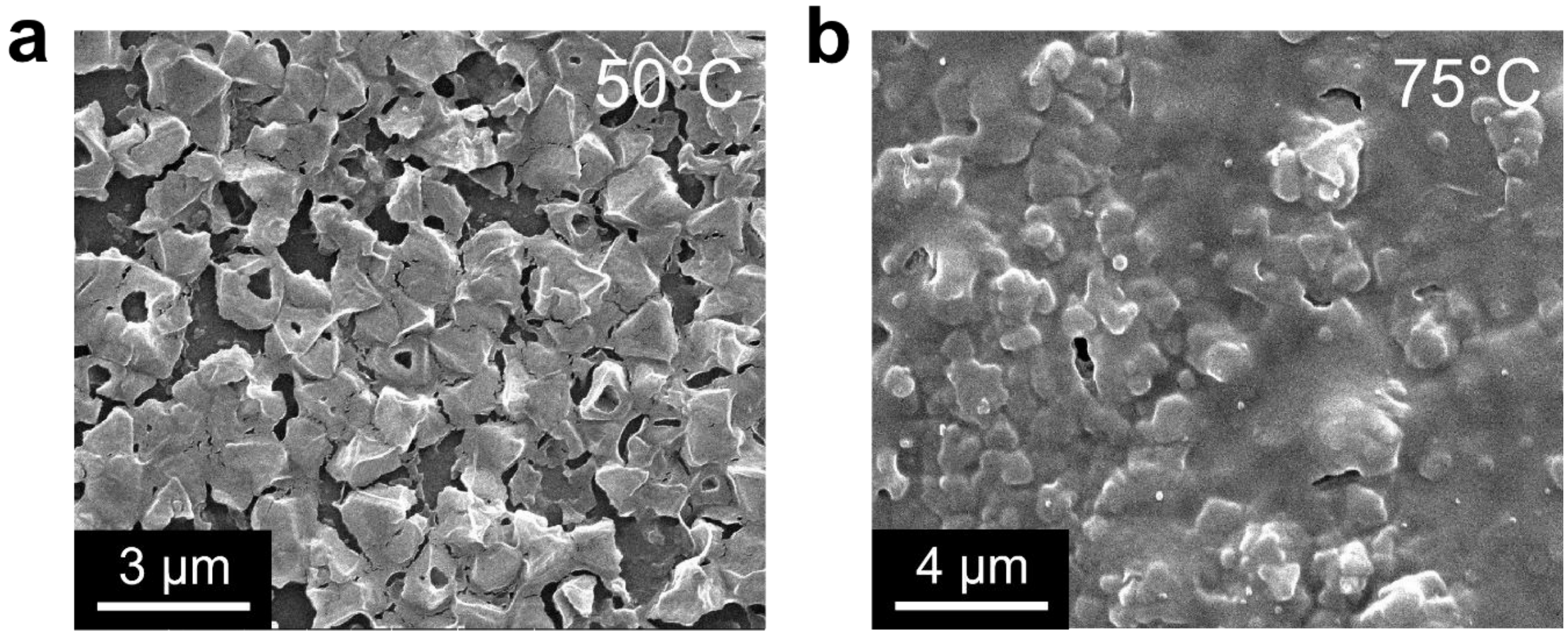

Fig. S26. SEM images of CuBTC membranes treated with 680 ppm of TA solution at different temperatures for 60 min. (a) 50 °C; (b) 75 °C.

Elevated treatment temperatures significantly accelerated framework degradation, resulting in pronounced damage to the membrane surface and crystal boundaries (Fig. S26).

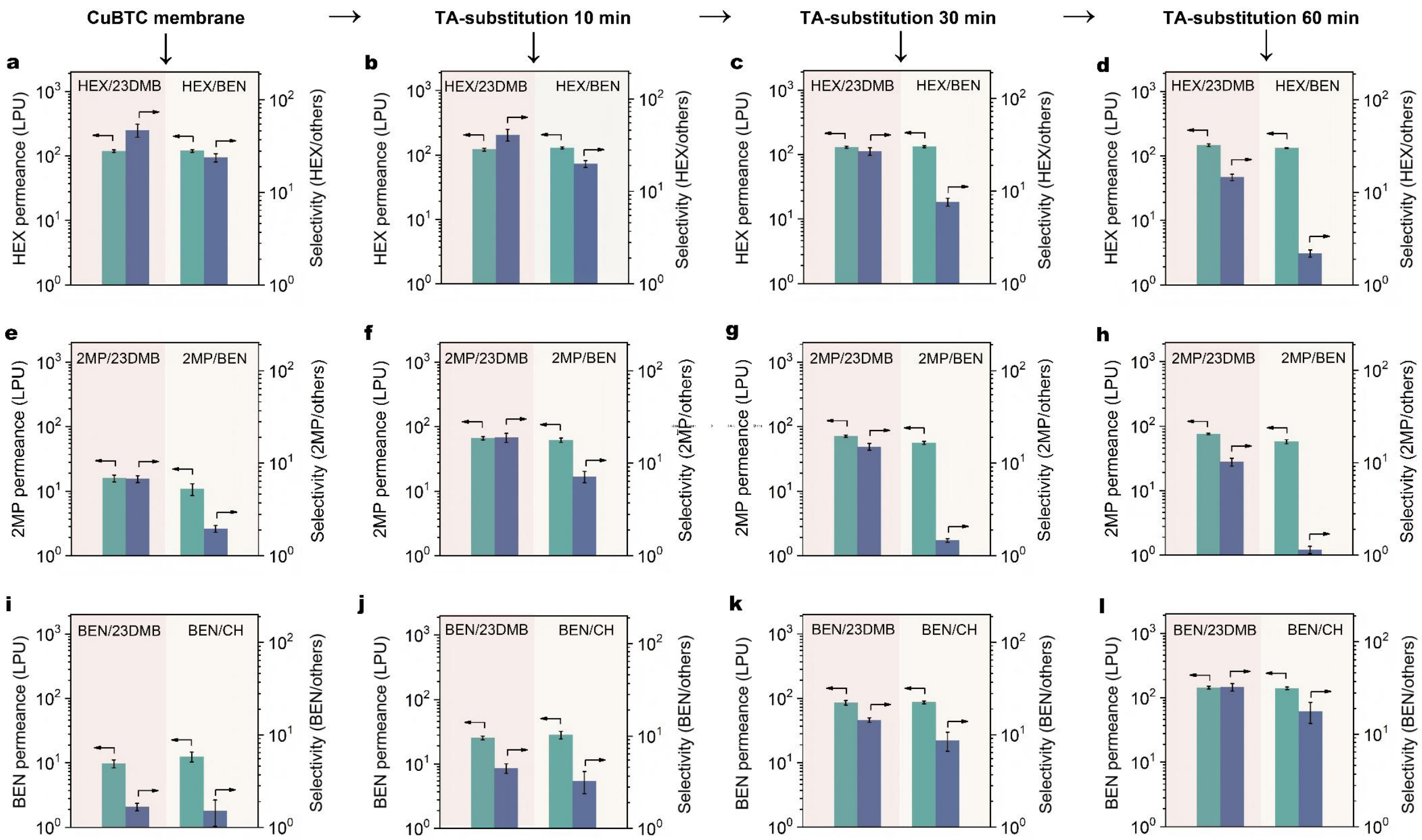

Fig. S27. Separation performance of the CuBTC membrane and TA-treated membranes for dual-component C_6 mixtures at 333 K. Error bars in (a to l) represent the standard deviation of the results from three membranes.

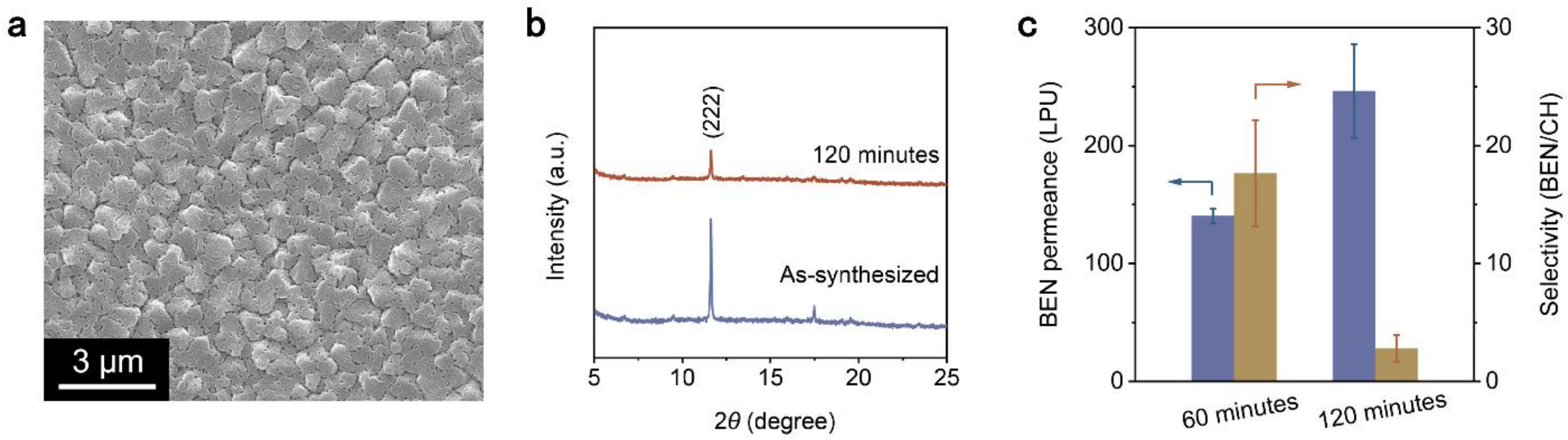

Fig. S28. The structure and separation performance of CuBTC membranes following TA treatment for 120 min. (a) Top-view SEM image. (b) PXRD patterns. (c) Separation performance for the BEN/CH mixture.

Prolonged TA exposure drastically damages intercrystalline boundaries and introduces abundant defects. Consistent with the SEM observation (Fig. S28a), only weak XRD diffraction peaks were detected (Fig. S28b), which indicates severe structural degradation of the membrane framework. This structural deterioration directly explains the sharp performance shift: the BEN permeance nearly doubles (Fig. S28c), while the BEN/CH selectivity falls drastically from 17.6 (60 min TA treatment) to merely 2.8 (120 min TA treatment). Under such conditions, viscous flow through membrane defects dominates mass transport. As a result, the Knudsen gas permeation method cannot reliably quantify the intrinsic membrane pore size.

Table S23. Comparison with other membranes for the separation of linear/multi-branched alkanes mixtures.

Membrane type	Membrane	T (°C)	Feed solution	HEX permeance (LPU)	Selectivity	References
Zeolite membrane	ZSM-5	190	50/50 HEX/22DMB	150	18.0	[6]
Zeolite membrane	Silicalite	190	50/50 HEX/22DMB	18.4	22.5	[7]
Mixed matrix membrane	UiO-66/polymer of intrinsic microporosity-1	40	50/50 HEX/22DMB	254	6.18	[8]
MOF membrane	Al-bttotb	25	50/50 HEX/22DMB	11.1	7.73	[9]
MOF membrane	CuBTC	60	50/50 HEX/22DMB	123	53.4	This work
MOF membrane	CuBTC	60	50/50 HEX/23DMB	119	46.9	This work

Table S24. Comparison with other membranes for the separation of mono-/multi-branched alkanes mixtures.

Membrane type	Membrane	T (°C)	Feed solution	2MP permeance (LPU)	Selectivity	References
Mixed matrix membrane	UiO-66/PIM	40	50/50 2MP/22 DMB	348	6.90	[8]
MOF membrane	Al-bttotb	25	50/50 3MP/22 DMB	3.30	1.69	[9]
MOF membrane	CuBTC	60	50/50 2MP/23 DMB	15.9	6.72	This work
MOF membrane	CuBTC (TA treatment 10 min)	60	50/50 2MP/22 DMB	65.7	23.3	This work
MOF membrane	CuBTC (TA treatment 10 min)	60	50/50 3MP/22 DMB	69.5	24.1	This work
MOF membrane	CuBTC (TA treatment 10 min)	60	50/50 2MP/23 DMB	66.2	18.9	This work

Table S25. Comparison with other membranes for the separation of cyclic/aromatic alkanes mixtures.

Membrane type	Membrane	T (°C)	Feed solution	BEN permeance (LPU)	Selectivity	References
Polymer membrane	Poly(ether-block-amide)	40	50/50 BEN/CH	21.8	3.90	[10]
Polymer membrane	PUU	40	50/50 BEN/CH	70.1	5.85	[11]
Polymer membrane	P(AN-b-MA)	30	50/50 BEN/CH	28.7	9.79	[12]
Polymer membrane	BTAPPPI–IA(PEAI)	50	50/50 BEN/CH	73.2	5.73	[13]
Polymer membrane	BTAPPI–TIPA(PEAI)	50	50/50 BEN/CH	91.7	4.35	[14]
Polymer membrane	BTAPPI–TA(PEAI)	50	50/50 BEN/CH	55.2	6.38	[15]
Ionic liquid membrane	IL-co-PU	50	50/50 BEN/CH	54.7	7.68	[16]
Mixed matrix membrane	W3000	40	50/50 BEN/CH	65.5	3.81	[17]
Mixed matrix membrane	Ag–COF-LZU8/Pebax	40	50/50 BEN/CH	64.9	3.90	[18]
Mixed matrix membrane	Calix/PVAc	37	50/50 BEN/CH	9.96	1.39	[19]
Mixed matrix membrane	Functionalized multiwalled carbon nanotubes/Chitosan	20	50/50 BEN/CH	77.1	3.18	[20]
Mixed matrix membrane	MOP-$SO_3Na_nH_m/Co(OH)_2$	40	50/50 BEN/CH	207	5.20	[21]
Mixed matrix membrane	UiO-66@HBP membrane	40	50/50 BEN/CH	336	10.5	[22]
MOF membrane	CuBTC	60	50/50 BEN/CH	12.5	1.50	This work
MOF membrane	TA-CuBTC (TA treatment 60 min)	60	50/50 BEN/CH	140	17.6	This work

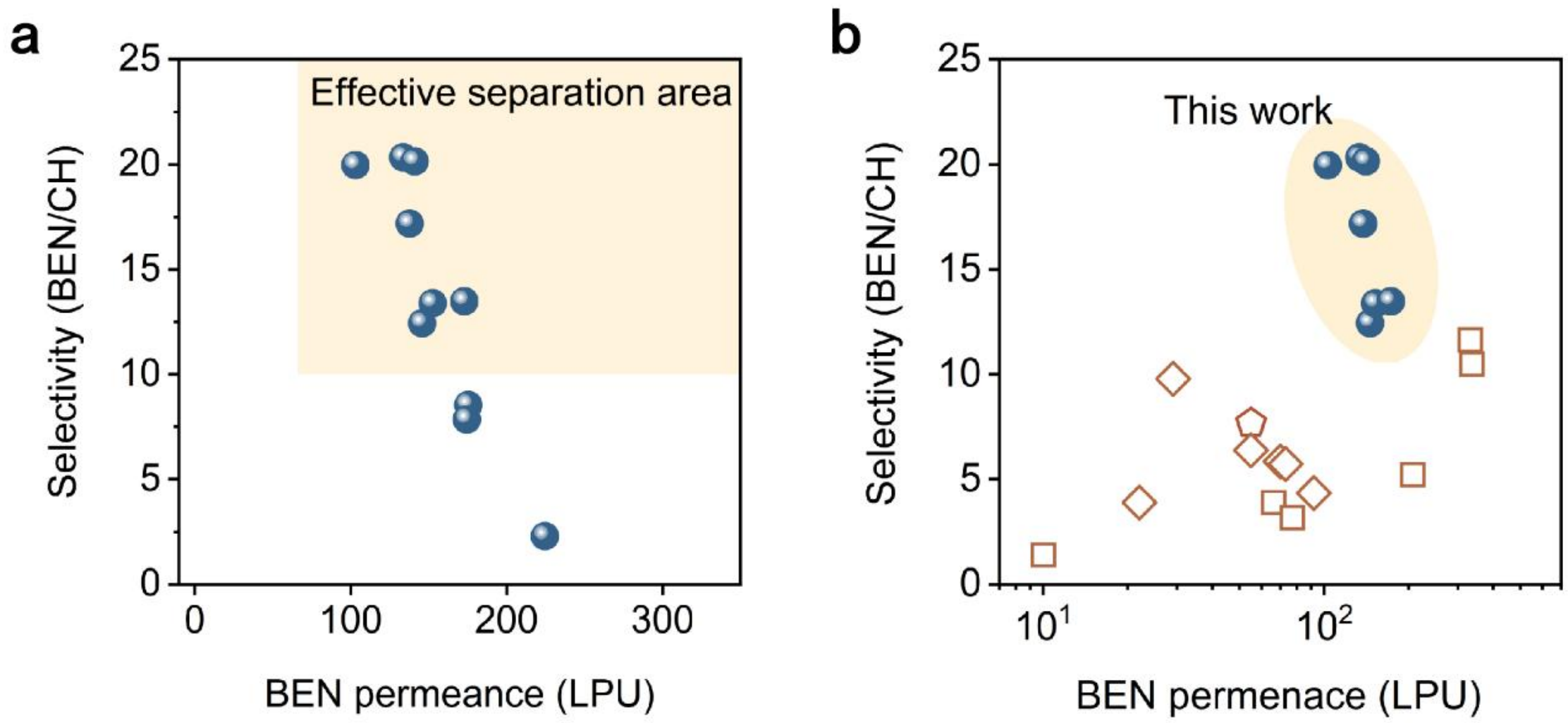

Fig. S29. Reproducibility of the 60-min TA-treated membrane for 50/50 BEN/CH separation (a) and performance comparison with literature (b).

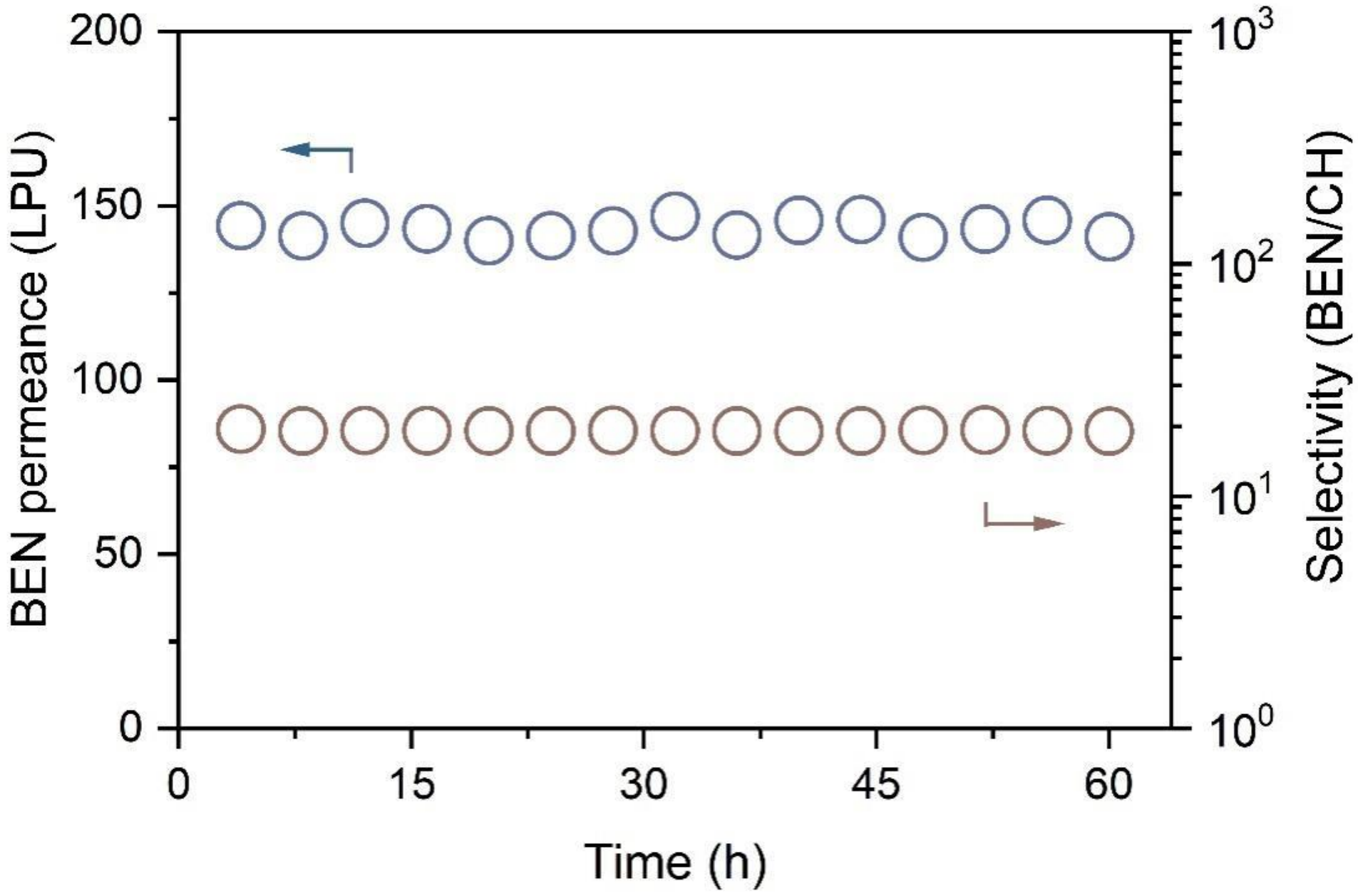

Fig. S30. Long-term stability of the 60-min TA-treated membrane for 50/50 BEN/CH.

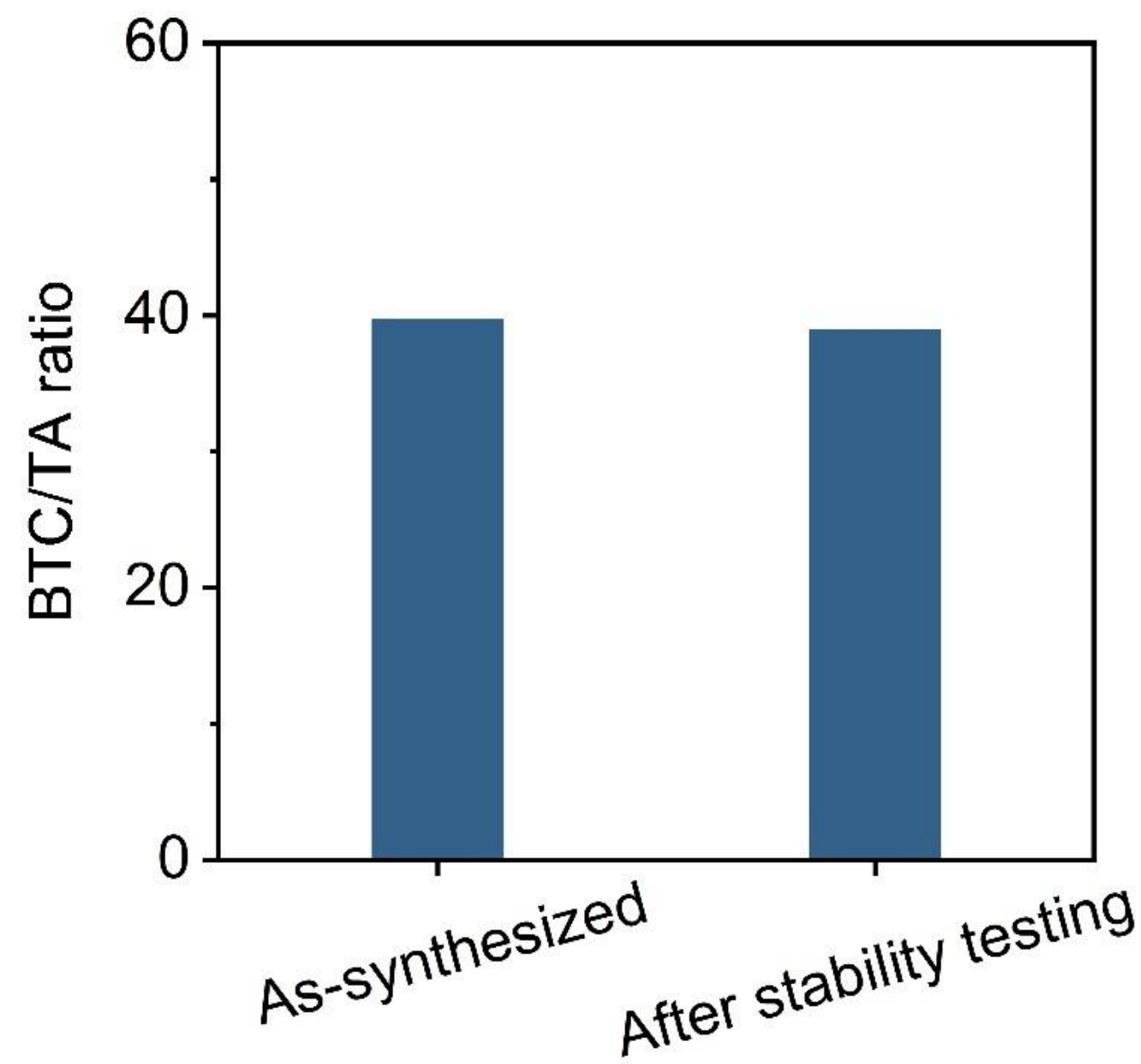

Fig. S31. The ratio of BTC/TA in the 60-min TA-treated membrane before and after stability testing

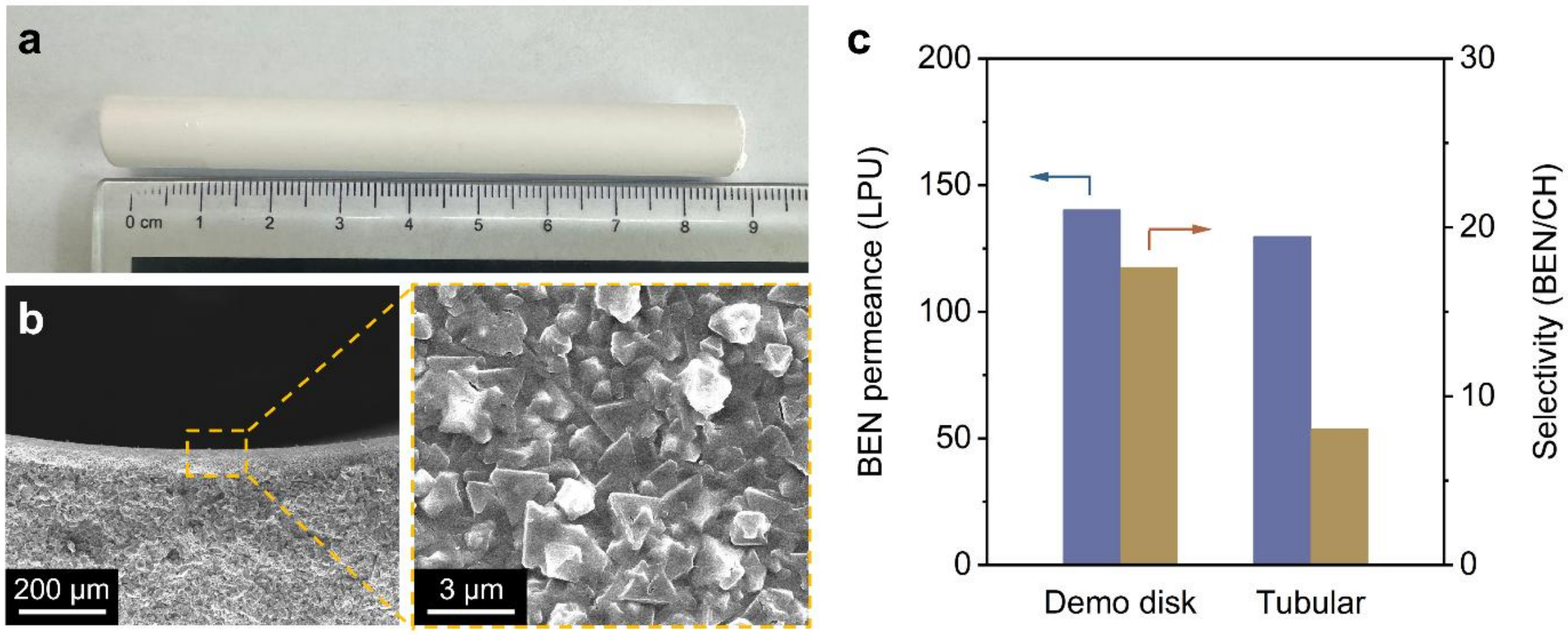

Fig. S32. Fabrication and separation performance of the tubular CuBTC membrane after 60-min TA treatment. (a) Digital photograph of the membrane fabricated on a tubular α-Al_2O_3 support. (b) Top-view and cross-sectional SEM images of the tubular membrane. (c) Comparison of the BEN/CH separation performance between the tubular membrane and the demo disk membrane under identical vapor permeation conditions.

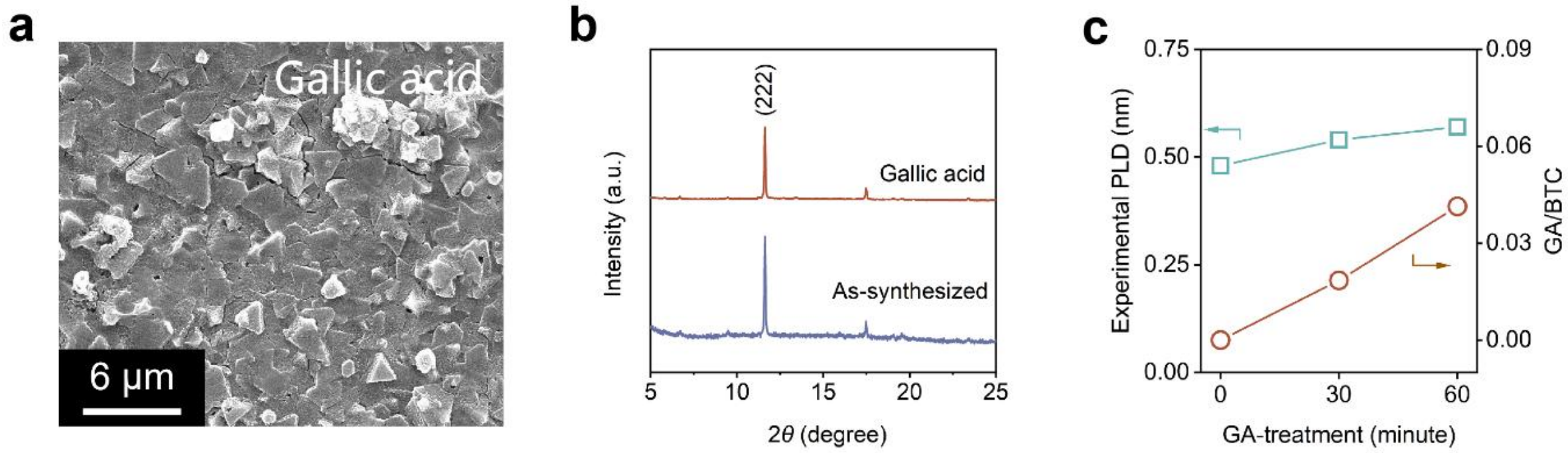

Fig. S33. Characterization of CuBTC membranes treated with 680 ppm gallic acid for 60 min. (a) Top-view SEM image. (b) PXRD patterns. (c) Experimental PLD and gallic acid/BTC ratio of CuBTC membranes as a function of treatment time.

After 60 min of treatment, SEM images revealed that the overall octahedral morphology of the membrane remained well preserved (Fig. S33a). The PXRD patterns retained the characteristic diffraction peaks of CuBTC after treatment (Fig. S33b), confirming that the crystalline framework remained largely intact throughout the etching process. As shown in Fig. S33c, the experimental PLD gradually increased with treatment time and reached 0.57 nm after 60 min. Simultaneously, the gallic acid/BTC ratio increased to 4.14%, indicating successful incorporation of gallic acid into the framework (Fig. S33c). These results demonstrate that gallic acid can effectively induce controlled framework reconstruction and pore enlargement while maintaining the structural integrity of the membrane, further supporting the general applicability of the polyphenol-assisted pore tuning strategy.

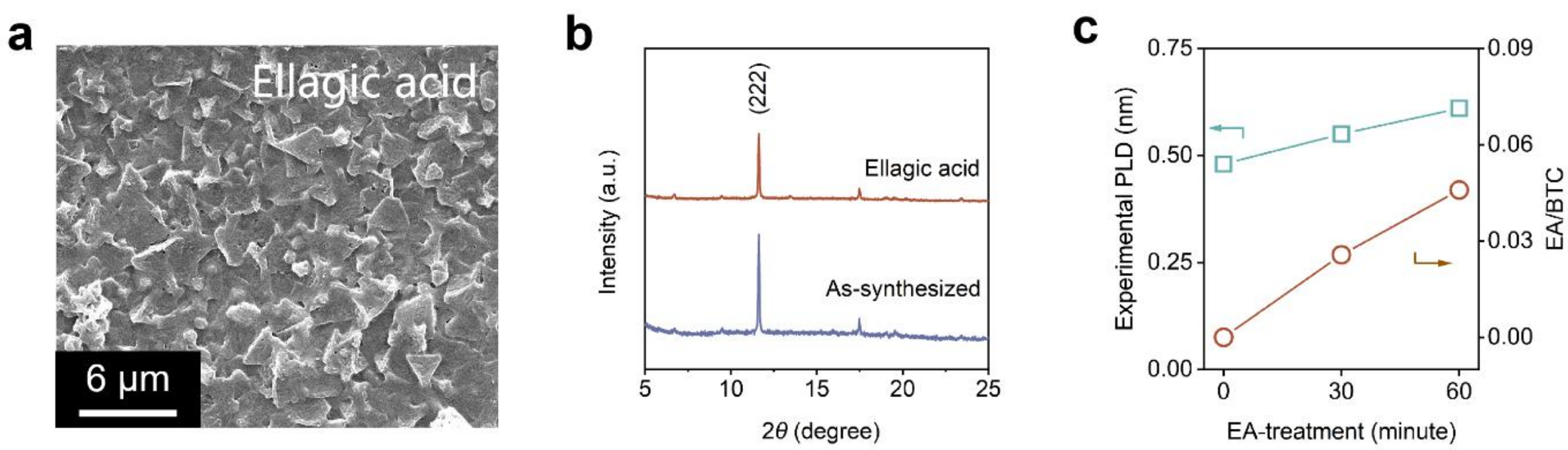

Fig. S34 Characterization of CuBTC membranes treated with 680 ppm ellagic acid for 60 min. (a) Top-view SEM image. (b) PXRD patterns. (c) Experimental PLD and ellagic acid/BTC ratio of CuBTC membranes as a function of treatment time.

Following treatment in a 680 ppm ellagic acid aqueous solution, the characteristic octahedral morphology of the membrane crystals was largely preserved (Fig. S34a). The PXRD patterns of the treated membranes remained dominated by the characteristic reflections of CuBTC (Fig. S34b), indicating that the long-range crystalline order was maintained during the modification process. As treatment time increased, the experimental PLD continuously expanded from 0.48 nm for the pristine membrane to 0.61 nm after 60 min of treatment, accompanied by an increase in the ellagic acid/BTC ratio to 4.59% (Fig. S34c). The larger PLD enlargement compared with that obtained using gallic acid suggests that ellagic acid can more effectively promote pore enlargement and functional molecule incorporation.

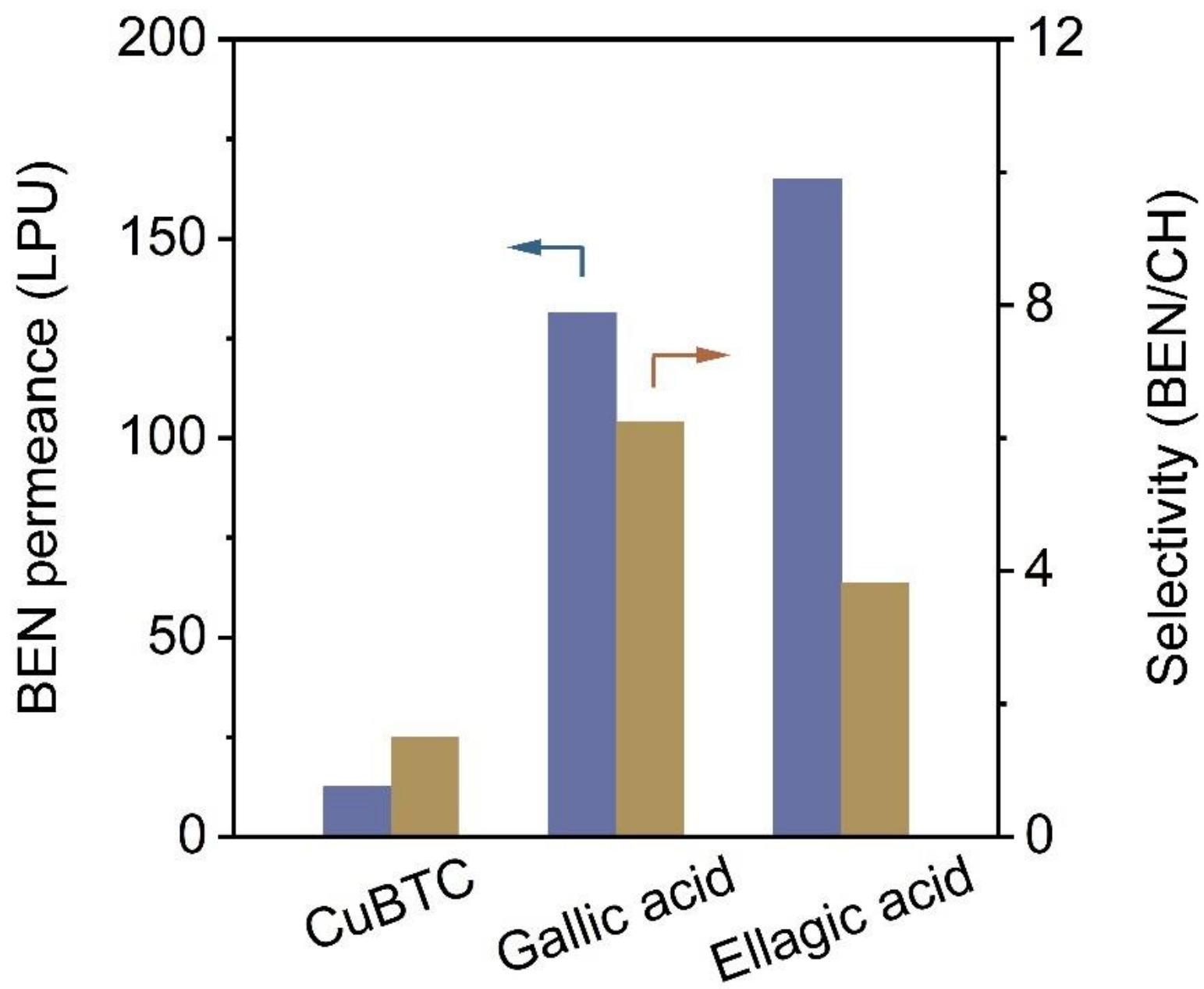

Fig. S35. Comparison of BEN/CH separation performance of CuBTC membrane and CuBTC membranes after treatment with other polyphenolic compounds.

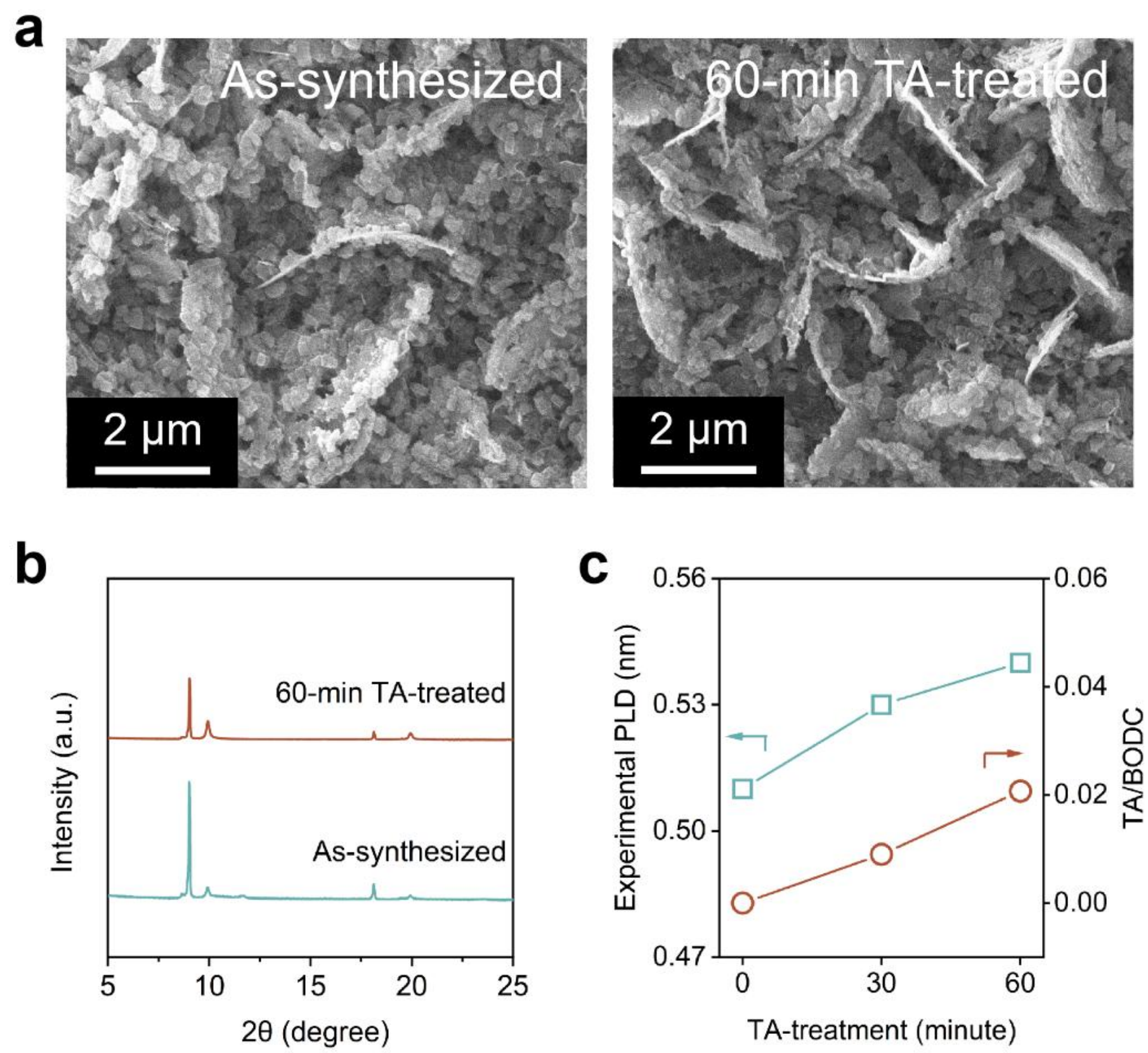

Fig. S36. Extension of the TA-treatment to Zn-BODC membranes, with the TA concentration at 680 ppm and duration of 60 min. (a) Top-view SEM image of the as-synthesized Zn-BODC membrane. (b) Top-view SEM image of the Zn-BODC membrane after treatment. (c) PXRD patterns of the as-synthesized and TA-treated Zn-BODC membranes. (d) Experimental PLD and TA/BODC ratio of Zn-BODC membranes as a function of TA treatment time.

To further evaluate the applicability of the TA-assisted pore-engineering strategy to other MOF membranes, Zn-BODC membranes were subjected to treatment in a 680-ppm TA aqueous solution for different durations. As shown in Fig. S36a, the surface morphology of the Zn-BODC membrane remained largely unchanged after 60 min of treatment, indicating that the TA treatment did not induce significant crystal damage or membrane deterioration. The PXRD patterns of the treated membrane retained all characteristic diffractions of Zn-BODC (Fig. S36b), confirming that the crystalline framework was preserved during the modification process. Despite the maintained structural integrity, gradual pore modulation was achieved. The experimental PLD increased from 0.51 nm for the as-synthesized membrane to 0.53 nm after 60 min of treatment (Fig. S36c). Simultaneously, the TA/BODC ratio reached 2.07%, demonstrating successful incorporation of TA into the framework. These results indicate that mild TA treatment can be extended to other MOF membranes, enabling controllable adjustment of pore structure and chemical functionality while maintaining framework stability.

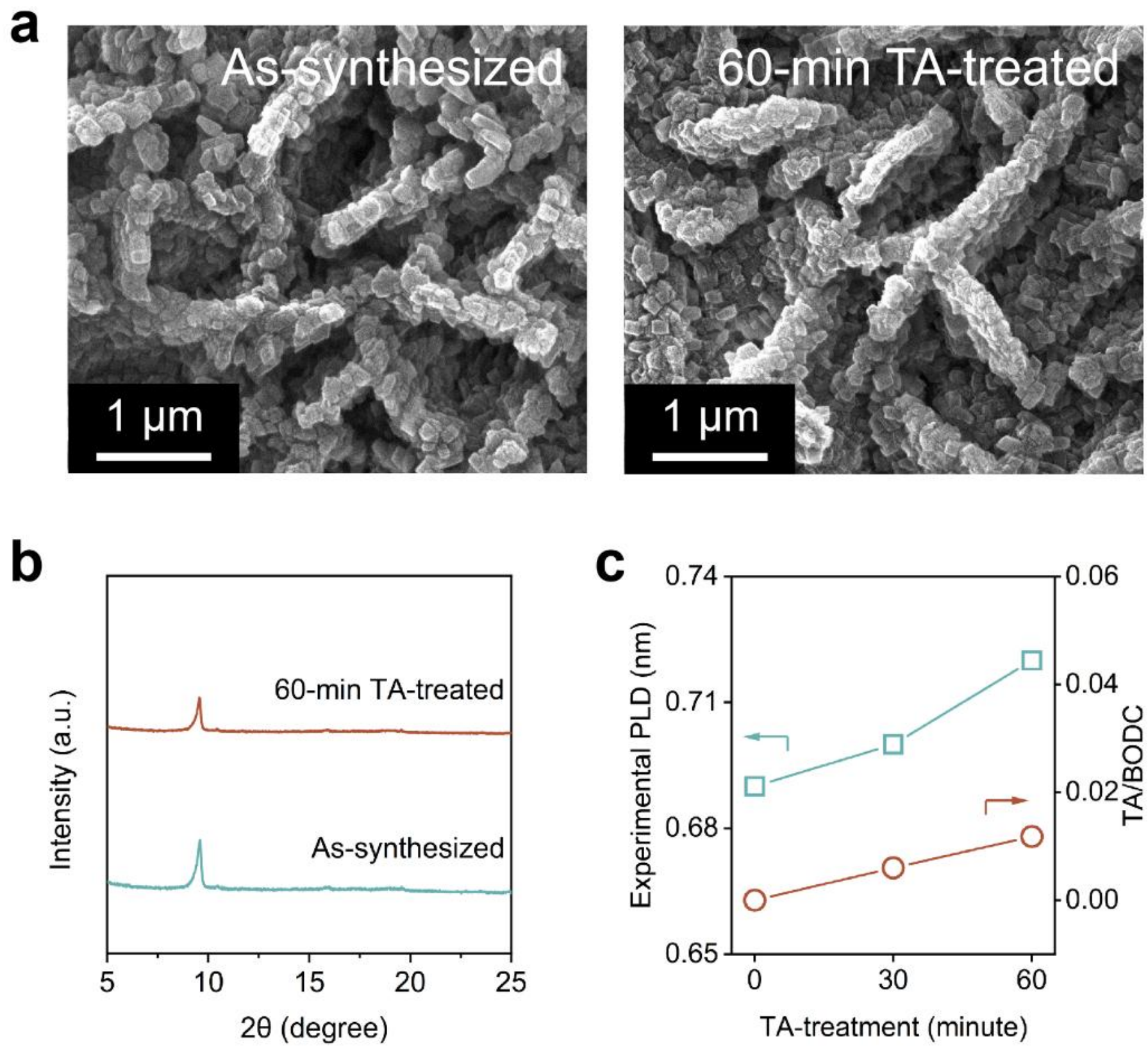

Fig. S37. Extension of the TA-treatment to Al-BODC membranes, with the TA concentration at 680 ppm and duration of 60 min. (a) Top-view SEM image of the as-synthesized Al-BODC membrane. (b) Top-view SEM image of the Al-BODC membrane after treatment. (c) PXRD patterns of the as-synthesized and TA-treated Al-BODC membranes. (d) Experimental PLD and TA/BODC ratio of Al-BODC membranes as a function of TA treatment time.

The versatility of the TA-assisted modification strategy was further examined using Al-BODC membranes, which possess a substantially larger intrinsic pore aperture than CuBTC and Zn-BODC. As shown in Fig. S37a, the membrane surface retained its original morphology after treatment with a 680-ppm TA aqueous solution for 60 min, indicating that the mild etching process did not compromise membrane continuity or crystal packing. Structural characterization by PXRD revealed that the characteristic diffraction peaks of Al-BODC were preserved after treatment (Fig. S37b), demonstrating that the framework remained crystalline throughout the modification process. Meanwhile, the experimental PLD gradually increased from 0.69 nm to 0.72 nm with increasing treatment time (Fig. S37c), accompanied by a TA/BODC ratio of 1.18% after 60 min. Although the degree of TA incorporation was lower than that observed for CuBTC and Zn-BODC, measurable pore expansion was still achieved. These findings suggest that the TA-assisted strategy is applicable to MOF membranes with different metal nodes and pore architectures, providing a general route for fine-tuning membrane pore size while preserving structural integrity.

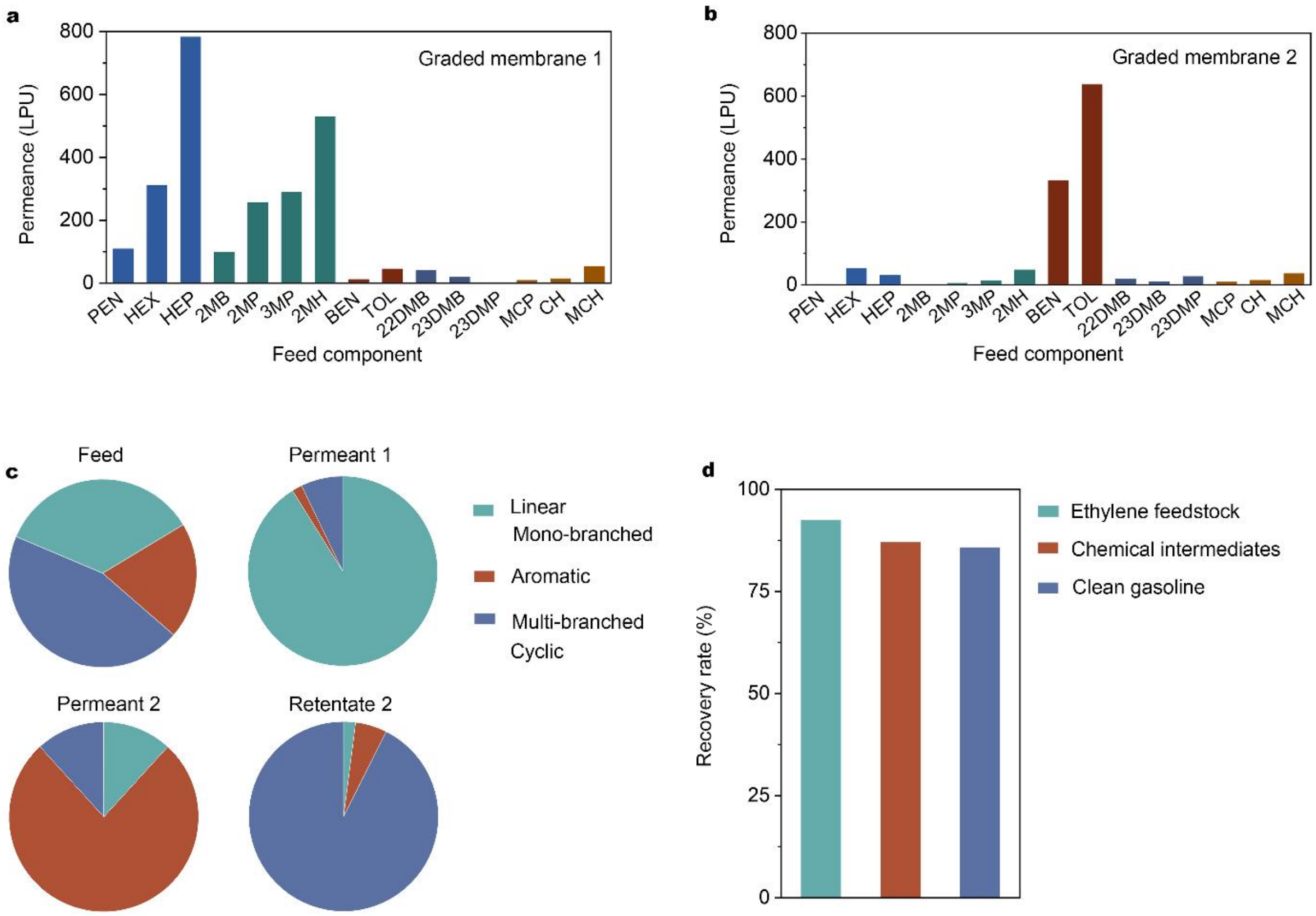

Fig. S38. Graded sorting cascade for light naphtha refining at 353 K. (a) Separation data for graded sorting stage. The feed solution consisted of PEN/HEX/HEP/2MB/2MP/3MP/2MH/22DMB/23DMB/23DMP (each 5 wt.%) and BEN/TOL/MCP/CH/MCH (each 10 wt.%). (b) Separation data for graded sorting stage 2. (c) Compositions of the raw feed and products from each separation stage. (d) Recovery rates of the ethylene feedstock, chemical intermediates, and clean gasoline.

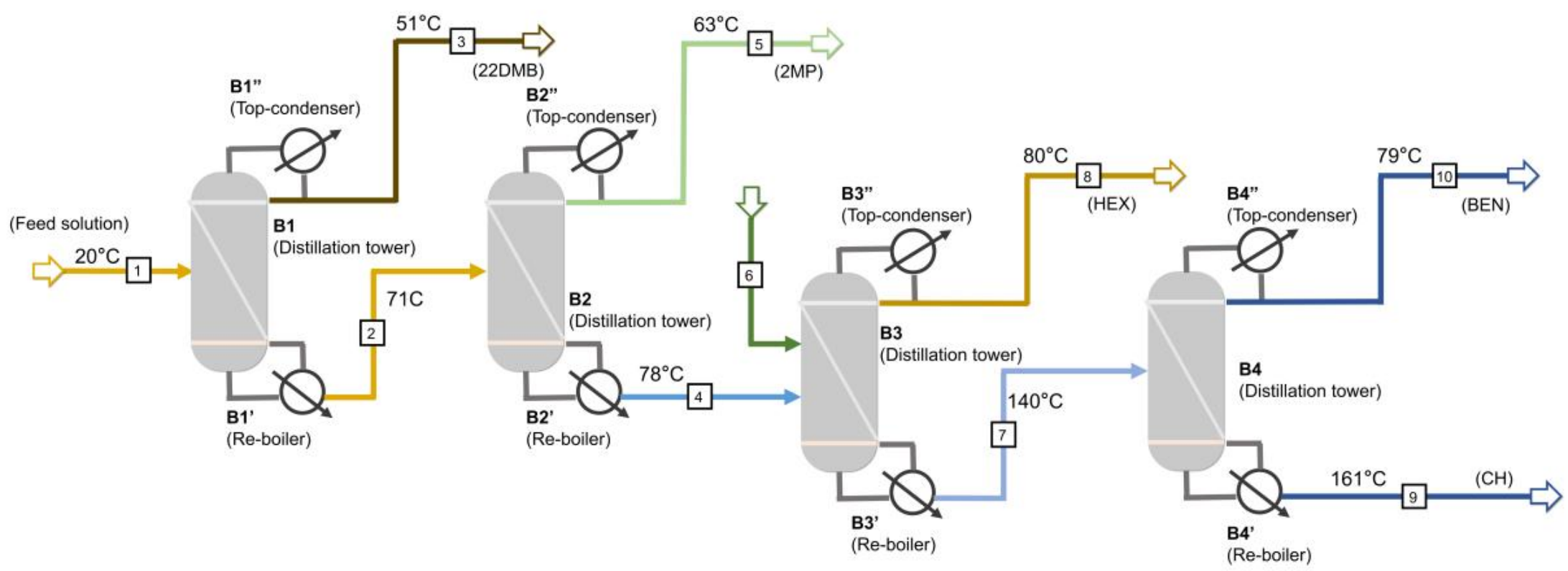

		1	2	3	4	5	6	7	8	9	10
Process parameters	Temperature (°C)	20	71	51	78	63	80	140	80	161	79
	Pressure (kPa)	405	105	102	105	102	300	105	101	100	100
	Mass flow rate (kg h^{-1})	1000	847	153	495	352	3000	495	300	3000	195
Energy consumption statistics		Q1	Q2	Q3	Q4	Q5	Q6	Q7	Q8	Q	
	Energy consumption (GJ per ton of feed solution)	0.569	0.477	0.951	0.952	0.629	0.280	0.314	0.268	4.44	

Fig. S39. Process schematic for separating a representative five-component mixture (HEX/2MP/BEN/22DMB/CH = 15/20/20/15/30) using vacuum distillation.

A process simulation was carried out using Aspen Plus v7.2 to evaluate the energy consumption of a conventional distillation for a representative five-component mixture (HEX/2MP/BEN/22DMB/CH = 15/20/20/15/30, by mass) at an annual processing capacity of eight thousand tons. The feed stream, set at 20 °C and 1000 kg h^{-1}, was first introduced into distillation column B1 for vacuum distillation, where 22DMB was recovered from the overhead at 102 kPa and 51 °C. The bottom stream was subsequently fed into column B2, yielding 2MP at the overhead under 102 kPa and 63 °C. The bottom product from B2 entered column B3, where HEX was separated at the overhead at 101 kPa and 80 °C. The remaining mixture was then processed in column B4 through extractive distillation, enabling the separation of BEN from the overhead at 100 kPa and 79 °C, while CH was retained in the bottom fraction.

The heat duties of the reboilers (B1', B2', B3', B4') were defined as Q1, Q3, Q5, and Q7, respectively, and the cooling duties of the condensers (B1'', B2'', B3'', B4'') were designated as Q2, Q4, Q6, and Q8. Irrespective of additional electricity consumption during vacuum operation, the total energy demand of the overall separation process is expressed as: Q = Q1 + Q2 + Q3 + Q4 + Q5 + Q6 + Q7 + Q8.

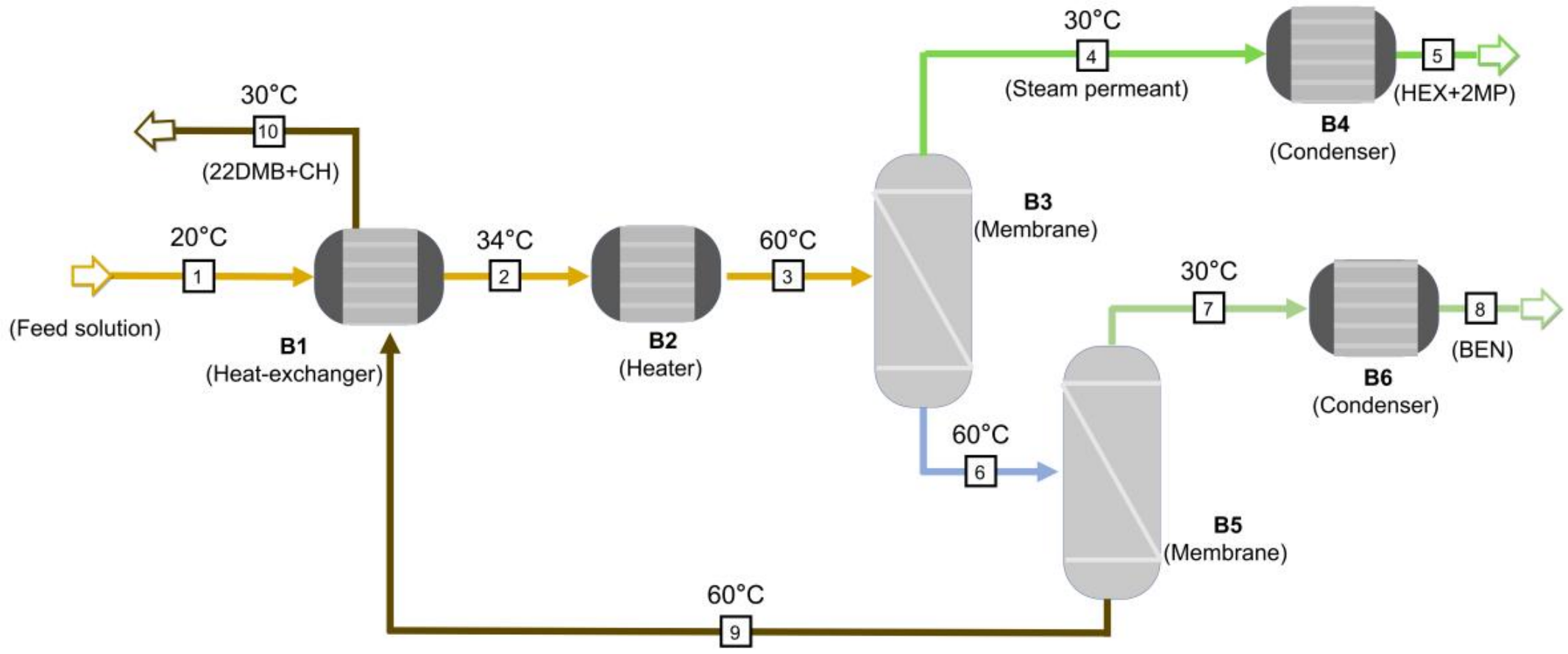

		1	2	3	4	5	6	7	8	9	10
Process parameters	Temperature (°C)	20	34	60	30	30	60	30	-14	60	30
	Pressure (kPa)	405	405	200	2	30	405	2	2	405	405
	Mass flow rate (kg h^{-1})	1250	1250	1250	428	428	822	243	243	1250	1250

		Q1	Q2	Q3	Q4	Q5	Q
Energy consumption	Energy consumption (GJ per ton of feed solution)	0.048	0.085	0.106	0.061	0.084	0.385

Fig. S40. Process schematic for separating a representative five-component mixture (HEX/2MP/BEN/22DMB/CH = 15/20/20/15/30) using a graded membrane-based separation strategy.

A process simulation was carried out using Aspen Plus v7.2 to evaluate the energy consumption of a graded sorting cascade process for a representative five-component mixture (HEX/2MP/BEN/22DMB/CH = 15/20/20/15/30, by mass) at an annual processing capacity of eight thousand tons. To reflect laboratory operating conditions, the feed temperature was set to 20 °C. The feed stream was preheated to 60 °C through a two-stage heating system (B1 and B2) before entering the first membrane module (B3). The permeate-side absolute pressure was maintained at 2 kPa, and the resulting permeate vapor was condensed and collected in the low-temperature condenser B4 at 30 °C.

The retentate from the first membrane stage was subsequently fed into a second membrane module (B5), where the permeate-side pressure was also maintained at 2 kPa to enable further selective separation. The vapor permeant generated in this stage was condensed at 30 °C in condenser B6. The total energy demand for vapor-permeation separation consists of three components: the heating duty required for feed preheating in B1 (Q1); the latent heat associated with vapor generation in membrane modules B3 and B5 (Q2 and Q4); and the cooling duty required for permeate condensation in B4 and B6 (Q3 and Q5). Accordingly, the overall energy consumption, excluding auxiliary electricity usage, is expressed as: Q = Q1 + Q2 + Q3 + Q4 + Q5.

Table S26. Cost estimation for separating a representative five-component mixture (HEX/2MP/BEN/22DMB/CH = 15/20/20/15/30, by mass) using a conventional vacuum distillation process.

Module		Annual operating cost (10^4 RMB·year^{-1})
B1	Reboiler heat duty	6.71
	Condenser cooling duty	0.63
B2	Reboiler heat duty	11.2
	Condenser cooling duty	1.25
B3	Reboiler heat duty	7.43
	Condenser cooling duty	0.37
B4	Reboiler heat duty	3.70
	Condenser cooling duty	0.35
Total		31.7

A process simulation and energy consumption analysis of the conventional continuous vacuum distillation process were performed using Aspen Plus v7.2. A representative five-component mixture (HEX/2MP/BEN/22DMB/CH = 15/20/20/15/30, by mass) was used as the feed, with a processing capacity of 8000 t year^{-1} (1000 kg h^{-1}) and an annual operating time of 8000 h. The operating cost was defined as the energy cost required to maintain continuous operation of the separation process and was mainly determined by the energy consumption associated with heating, cooling, and phase-change operations. For the vacuum distillation process, the operating cost consisted primarily of the reboiler heat duties and condenser cooling duties of distillation modules B1–B4. Based on the energy consumption of each unit, the total annual operating cost of the vacuum distillation process was estimated to be RMB 316,700 year^{-1}.

Table S27. Cost estimation for separating a representative five-component mixture (HEX/2MP/BEN/22DMB/CH = 15/20/20/15/30, by mass) using the graded membrane cascade strategy.

Module		Annual operating cost (10^4 RMB·year^{-1})
B2	Heating duty	0.57
B3	Pervaporation heating duty	1.00
	Vacuum condensation cooling duty	0.14
B5	Pervaporation heating duty	0.72
	Vacuum condensation cooling duty	1.73
Total		3.86

A process simulation and energy consumption analysis of the graded membrane cascade were also carried out using Aspen Plus v7.2. The feed composition, processing capacity, and product specifications were identical to those used for the vacuum distillation process. For the membrane-based process, the operating cost was defined as the energy cost required to sustain vapor permeation and was mainly influenced by feed preheating, vapor generation, vacuum maintenance, and permeate condensation. Specifically, the operating cost included the feed heating duty in module B2 and the heating and vacuum condensation duties associated with the vapor permeation units in modules B3 and B5. The total annual operating cost of the graded membrane cascade was estimated to be RMB 38,600 year^{-1}.

Reference

1. Xiao J and Wei J, Diffusion mechanism of hydrocarbons in zeolites—I. Theory. *Chem Eng Sci* 1992; **47**: 1123–41.
2. Shelekhin A, Dixon A and Ma Y. Theory of gas diffusion and permeation in inorganic molecular-sieve membranes. *AIChE J* 1995; **41**: 58–67.
3. Lee H, Kanezashi M and Shimomura Y *et al*. Evaluation and fabrication of pore-size-tuned silica membranes with tetraethoxydimethyl disiloxane for gas separation. *AIChE J* 2011; **57**: 2755–65.
4. Han S, Ciufo R and Meyerson M *et al*. Solvent-free vacuum growth of oriented HKUST-1 thin films. *J Mater Chem A* 2009; **7**: 19396–406.
5. Liu P, Cai K and Wang K *et al*. Highly defective HKUST-1 with excellent stability and SO_2 uptake: the hydrophobic armor effect of functionalized ionic liquids. *Green Energy Environ* 2024; **9**: 1711–23.
6. Coronas J, Noble R and Falconer J. Separations of C_4 and C_6 isomers in ZSM-5 tubular membranes. *Ind Eng Chem Res* 1998; **37**: 166.
7. Funke H, Argo A and Falconer J *et al*. Separations of cyclic, branched, and linear hydrocarbon mixtures through silicalite membranes. *Ind Eng Chem Res* 1997; **36**: 137–43.
8. Yu J, Zhang P and Ding J *et al*. UiO-66/PIM-1 mixed-matrix membrane for hexane isomer separation. *Inorg Chem* 2024; **63**: 13031–8.
9. Yang W, Yang X and Wang Y *et al*. Pervaporation separation of C_6 alkane isomers by Al-bttotb membrane. *J Membr Sci* 2022; **661**: 120916.
10. Wu T, Wang N and Li J *et al*. Tubular thermal crosslinked-PEBA/ceramic membrane for aromatic/aliphatic pervaporation. *J Membr Sci* 2015; **486**: 1–9.
11. Ye H, Li J and Lin Y *et al*. Synthesis of polyimides containing fluorine and their pervaporation performances to aromatic/aliphatic hydrocarbon mixtures. *J Macromol Sci Part A Pure Appl Chem* 2008; **45**: 172–8.
12. An Q, Qian J and Zhao Q *et al*. Polyacrylonitrile-block-poly(methyl acrylate) membranes 2: Swelling behavior and pervaporation performance for separating benzene/cyclohexane. *J Membr Sci* 2008; **313**: 60–7.
13. Maji S and Banerjee S. Synthesis and characterization of new meta connecting semifluorinated poly(ether amide)s and their pervaporation properties for benzene/cyclohexane mixtures. *J Membr Sci* 2010; **360**: 380–8.
14. Maji S, Banerjee S and Pradhan N. Separation of benzene/cyclohexane mixture using semifluorinated aromatic poly(ether amide) membranes with and without cardo unit in the main chain. *Separ Purif Tech* 2009; **70**: 128–35.
15. Maji S and Banerjee S. Preparation of new semifluorinated aromatic poly(ether amide)s and evaluation of pervaporation performance for benzene/cyclohexane 50/50 mixture. *J Membr Sci* 2010; **349**: 145–55.

16. Xi T, Lu Y and Ai X *et al*. Ionic liquid copolymerized polyurethane membranes for pervaporation separation of benzene/cyclohexane mixtures. *Polymer* 2019; **185**: 121948.

17. Wang N, Wu T and Wang L *et al*. Hyperbranched polymer composite membrane using water as solvent for separating aromatic/aliphatic hydrocarbon mixtures. *Separ Purif Tech* 2017; **179**: 225–35.

18. Zhang L, Wang L and Wang N *et al*. Covalent organic frameworks hybird membrane with optimized mass transport nanochannel for aromatic/aliphatic mixture pervaporation. *J Membr Sci* 2020; **598**: 117652.

19. Dubey V, Pandey L and Saxena C. Pervaporation of benzene/cyclohexane mixtures through supramolecule containing poly(vinyl acetal) membranes. *Separ Purif Tech* 2006; **50**: 45–50.

20. Shen J, Chu Y and Ruan H *et al*. Pervaporation of benzene/cyclohexane mixtures through mixed matrix membranes of chitosan and Ag^{+}/carbon nanotubes. *J Membr Sci* 2014; **462**: 160–9.

21. Liu L, Wang N and Liu H *et al*. Nano-array assisted metalorganic polyhedra membranes for the pervaporation of aromatic/aliphatic mixtures. *J Membr Sci* 2019; **575**: 1–8.

22. Zhou J, Yu G and Li Q *et al*. Separation of benzene and cyclohexane by nonporous adaptive crystals of a hybrid[3]arene. *J Am Chem Soc* 2020; **142**: 2228–32.
